# Supplementary material for: Proteomic and Transcriptomic Responses of the Desiccation-Tolerant Moss Racomitrium canescens in the Rapid Rehydration Processes
Source: Genes (Basel). 2023 Feb 2;14(2):390. doi: 10.3390/genes14020390 (PMC9956249; doi:10.3390/genes14020390)
Supplement: Supplementary file 1 [file genes-14-00390-s001.zip › figure S1.pptx]

## Slide 1
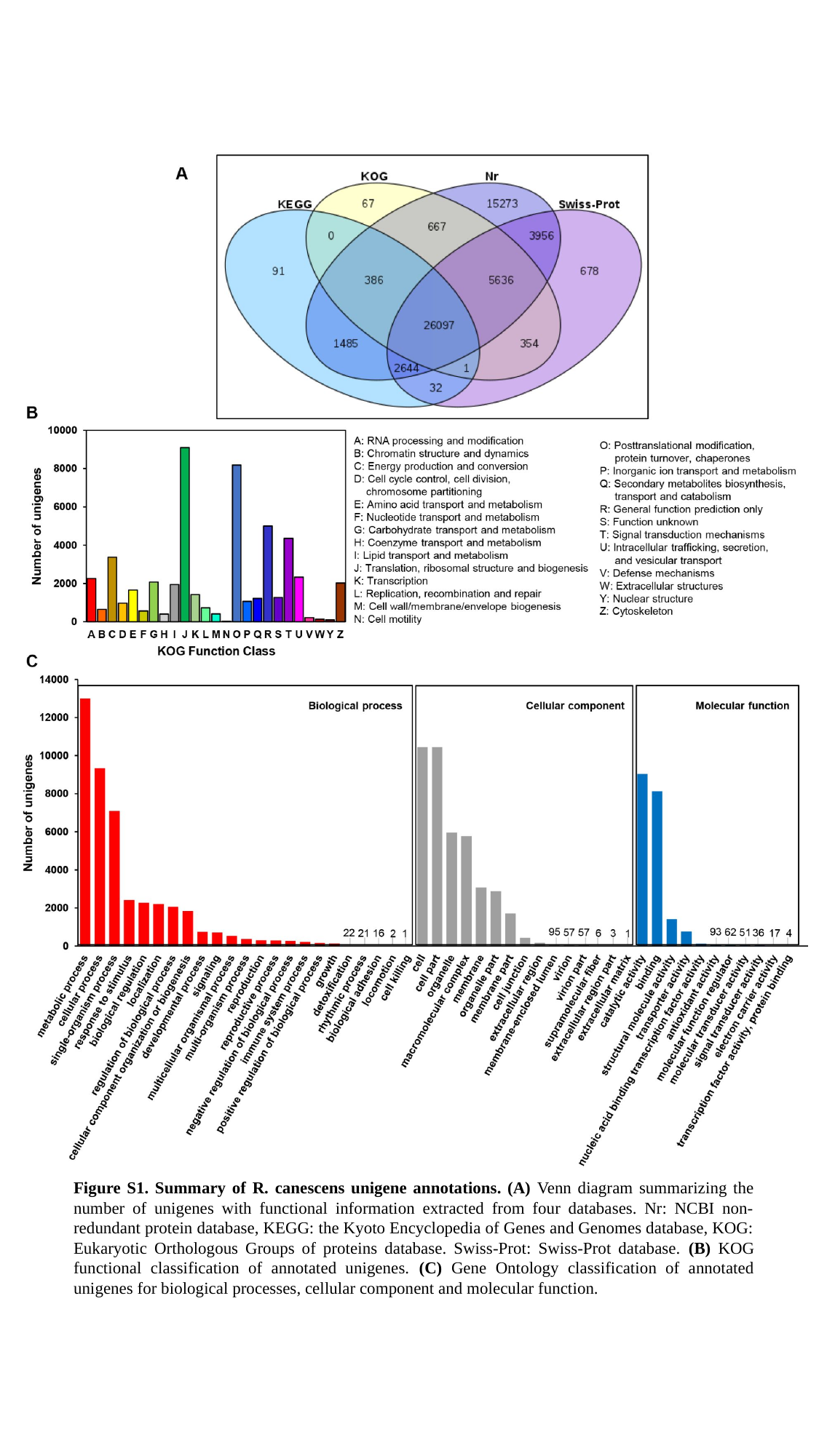

Figure S1. Summary of R. canescens unigene annotations. (A) Venn diagram summarizing the number of unigenes with functional information extracted from four databases. Nr: NCBI non-redundant protein database, KEGG: the Kyoto Encyclopedia of Genes and Genomes database, KOG: Eukaryotic Orthologous Groups of proteins database. Swiss-Prot: Swiss-Prot database. (B) KOG functional classification of annotated unigenes. (C) Gene Ontology classification of annotated unigenes for biological processes, cellular component and molecular function.
