# Supplementary material for: Proteomic and Transcriptomic Responses of the Desiccation-Tolerant Moss Racomitrium canescens in the Rapid Rehydration Processes
Source: Genes (Basel). 2023 Feb 2;14(2):390. doi: 10.3390/genes14020390 (PMC9956249; doi:10.3390/genes14020390)
Supplement: Supplementary file 1 [file genes-14-00390-s001.zip › figure S10 .pptx]

## Slide 1
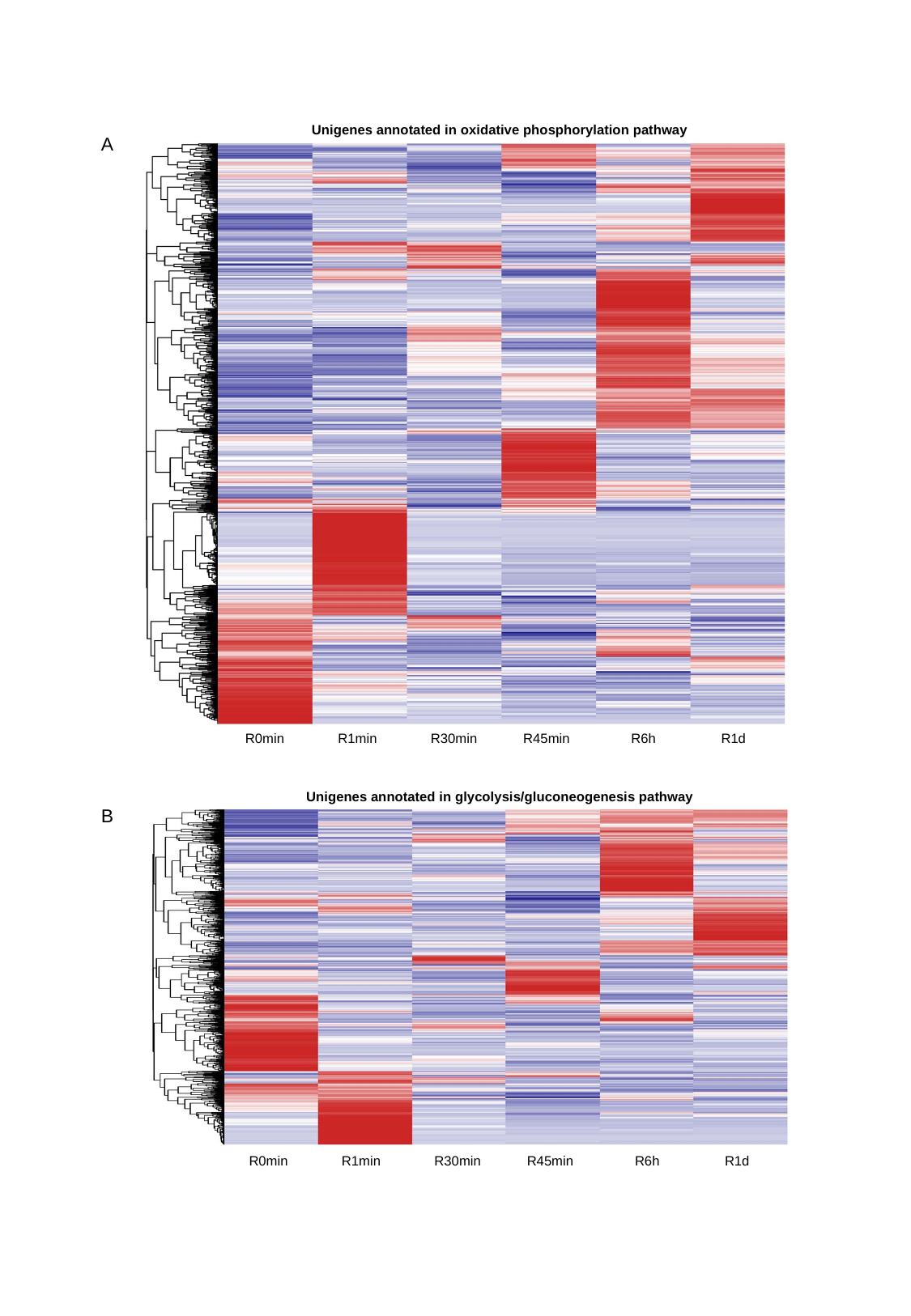

Unigenes annotated in oxidative phosphorylation pathway
A
R0min R1min R30min R45min R6h R1d
Unigenes annotated in glycolysis/gluconeogenesis pathway
B
R0min R1min R30min R45min R6h R1d

## Slide 2
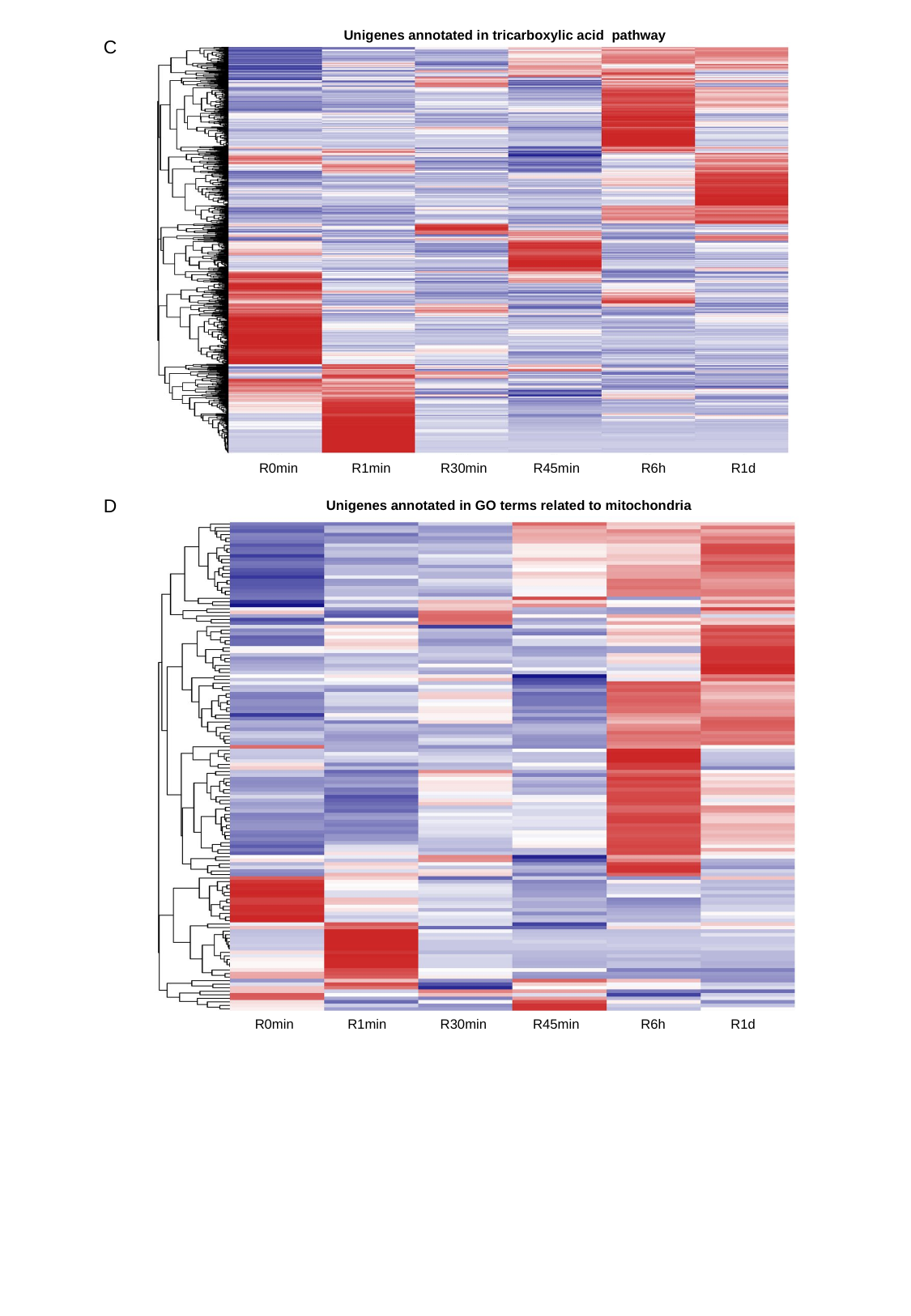

Unigenes annotated in tricarboxylic acid pathway
C
R0min R1min R30min R45min R6h R1d
D
Unigenes annotated in GO terms related to mitochondria
R0min R1min R30min R45min R6h R1d

## Slide 3
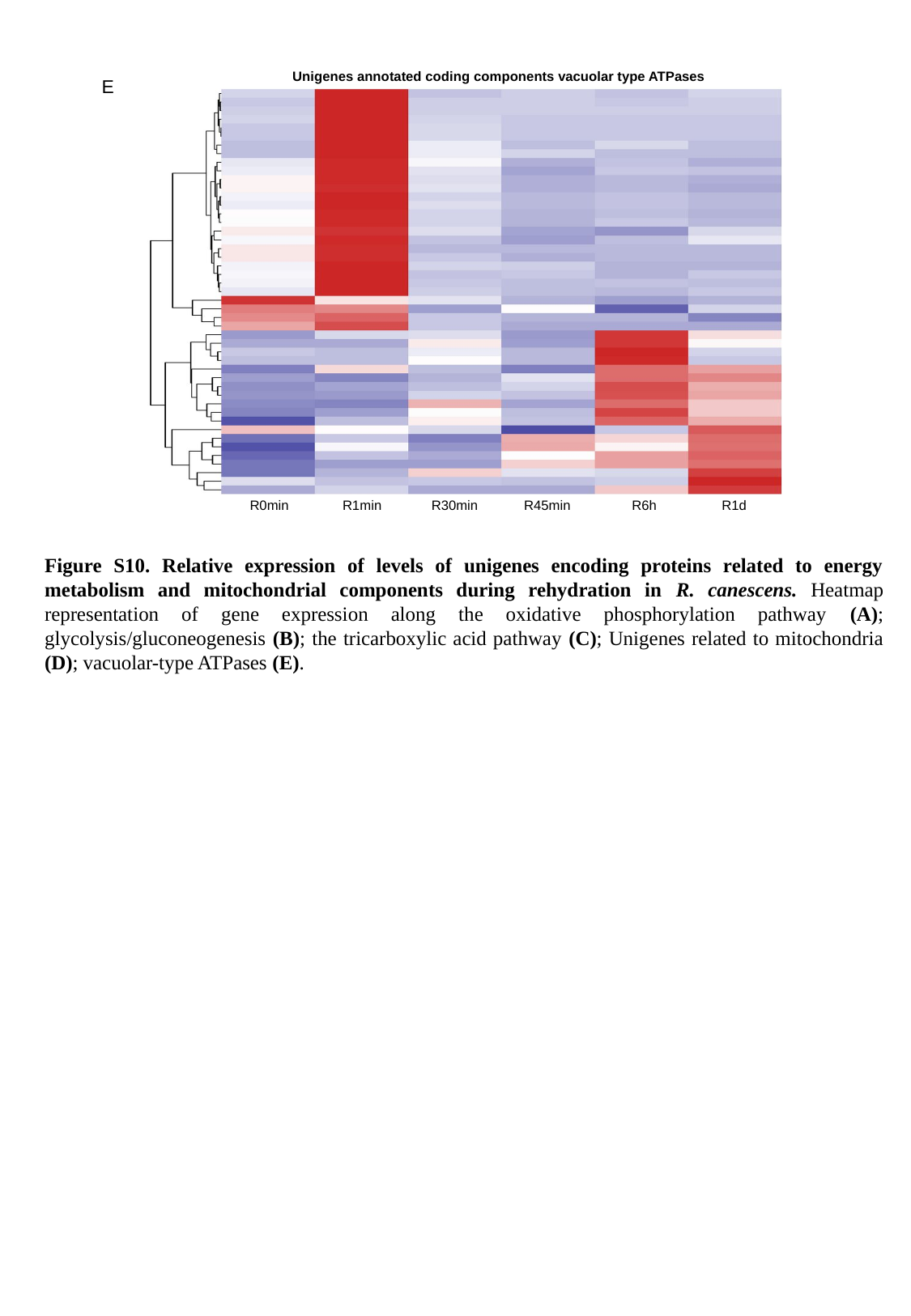

Unigenes annotated coding components vacuolar type ATPases
E
R0min R1min R30min R45min R6h R1d
Figure S10. Relative expression of levels of unigenes encoding proteins related to energy metabolism and mitochondrial components during rehydration in R. canescens. Heatmap representation of gene expression along the oxidative phosphorylation pathway (A); glycolysis/gluconeogenesis (B); the tricarboxylic acid pathway (C); Unigenes related to mitochondria (D); vacuolar-type ATPases (E).
