# Supplementary material for: Proteomic and Transcriptomic Responses of the Desiccation-Tolerant Moss Racomitrium canescens in the Rapid Rehydration Processes
Source: Genes (Basel). 2023 Feb 2;14(2):390. doi: 10.3390/genes14020390 (PMC9956249; doi:10.3390/genes14020390)
Supplement: Supplementary file 1 [file genes-14-00390-s001.zip › figure S11.pptx]

## Slide 1
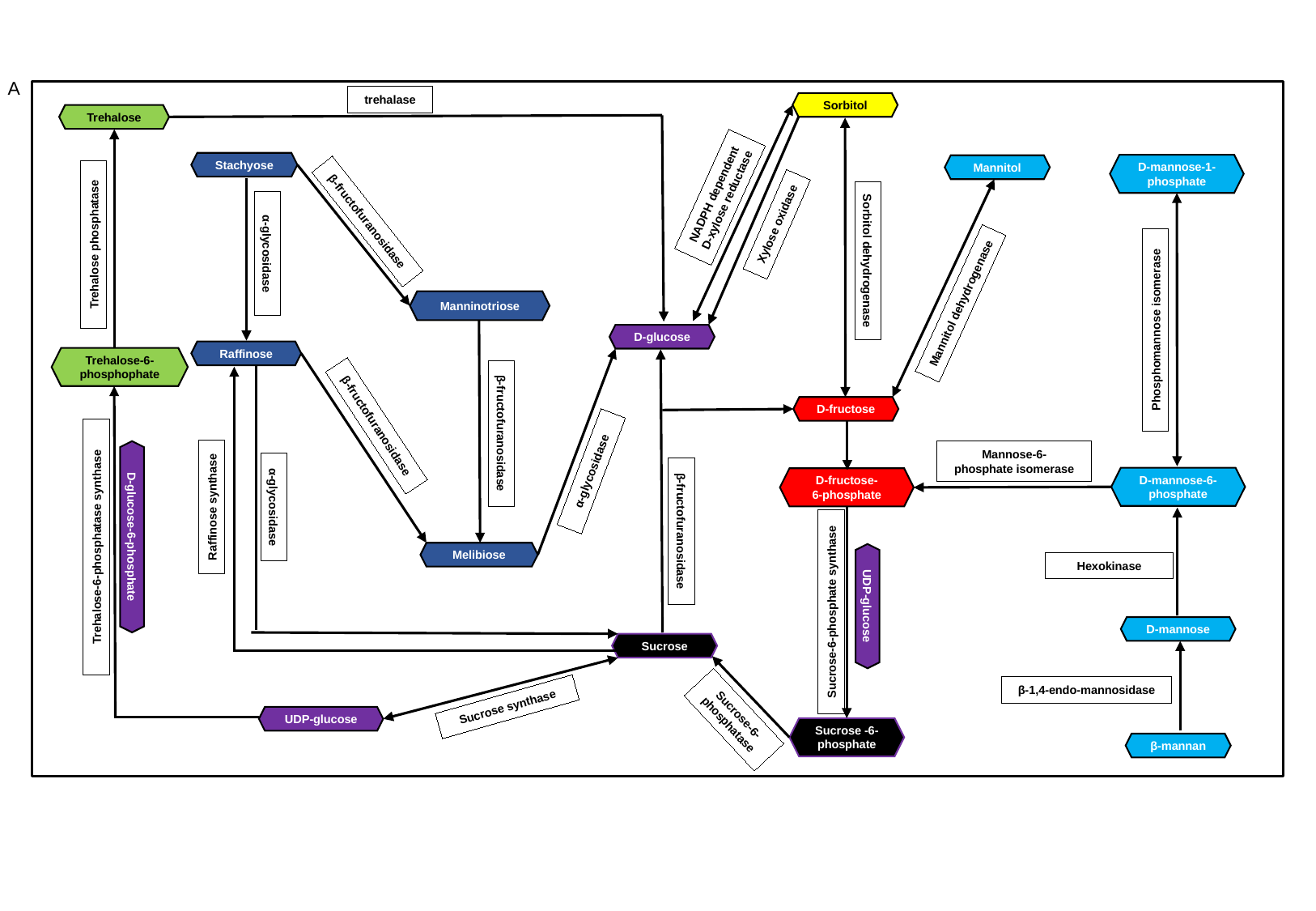

A
trehalase
Sorbitol
Trehalose
Stachyose
D-mannose-1-phosphate
Mannitol
NADPH dependent
D-xylose reductase
β-fructofuranosidase
Xylose oxidase
Trehalose phosphatase
α-glycosidase
Sorbitol dehydrogenase
Mannitol dehydrogenase
Manninotriose
Phosphomannose isomerase
D-glucose
Raffinose
Trehalose-6-phosphophate
D-fructose
β-fructofuranosidase
β-fructofuranosidase
Mannose-6-
phosphate isomerase
α-glycosidase
D-mannose-6-phosphate
D-fructose-
6-phosphate
α-glycosidase
Raffinose synthase
β-fructofuranosidase
D-glucose-6-phosphate
Trehalose-6-phosphatase synthase
Melibiose
Hexokinase
UDP-glucose
Sucrose-6-phosphate synthase
D-mannose
Sucrose
β-1,4-endo-mannosidase
Sucrose synthase
Sucrose-6-phosphatase
UDP-glucose
Sucrose -6-phosphate
β-mannan

## Slide 2
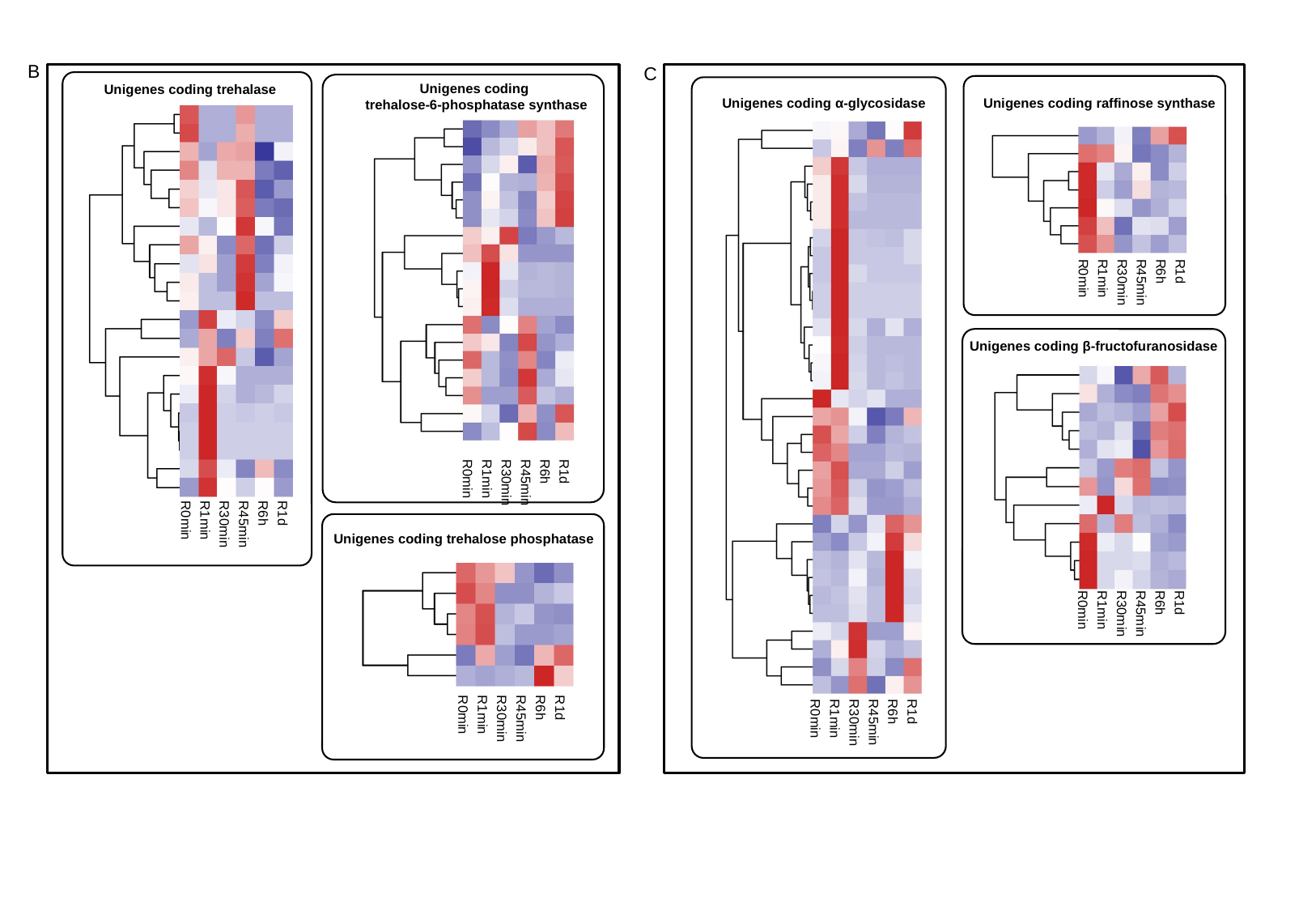

B
Unigenes coding trehalase
R1d
R6h
R45min
R30min
R1min
R0min
Unigenes coding
 trehalose-6-phosphatase synthase
R1d
R6h
R45min
R30min
R1min
R0min
Unigenes coding trehalose phosphatase
R1d
R6h
R45min
R30min
R1min
R0min
C
Unigenes coding raffinose synthase
R1d
R6h
R45min
R30min
R1min
R0min
Unigenes coding α-glycosidase
R1d
R6h
R45min
R30min
R1min
R0min
Unigenes coding β-fructofuranosidase
R1d
R6h
R45min
R30min
R1min
R0min

## Slide 3
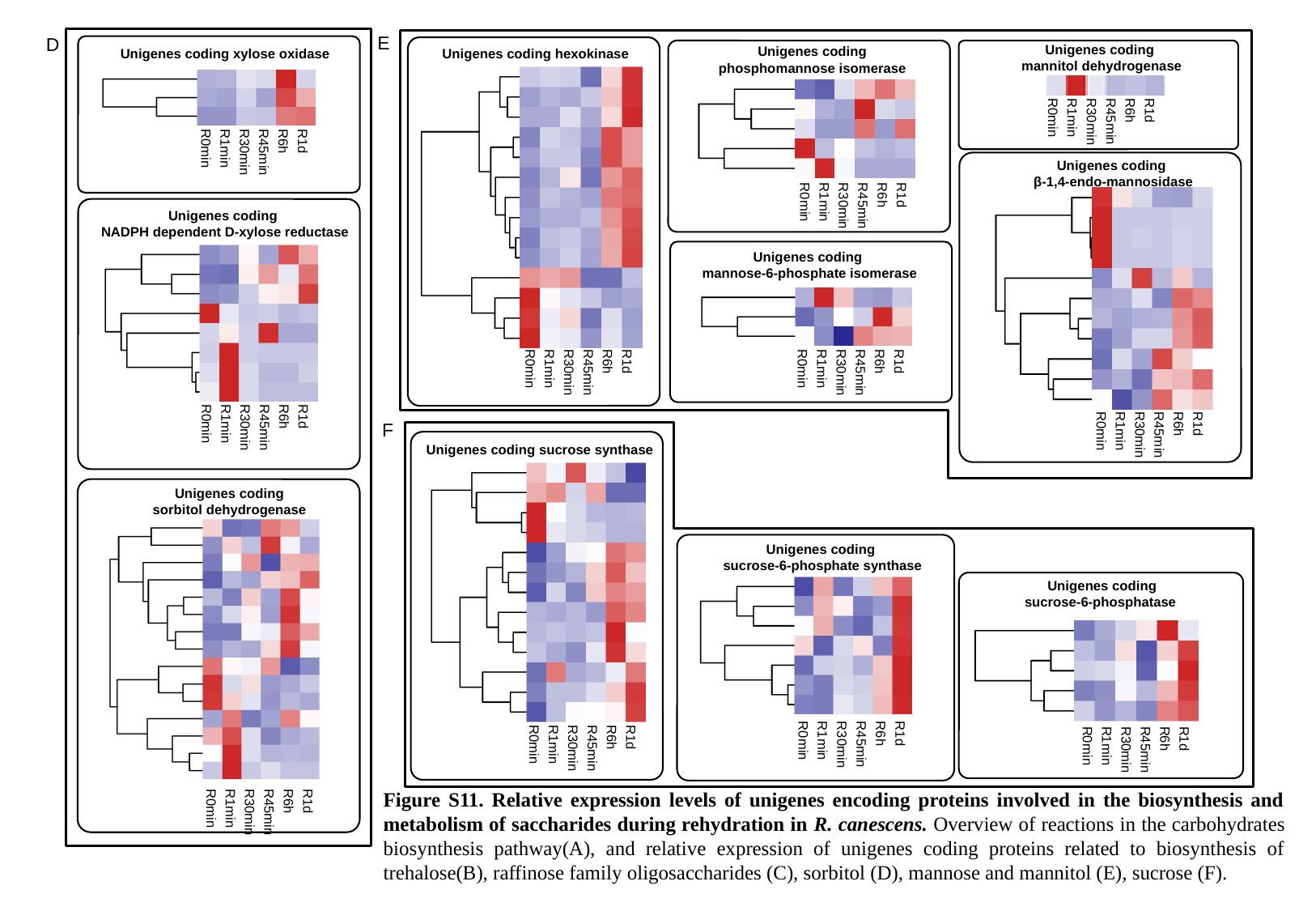

E
Unigenes coding
mannitol dehydrogenase
Unigenes coding
phosphomannose isomerase
Unigenes coding hexokinase
R1d
R6h
R45min
R30min
R1min
R0min
R1d
R6h
R45min
R30min
R1min
R0min
Unigenes coding
β-1,4-endo-mannosidase
Unigenes coding
mannose-6-phosphate isomerase
R1d
R6h
R45min
R30min
R1min
R0min
R1d
R6h
R45min
R30min
R1min
R0min
R1d
R6h
R45min
R30min
R1min
R0min
D
Unigenes coding xylose oxidase
R1d
R6h
R45min
R30min
R1min
R0min
Unigenes coding
NADPH dependent D-xylose reductase
R1d
R6h
R45min
R30min
R1min
R0min
Unigenes coding
sorbitol dehydrogenase
R1d
R6h
R45min
R30min
R1min
R0min
F
Unigenes coding sucrose synthase
Unigenes coding
sucrose-6-phosphate synthase
Unigenes coding
sucrose-6-phosphatase
R1d
R6h
R45min
R30min
R1min
R0min
R1d
R6h
R45min
R30min
R1min
R0min
R1d
R6h
R45min
R30min
R1min
R0min
Figure S11. Relative expression levels of unigenes encoding proteins involved in the biosynthesis and metabolism of saccharides during rehydration in R. canescens. Overview of reactions in the carbohydrates biosynthesis pathway(A), and relative expression of unigenes coding proteins related to biosynthesis of trehalose(B), raffinose family oligosaccharides (C), sorbitol (D), mannose and mannitol (E), sucrose (F).
