# Supplementary material for: Proteomic and Transcriptomic Responses of the Desiccation-Tolerant Moss Racomitrium canescens in the Rapid Rehydration Processes
Source: Genes (Basel). 2023 Feb 2;14(2):390. doi: 10.3390/genes14020390 (PMC9956249; doi:10.3390/genes14020390)
Supplement: Supplementary file 1 [file genes-14-00390-s001.zip › figure S12.pptx]

## Slide 1
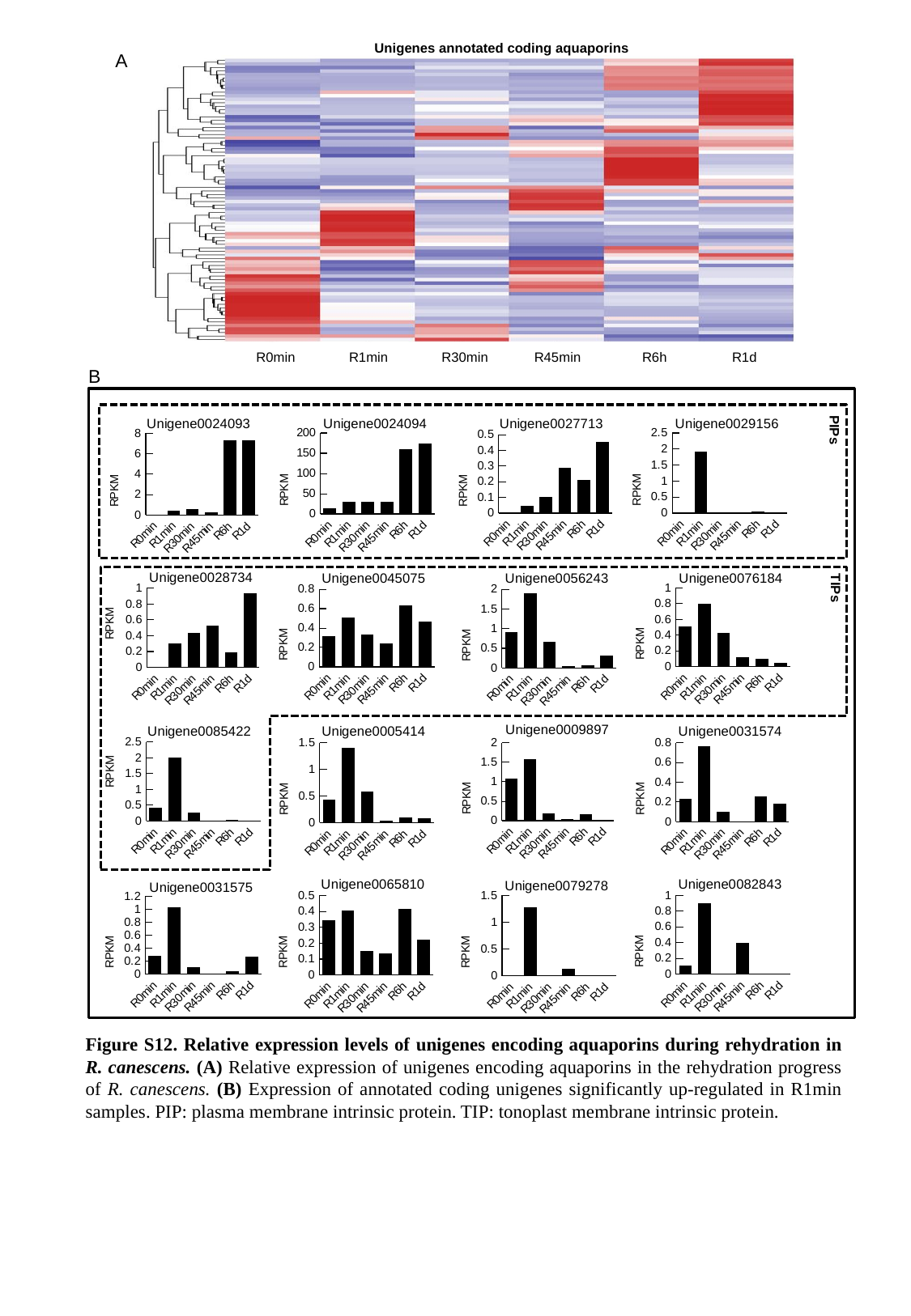

Unigenes annotated coding aquaporins
A
R0min R1min R30min R45min R6h R1d
B
### Chart: Unigene0027713
| Category | Unigene0027713 |
|---|---|
| R0min | 0.0 |
| R1min | 0.045233333 |
| R30min | 0.100766667 |
| R45min | 0.290566667 |
| R6h | 0.2134 |
| R1d | 0.455933333 |
### Chart: Unigene0029156
| Category | Unigene0029156 |
|---|---|
| R0min | 0.0 |
| R1min | 1.9255000000000002 |
| R30min | 0.0258 |
| R45min | 0.016999999999999998 |
| R6h | 0.0405 |
| R1d | 0.014066666666666667 |
### Chart: Unigene0024094
| Category | Unigene0024094 |
|---|---|
| R0min | 13.87443333 |
| R1min | 30.0294 |
| R30min | 30.83786667 |
| R45min | 29.67876667 |
| R6h | 160.2789 |
| R1d | 174.6489 |
### Chart: Unigene0024093
| Category | Unigene0024093 |
|---|---|
| R0min | 0.0234 |
| R1min | 0.4159 |
| R30min | 0.620466667 |
| R45min | 0.276433333 |
| R6h | 7.287433333 |
| R1d | 7.310533333 |
PIPs
### Chart: Unigene0045075
| Category | Unigene0045075 |
|---|---|
| R0min | 0.3149 |
| R1min | 0.5113666666666666 |
| R30min | 0.33096666666666663 |
| R45min | 0.24430000000000004 |
| R6h | 0.6314 |
| R1d | 0.46849999999999997 |
### Chart: Unigene0056243
| Category | Unigene0056243 |
|---|---|
| R0min | 0.9182666666666667 |
| R1min | 1.8989666666666667 |
| R30min | 0.6730333333333335 |
| R45min | 0.04346666666666666 |
| R6h | 0.06813333333333334 |
| R1d | 0.3239 |
### Chart: Unigene0076184
| Category | Unigene0076184 |
|---|---|
| R0min | 0.5077 |
| R1min | 0.8027 |
| R30min | 0.427466666666667 |
| R45min | 0.115766666666667 |
| R6h | 0.103166666666667 |
| R1d | 0.0449333333333333 |
### Chart: Unigene0028734
| Category | Unigene0028734 |
|---|---|
| R0min | 0.0 |
| R1min | 0.3039 |
| R30min | 0.44 |
| R45min | 0.527966667 |
| R6h | 0.192 |
| R1d | 0.934133333 |
TIPs
### Chart: Unigene0005414
| Category | Unigene0005414 |
|---|---|
| R0min | 0.431433333333333 |
| R1min | 1.4102 |
| R30min | 0.5773 |
| R45min | 0.0364 |
| R6h | 0.0906666666666667 |
| R1d | 0.0754 |
### Chart: Unigene0009897
| Category | Unigene0009897 |
|---|---|
| R0min | 1.07896666666667 |
| R1min | 1.5743 |
| R30min | 0.1895 |
| R45min | 0.0491333333333333 |
| R6h | 0.172 |
| R1d | 0.0 |
### Chart: Unigene0031574
| Category | Unigene0031574 |
|---|---|
| R0min | 0.2331 |
| R1min | 0.761 |
| R30min | 0.0971 |
| R45min | 0.0 |
| R6h | 0.2579 |
| R1d | 0.179533333333333 |
### Chart: Unigene0085422
| Category | Unigene0085422 |
|---|---|
| R0min | 0.418766666666667 |
| R1min | 2.01026666666667 |
| R30min | 0.2708 |
| R45min | 0.0 |
| R6h | 0.0464666666666667 |
| R1d | 0.0193 |
### Chart: Unigene0065810
| Category | Unigene0065810 |
|---|---|
| R0min | 0.3432 |
| R1min | 0.40276666666666666 |
| R30min | 0.15306666666666666 |
| R45min | 0.13396666666666668 |
| R6h | 0.41683333333333333 |
| R1d | 0.2197666666666667 |
### Chart: Unigene0079278
| Category | Unigene0079278 |
|---|---|
| R0min | 0.0 |
| R1min | 1.2813666666666668 |
| R30min | 0.0 |
| R45min | 0.13546666666666665 |
| R6h | 0.0 |
| R1d | 0.0 |
### Chart: Unigene0082843
| Category | Unigene0082843 |
|---|---|
| R0min | 0.11046666666666666 |
| R1min | 0.9041333333333332 |
| R30min | 0.0 |
| R45min | 0.4005666666666667 |
| R6h | 0.0 |
| R1d | 0.0 |
### Chart: Unigene0031575
| Category | Unigene0031575 |
|---|---|
| R0min | 0.284133333333333 |
| R1min | 1.03153333333333 |
| R30min | 0.105666666666667 |
| R45min | 0.0 |
| R6h | 0.048 |
| R1d | 0.273966666666667 |Figure S12. Relative expression levels of unigenes encoding aquaporins during rehydration in R. canescens. (A) Relative expression of unigenes encoding aquaporins in the rehydration progress of R. canescens. (B) Expression of annotated coding unigenes significantly up-regulated in R1min samples. PIP: plasma membrane intrinsic protein. TIP: tonoplast membrane intrinsic protein.
