# Supplementary material for: Proteomic and Transcriptomic Responses of the Desiccation-Tolerant Moss Racomitrium canescens in the Rapid Rehydration Processes
Source: Genes (Basel). 2023 Feb 2;14(2):390. doi: 10.3390/genes14020390 (PMC9956249; doi:10.3390/genes14020390)
Supplement: Supplementary file 1 [file genes-14-00390-s001.zip › figure S13.pptx]

## Slide 1
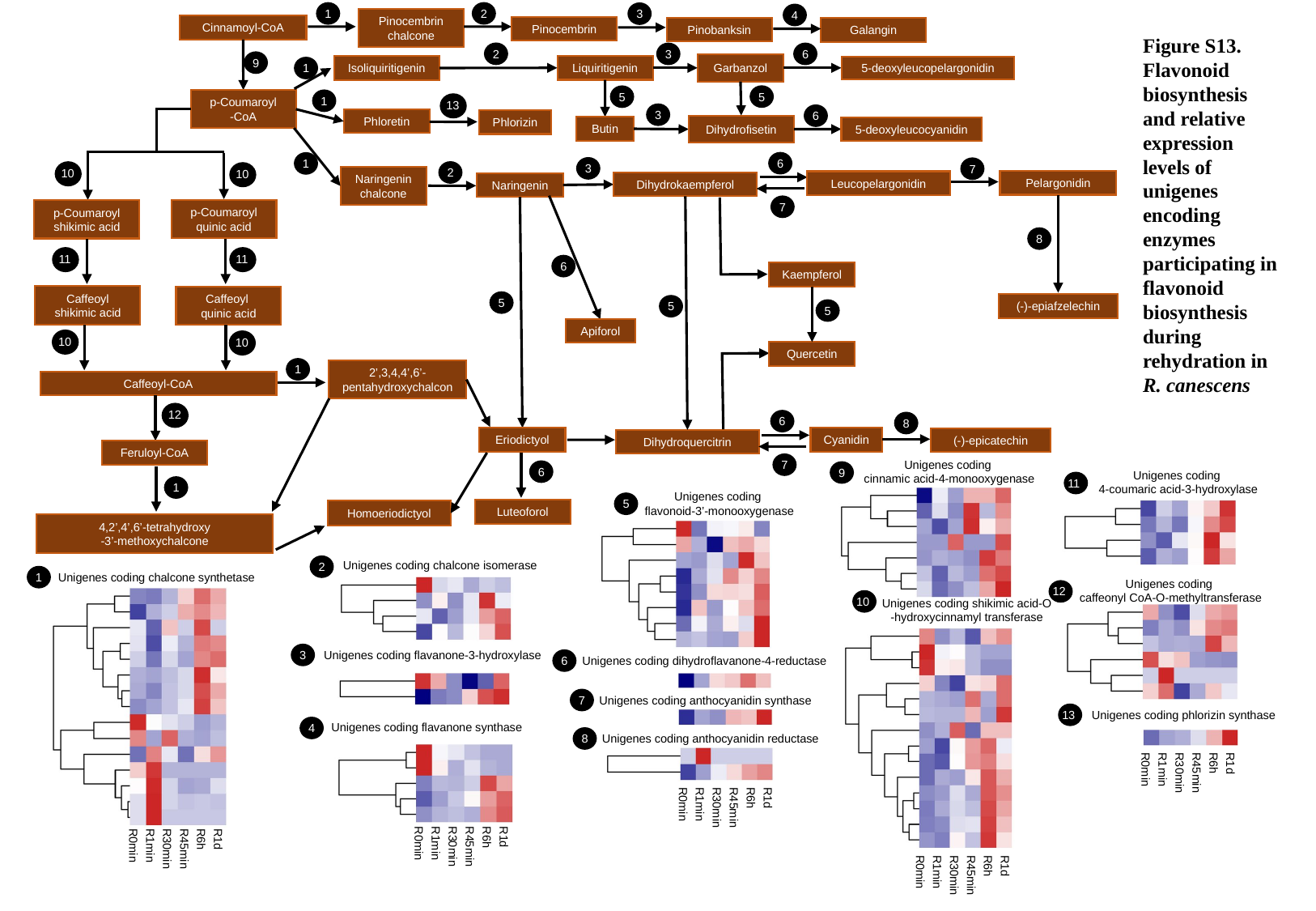

2
1
3
4
Pinocembrin
chalcone
Cinnamoyl-CoA
Pinocembrin
Pinobanksin
Galangin
2
6
3
9
Garbanzol
Liquiritigenin
Isoliquiritigenin
5-deoxyleucopelargonidin
1
5
5
p-Coumaroyl
-CoA
1
13
3
6
Phloretin
Phlorizin
Dihydrofisetin
Butin
5-deoxyleucocyanidin
6
1
3
7
10
10
2
Naringenin chalcone
Pelargonidin
Leucopelargonidin
Dihydrokaempferol
Naringenin
7
p-Coumaroyl
quinic acid
p-Coumaroyl
shikimic acid
8
11
11
6
Kaempferol
Caffeoyl
shikimic acid
Caffeoyl
quinic acid
5
(-)-epiafzelechin
5
5
Apiforol
10
10
Quercetin
1
2’,3,4,4’,6’-
pentahydroxychalcon
Caffeoyl-CoA
12
6
8
Cyanidin
Eriodictyol
(-)-epicatechin
Dihydroquercitrin
Feruloyl-CoA
7
6
1
Luteoforol
Homoeriodictyol
4,2’,4’,6’-tetrahydroxy
-3’-methoxychalcone
Unigenes coding
cinnamic acid-4-monooxygenase
Unigenes coding
4-coumaric acid-3-hydroxylase
9
11
Unigenes coding
flavonoid-3’-monooxygenase
5
Unigenes coding chalcone isomerase
2
Unigenes coding chalcone synthetase
1
Unigenes coding
caffeonyl CoA-O-methyltransferase
12
10
Unigenes coding shikimic acid-O
-hydroxycinnamyl transferase
Unigenes coding flavanone-3-hydroxylase
3
Unigenes coding dihydroflavanone-4-reductase
6
Unigenes coding anthocyanidin synthase
7
Unigenes coding phlorizin synthase
13
Unigenes coding flavanone synthase
R1d
R6h
R45min
R30min
R1min
R0min
4
Unigenes coding anthocyanidin reductase
8
R1d
R6h
R45min
R30min
R1min
R0min
R1d
R6h
R45min
R30min
R1min
R0min
R1d
R6h
R45min
R30min
R1min
R0min
R1d
R6h
R45min
R30min
R1min
R0min
Figure S13. Flavonoid biosynthesis and relative expression levels of unigenes encoding enzymes participating in flavonoid biosynthesis during rehydration in R. canescens
