# Supplementary material for: Proteomic and Transcriptomic Responses of the Desiccation-Tolerant Moss Racomitrium canescens in the Rapid Rehydration Processes
Source: Genes (Basel). 2023 Feb 2;14(2):390. doi: 10.3390/genes14020390 (PMC9956249; doi:10.3390/genes14020390)
Supplement: Supplementary file 1 [file genes-14-00390-s001.zip › figure S14.pptx]

## Slide 1
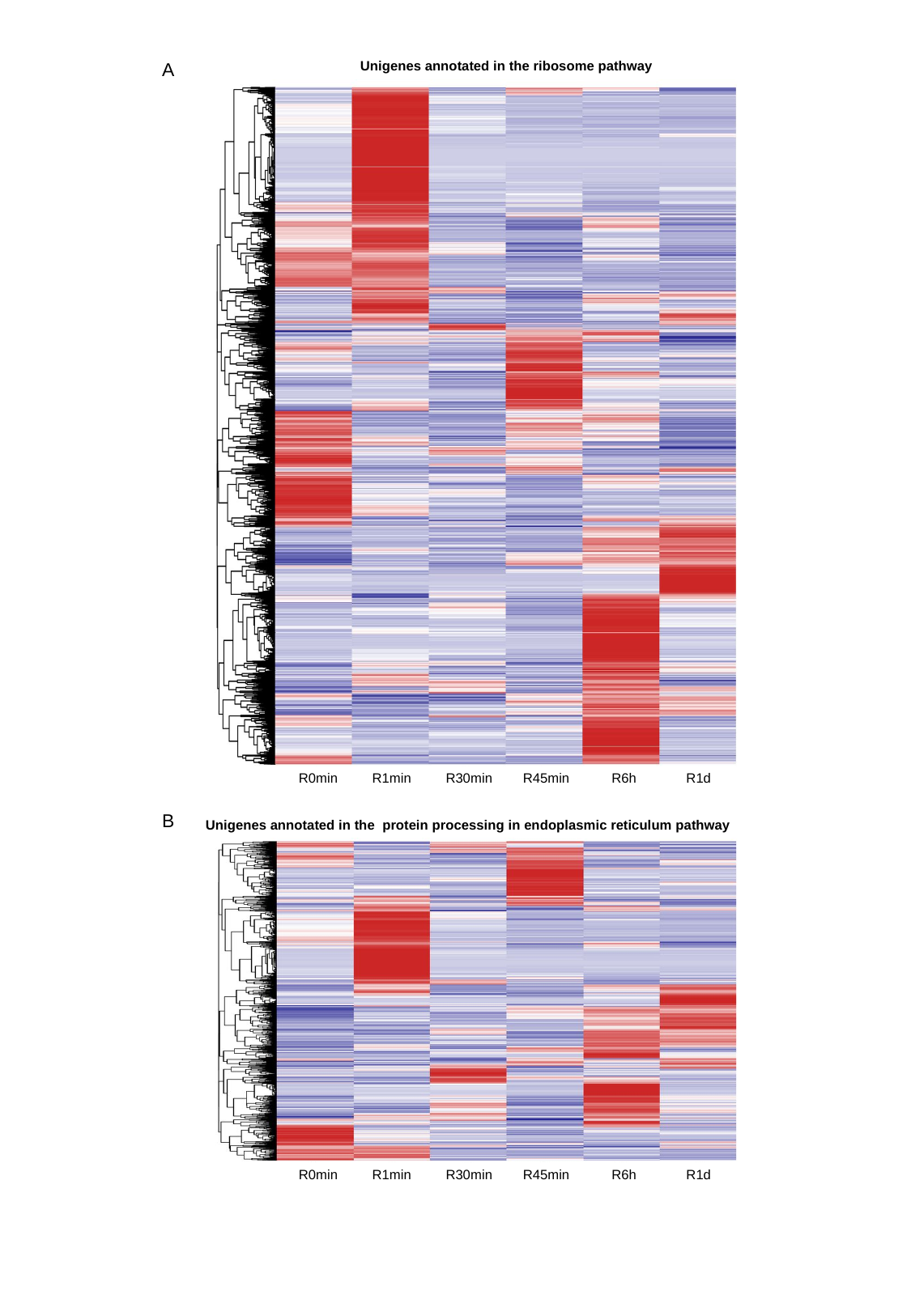

A
Unigenes annotated in the ribosome pathway
R0min R1min R30min R45min R6h R1d
B
Unigenes annotated in the protein processing in endoplasmic reticulum pathway
R0min R1min R30min R45min R6h R1d

## Slide 2
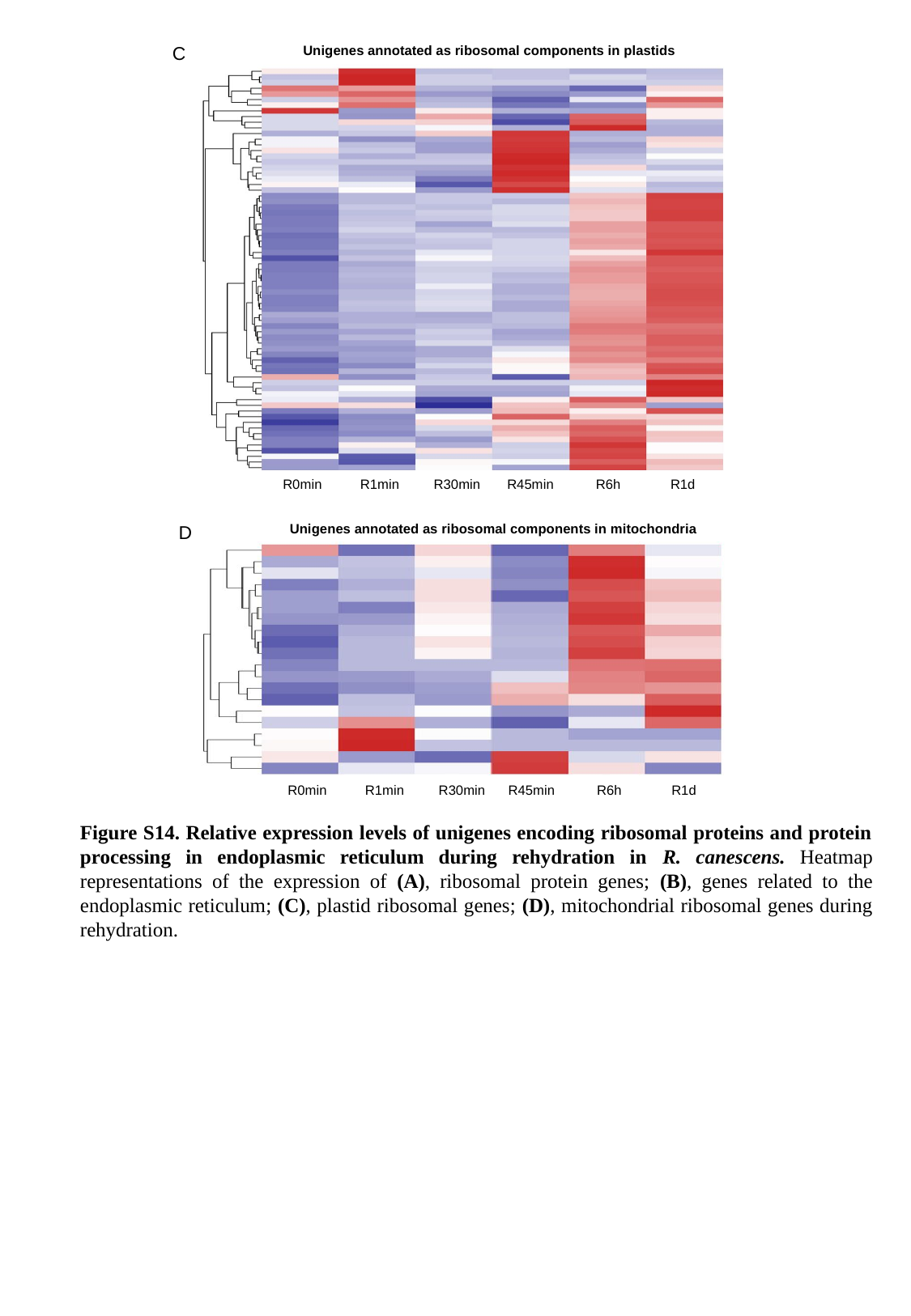

C
Unigenes annotated as ribosomal components in plastids
R0min R1min R30min R45min R6h R1d
Unigenes annotated as ribosomal components in mitochondria
D
R0min R1min R30min R45min R6h R1d
Figure S14. Relative expression levels of unigenes encoding ribosomal proteins and protein processing in endoplasmic reticulum during rehydration in R. canescens. Heatmap representations of the expression of (A), ribosomal protein genes; (B), genes related to the endoplasmic reticulum; (C), plastid ribosomal genes; (D), mitochondrial ribosomal genes during rehydration.
