# Supplementary material for: Proteomic and Transcriptomic Responses of the Desiccation-Tolerant Moss Racomitrium canescens in the Rapid Rehydration Processes
Source: Genes (Basel). 2023 Feb 2;14(2):390. doi: 10.3390/genes14020390 (PMC9956249; doi:10.3390/genes14020390)
Supplement: Supplementary file 1 [file genes-14-00390-s001.zip › figure S15.pptx]

## Slide 1
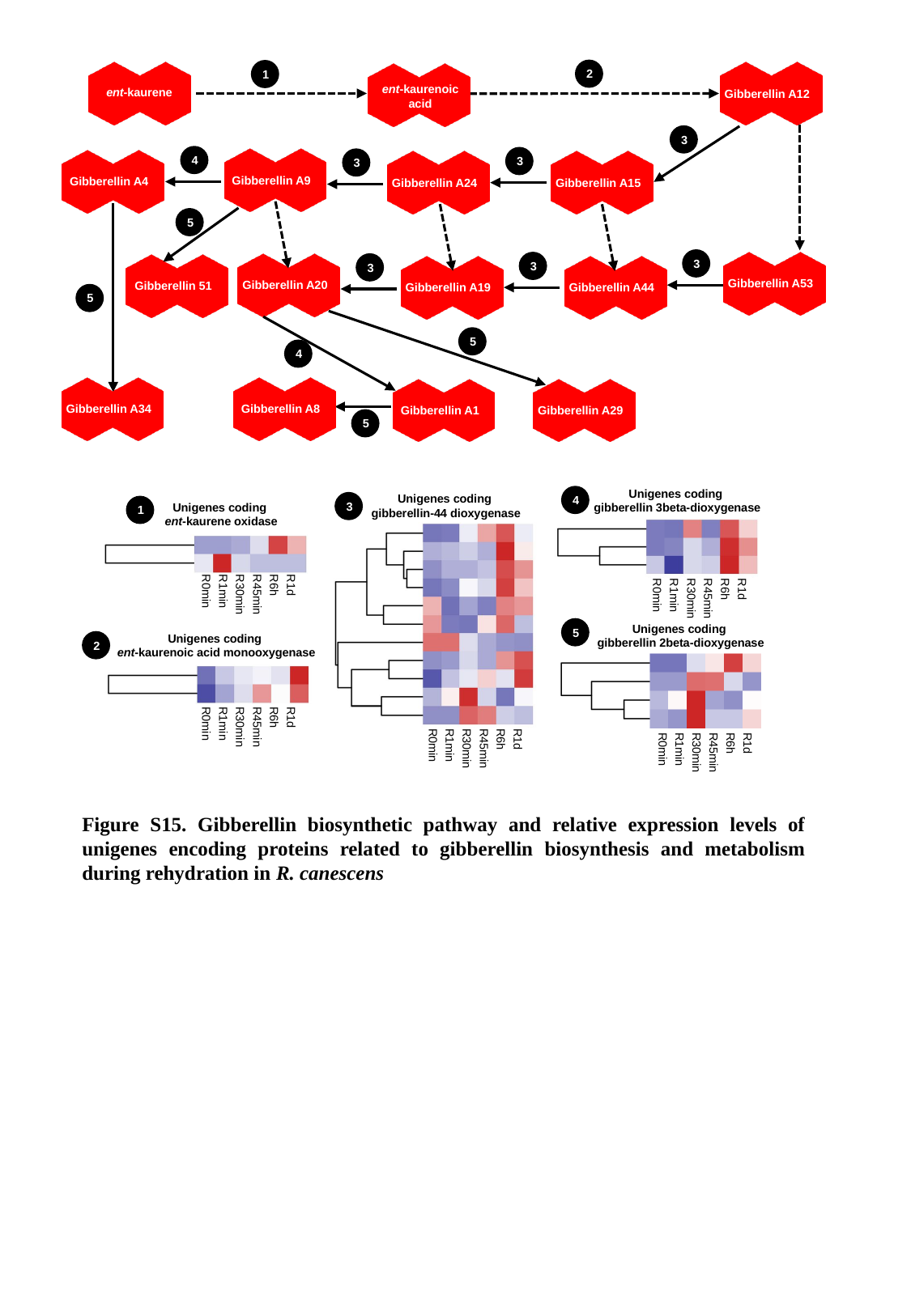

2
1
ent-kaurene
Gibberellin A12
ent-kaurenoic acid
3
4
3
Gibberellin A9
3
Gibberellin A4
Gibberellin A24
Gibberellin A15
5
3
Gibberellin A53
3
Gibberellin A20
3
Gibberellin 51
Gibberellin A19
Gibberellin A44
5
5
4
Gibberellin A34
Gibberellin A8
Gibberellin A29
Gibberellin A1
5
Unigenes coding
gibberellin 3beta-dioxygenase
R1d
R6h
R45min
R30min
R1min
R0min
Unigenes coding
gibberellin-44 dioxygenase
R1d
R6h
R45min
R30min
R1min
R0min
4
3
Unigenes coding
ent-kaurene oxidase
R1d
R6h
R45min
R30min
R1min
R0min
R1d
R6h
R45min
R30min
R1min
R0min
Unigenes coding
ent-kaurenoic acid monooxygenase
1
Unigenes coding
 gibberellin 2beta-dioxygenase
R1d
R6h
R45min
R30min
R1min
R0min
5
2
Figure S15. Gibberellin biosynthetic pathway and relative expression levels of unigenes encoding proteins related to gibberellin biosynthesis and metabolism during rehydration in R. canescens
