# Supplementary material for: Proteomic and Transcriptomic Responses of the Desiccation-Tolerant Moss Racomitrium canescens in the Rapid Rehydration Processes
Source: Genes (Basel). 2023 Feb 2;14(2):390. doi: 10.3390/genes14020390 (PMC9956249; doi:10.3390/genes14020390)
Supplement: Supplementary file 1 [file genes-14-00390-s001.zip › figure S2.pptx]

## Slide 1
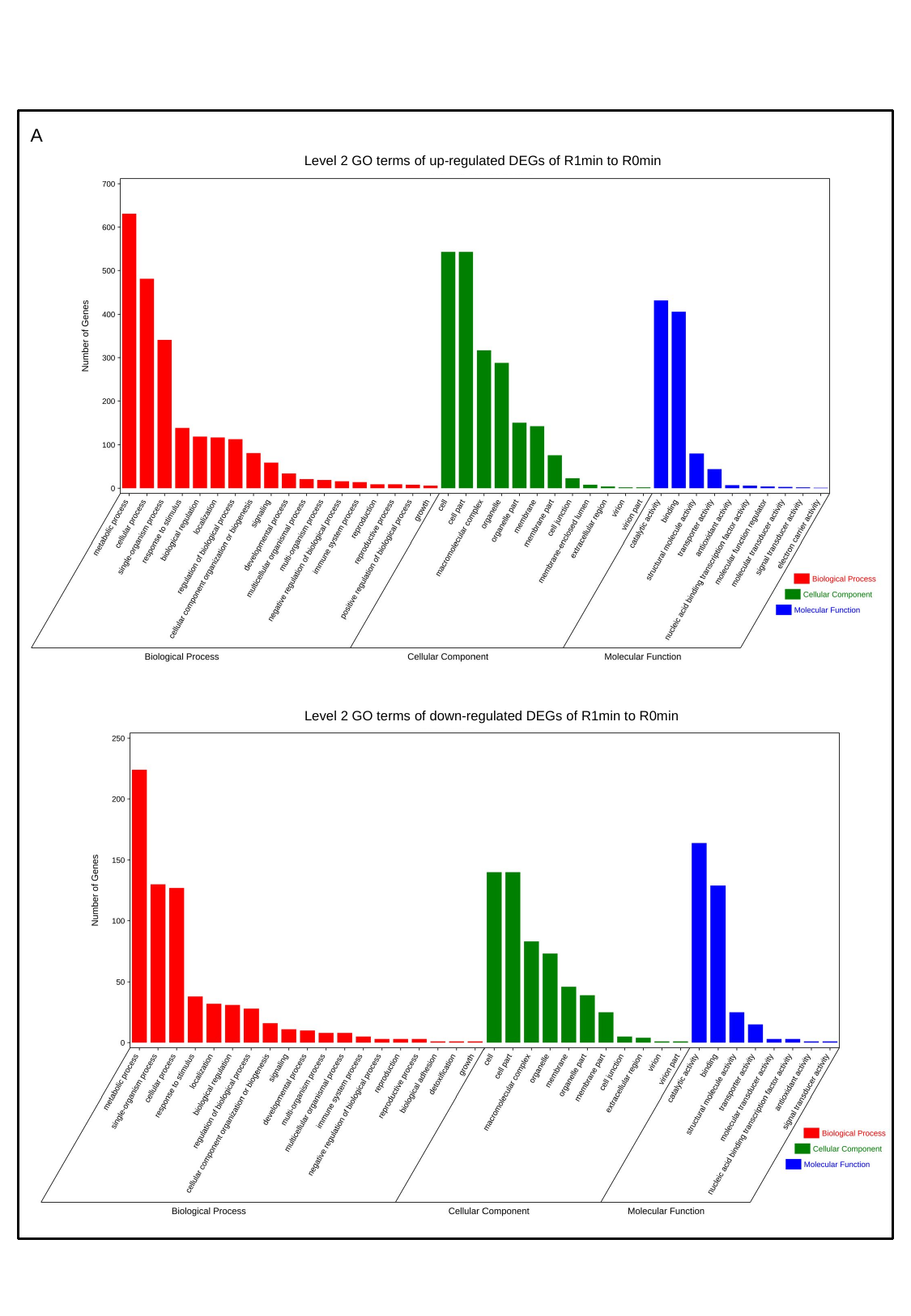

A
Level 2 GO terms of up-regulated DEGs of R1min to R0min
Level 2 GO terms of down-regulated DEGs of R1min to R0min

## Slide 2
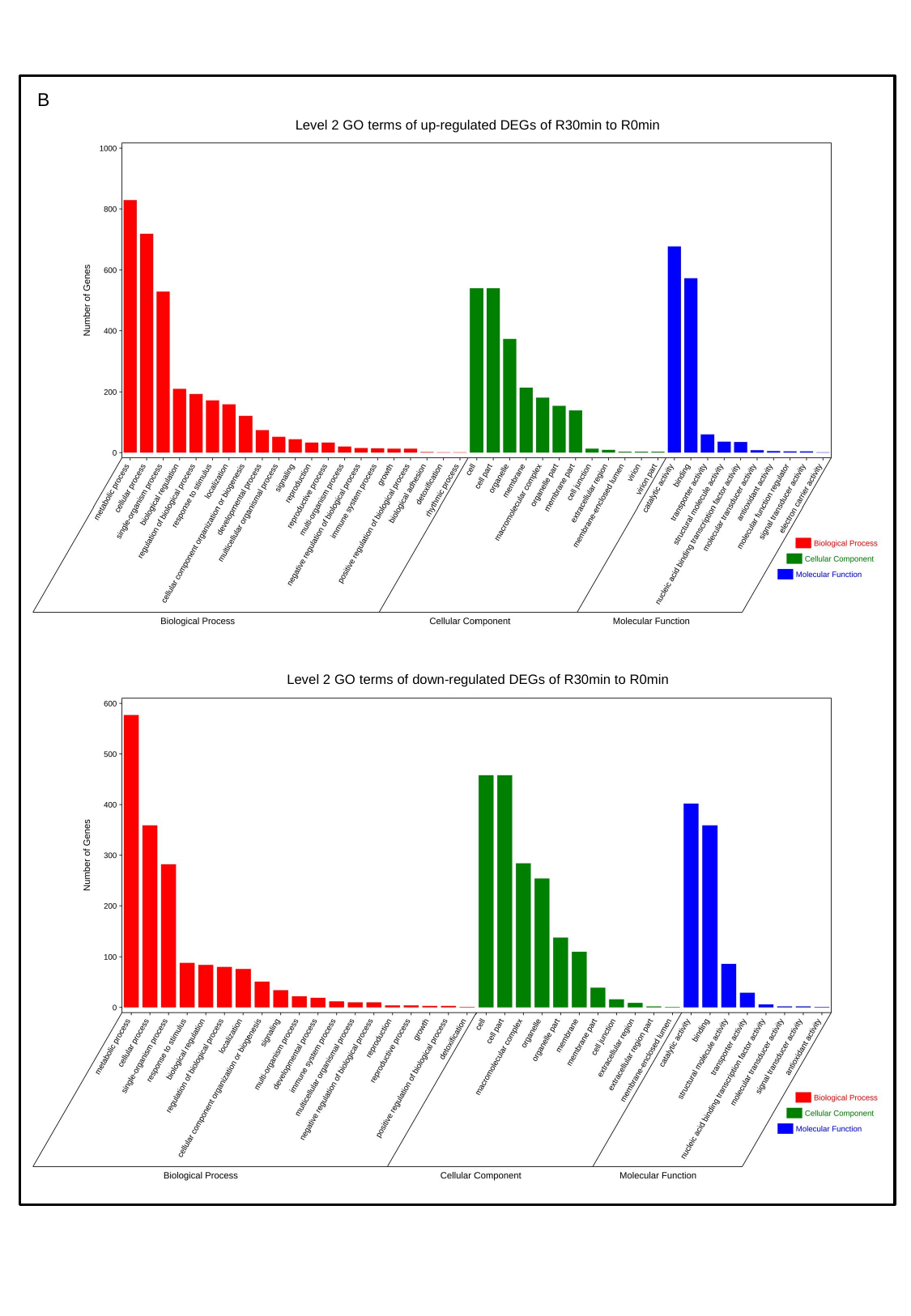

B
Level 2 GO terms of up-regulated DEGs of R30min to R0min
Level 2 GO terms of down-regulated DEGs of R30min to R0min

## Slide 3
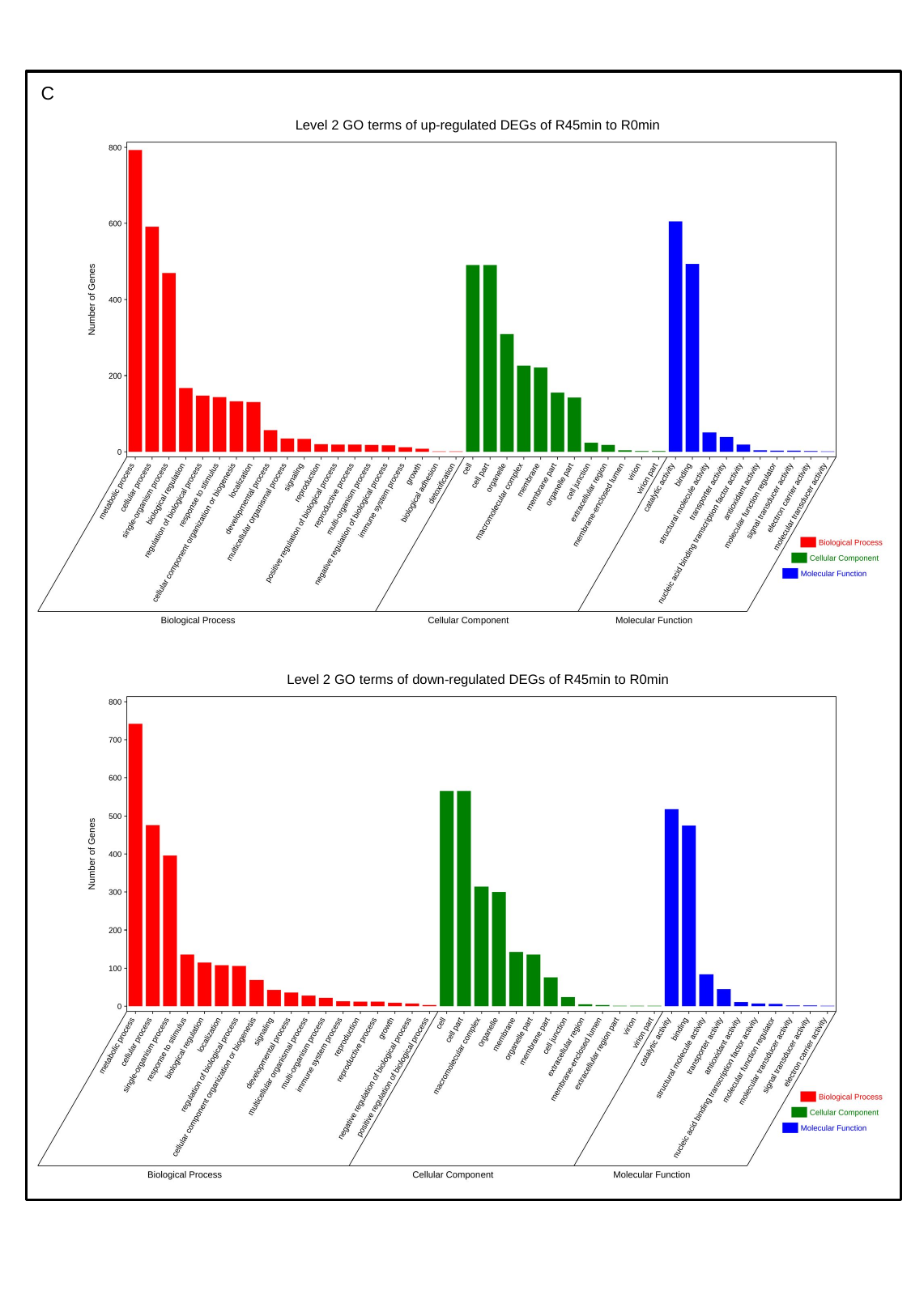

C
Level 2 GO terms of up-regulated DEGs of R45min to R0min
Level 2 GO terms of down-regulated DEGs of R45min to R0min

## Slide 4
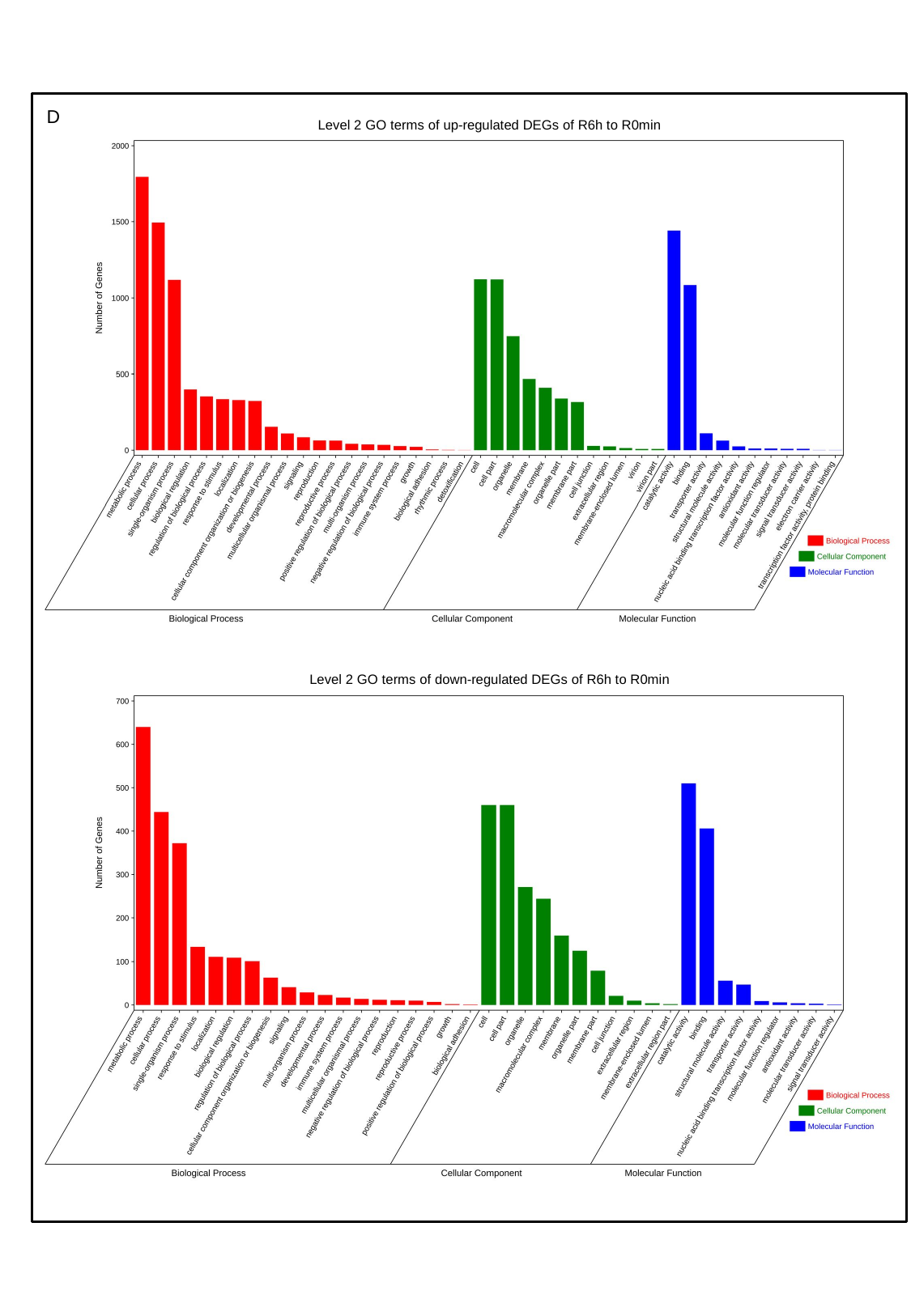

D
Level 2 GO terms of up-regulated DEGs of R6h to R0min
Level 2 GO terms of down-regulated DEGs of R6h to R0min

## Slide 5
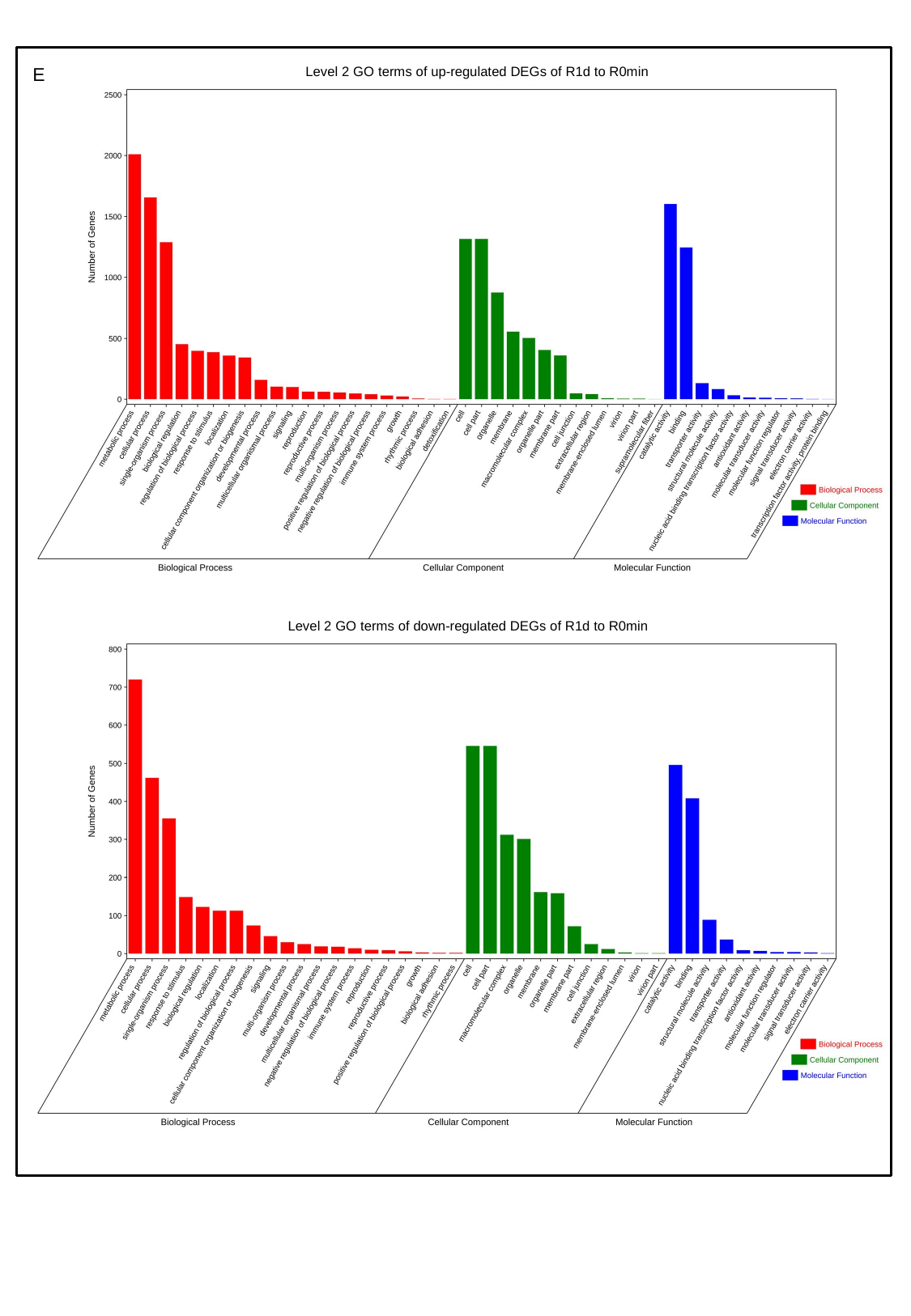

E
Level 2 GO terms of up-regulated DEGs of R1d to R0min
Level 2 GO terms of down-regulated DEGs of R1d to R0min

## Slide 6
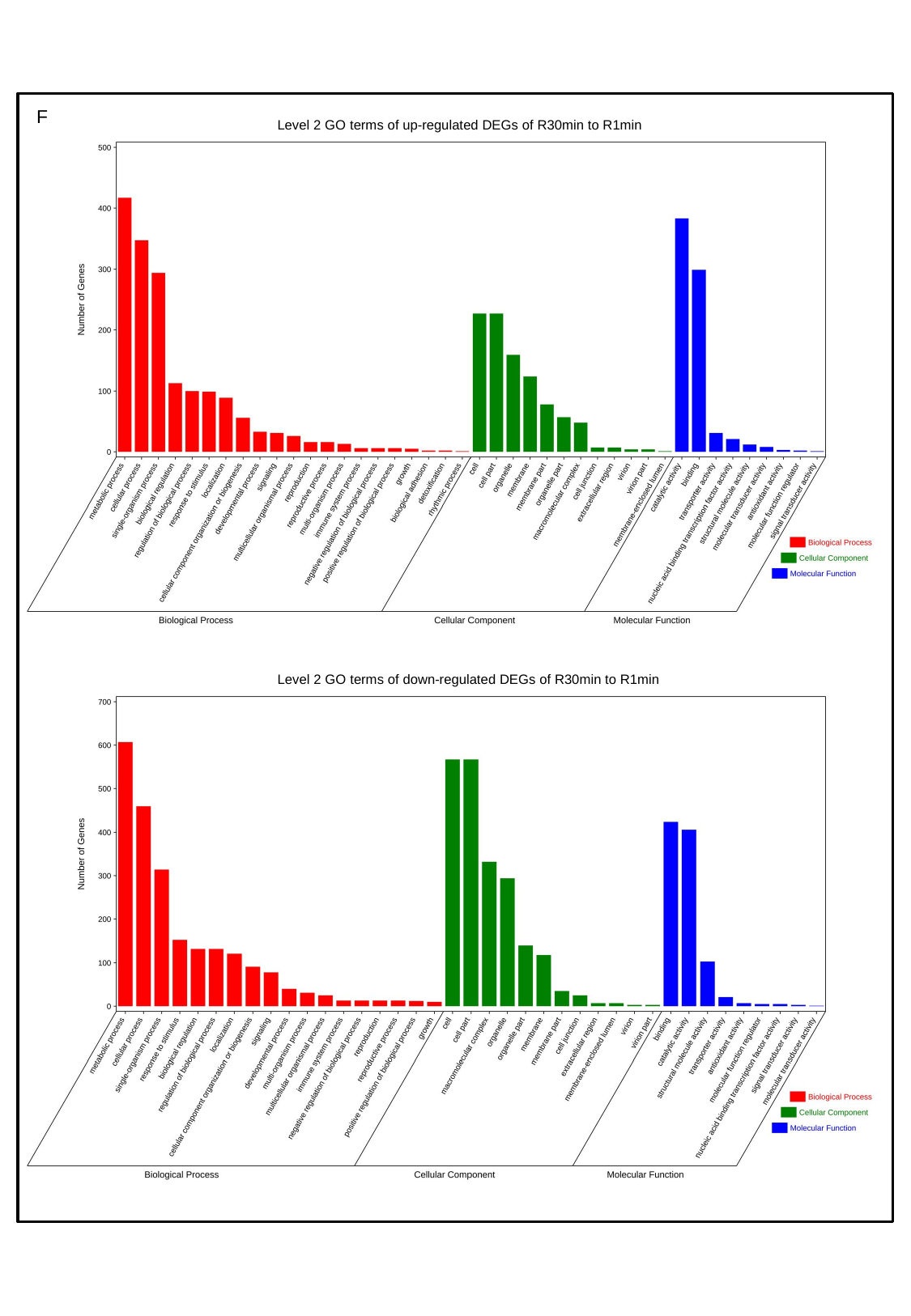

F
Level 2 GO terms of up-regulated DEGs of R30min to R1min
Level 2 GO terms of down-regulated DEGs of R30min to R1min

## Slide 7
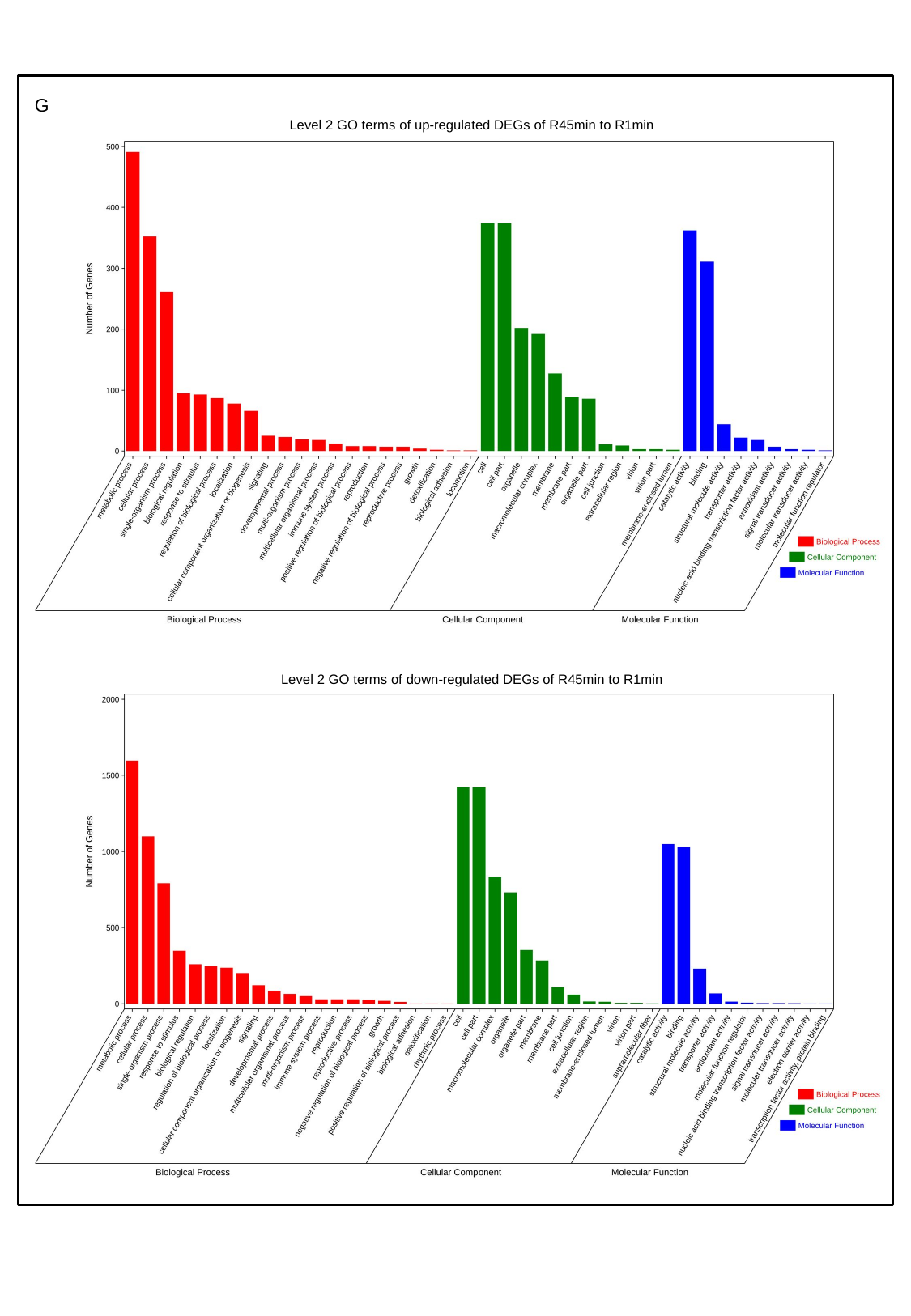

G
Level 2 GO terms of up-regulated DEGs of R45min to R1min
Level 2 GO terms of down-regulated DEGs of R45min to R1min

## Slide 8
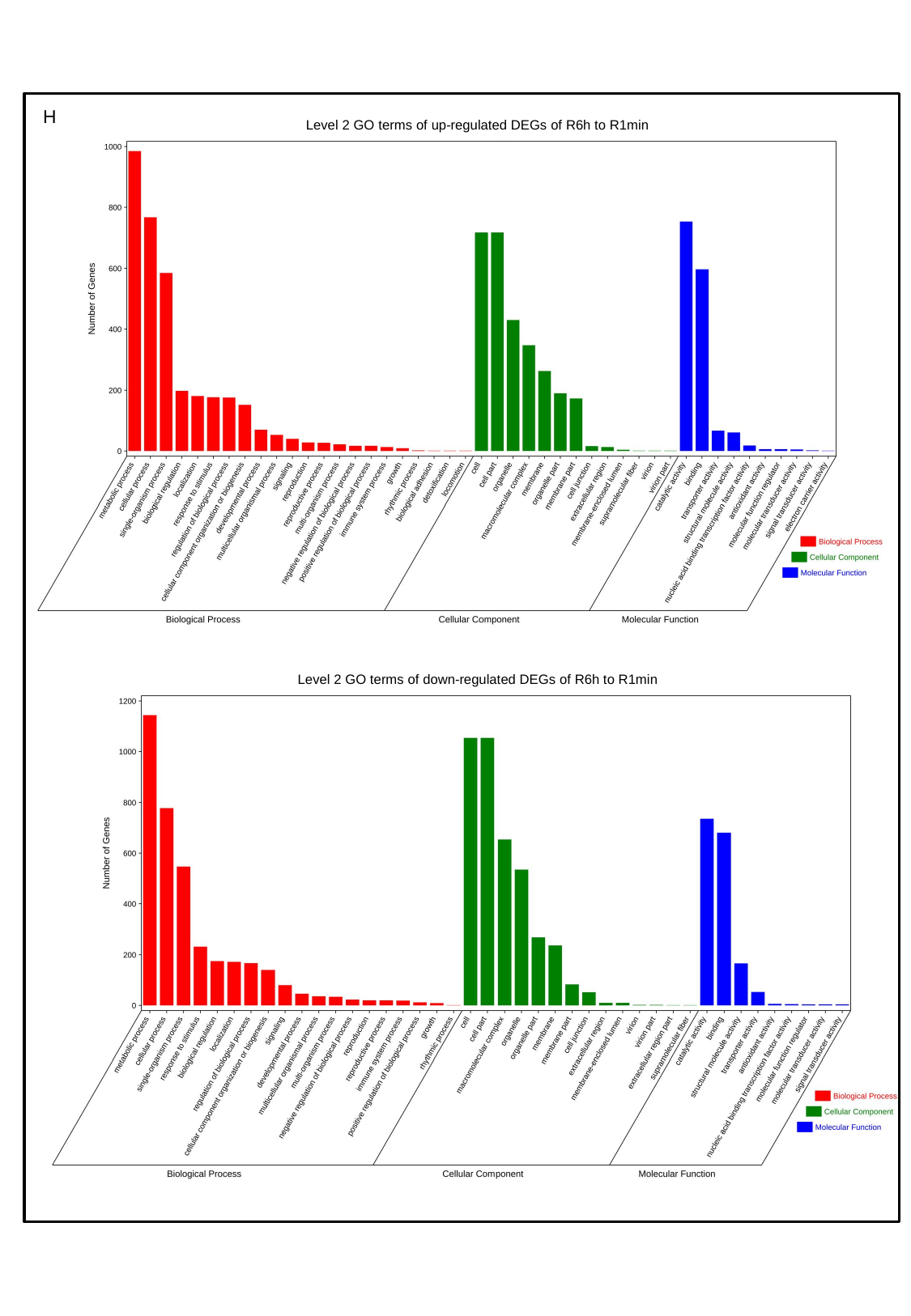

H
Level 2 GO terms of up-regulated DEGs of R6h to R1min
Level 2 GO terms of down-regulated DEGs of R6h to R1min

## Slide 9
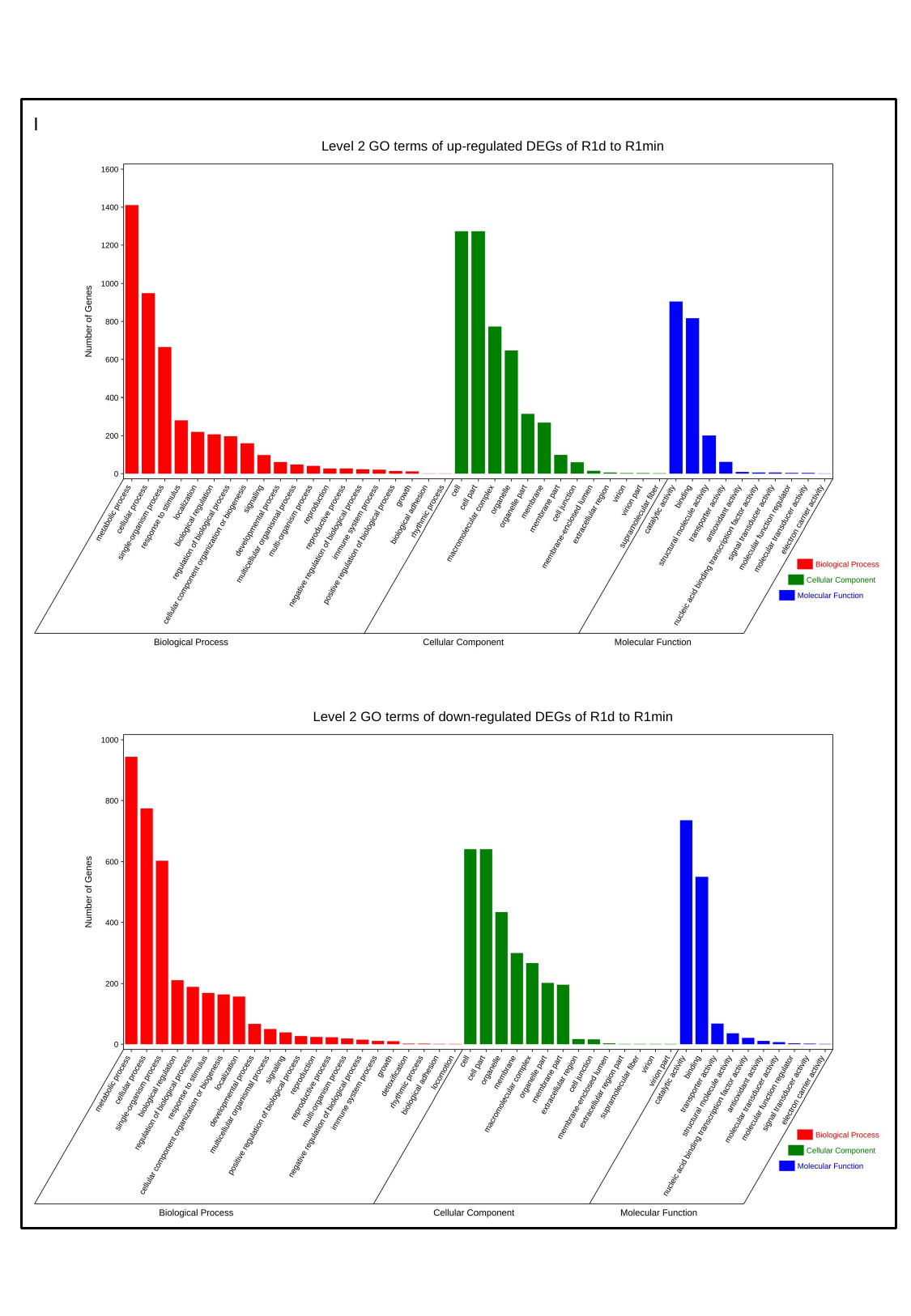

I
Level 2 GO terms of up-regulated DEGs of R1d to R1min
Level 2 GO terms of down-regulated DEGs of R1d to R1min

## Slide 10
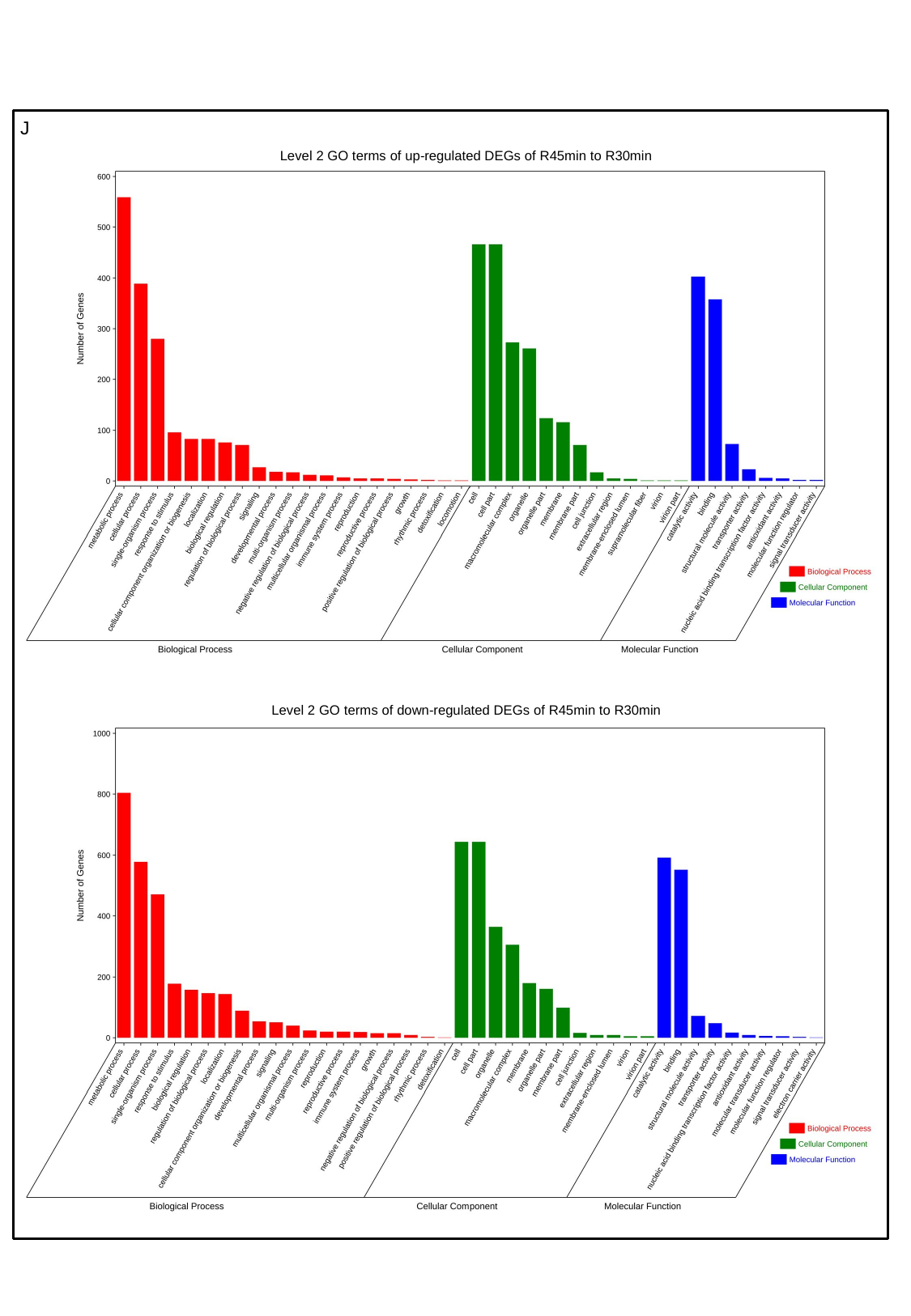

J
Level 2 GO terms of up-regulated DEGs of R45min to R30min
Level 2 GO terms of down-regulated DEGs of R45min to R30min

## Slide 11
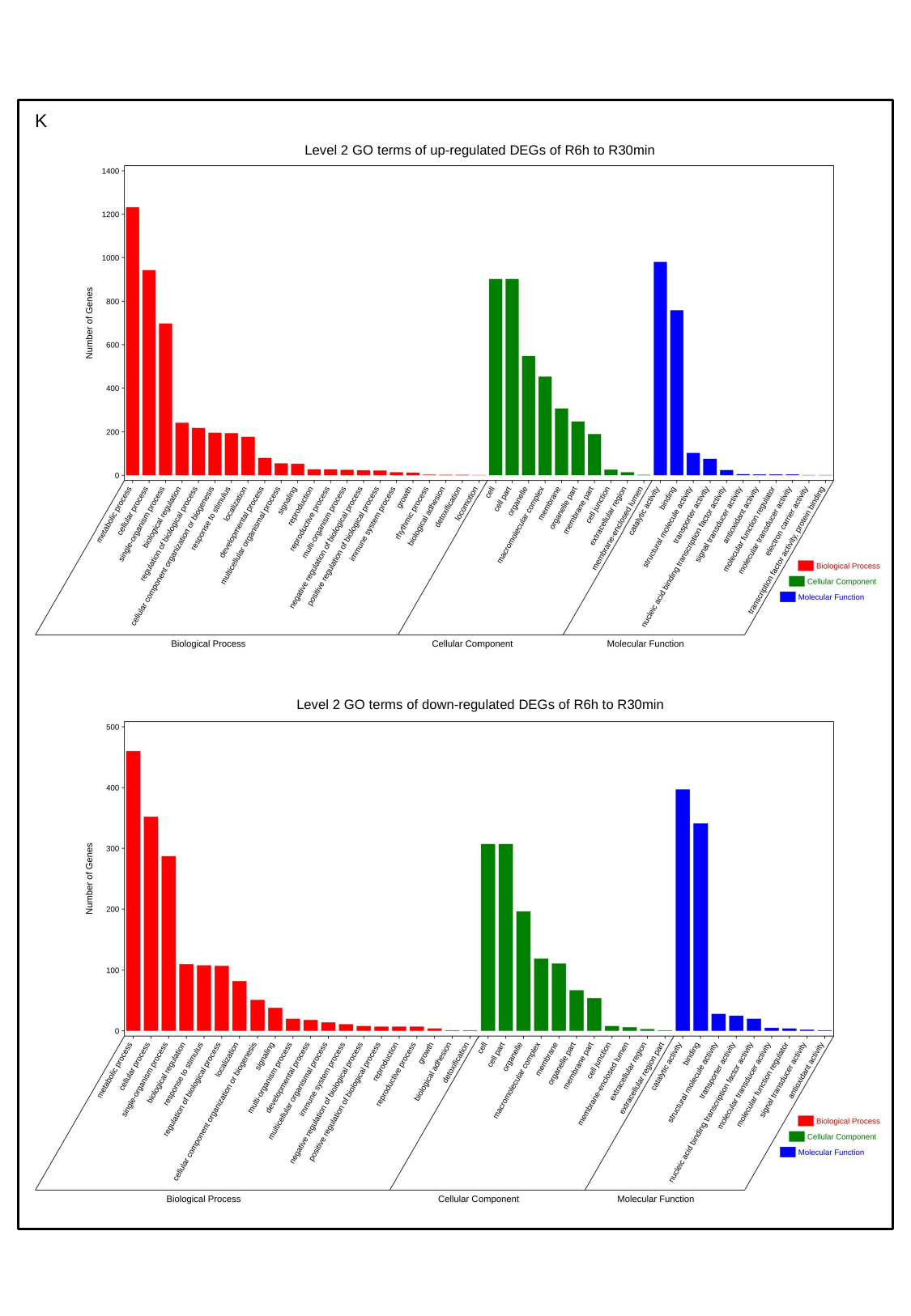

K
Level 2 GO terms of up-regulated DEGs of R6h to R30min
Level 2 GO terms of down-regulated DEGs of R6h to R30min

## Slide 12
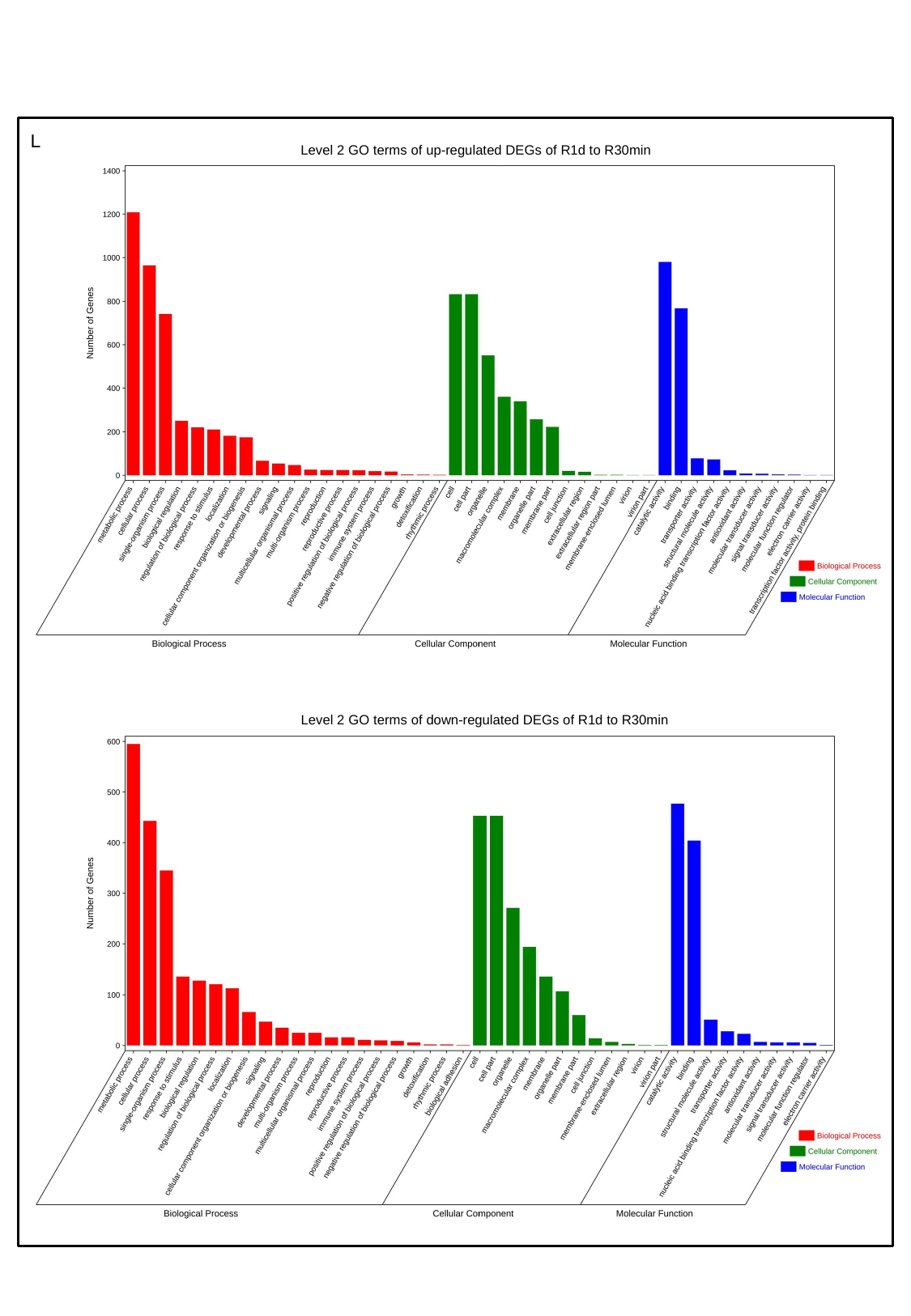

L
Level 2 GO terms of up-regulated DEGs of R1d to R30min
Level 2 GO terms of down-regulated DEGs of R1d to R30min

## Slide 13
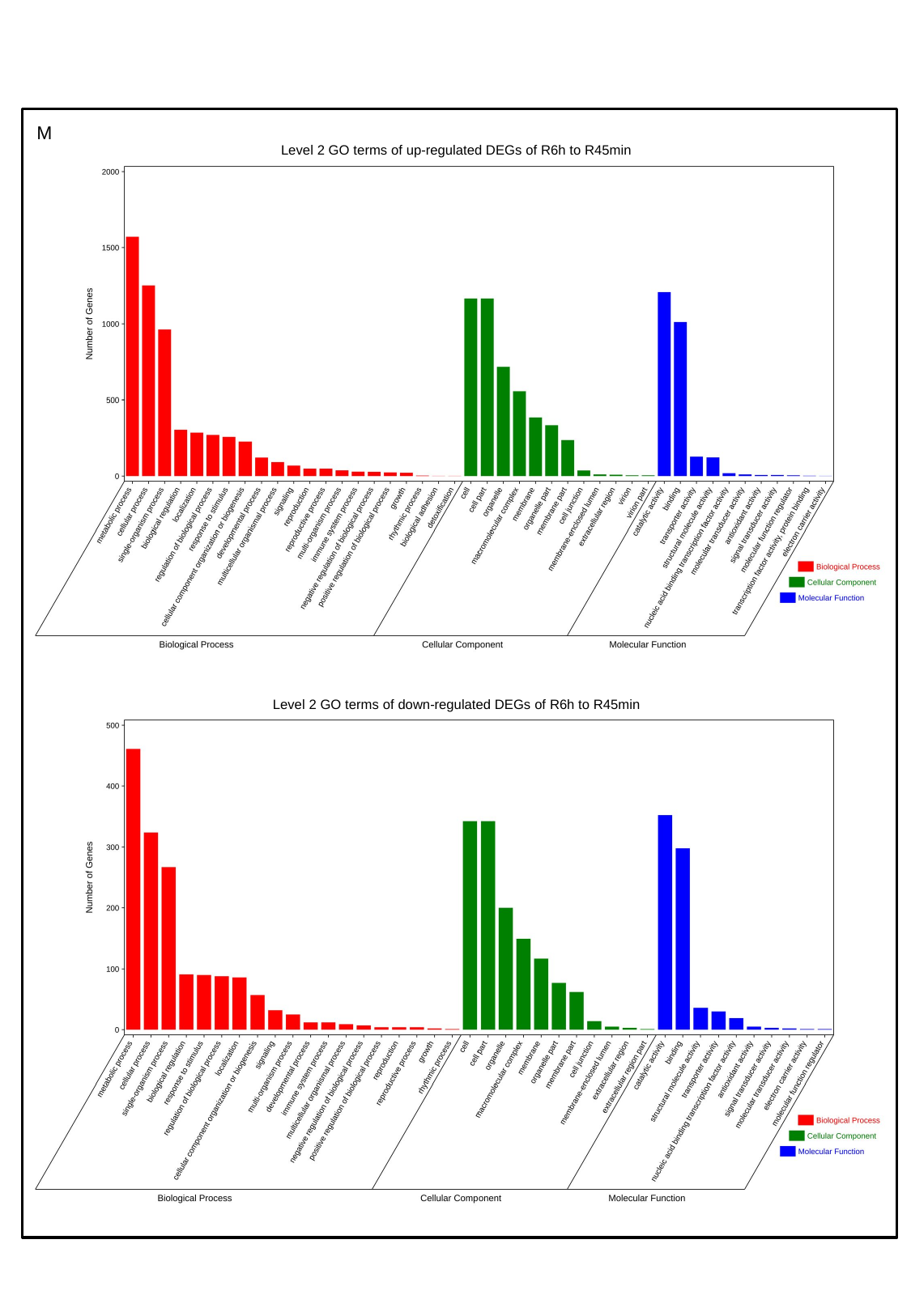

M
Level 2 GO terms of up-regulated DEGs of R6h to R45min
Level 2 GO terms of down-regulated DEGs of R6h to R45min

## Slide 14
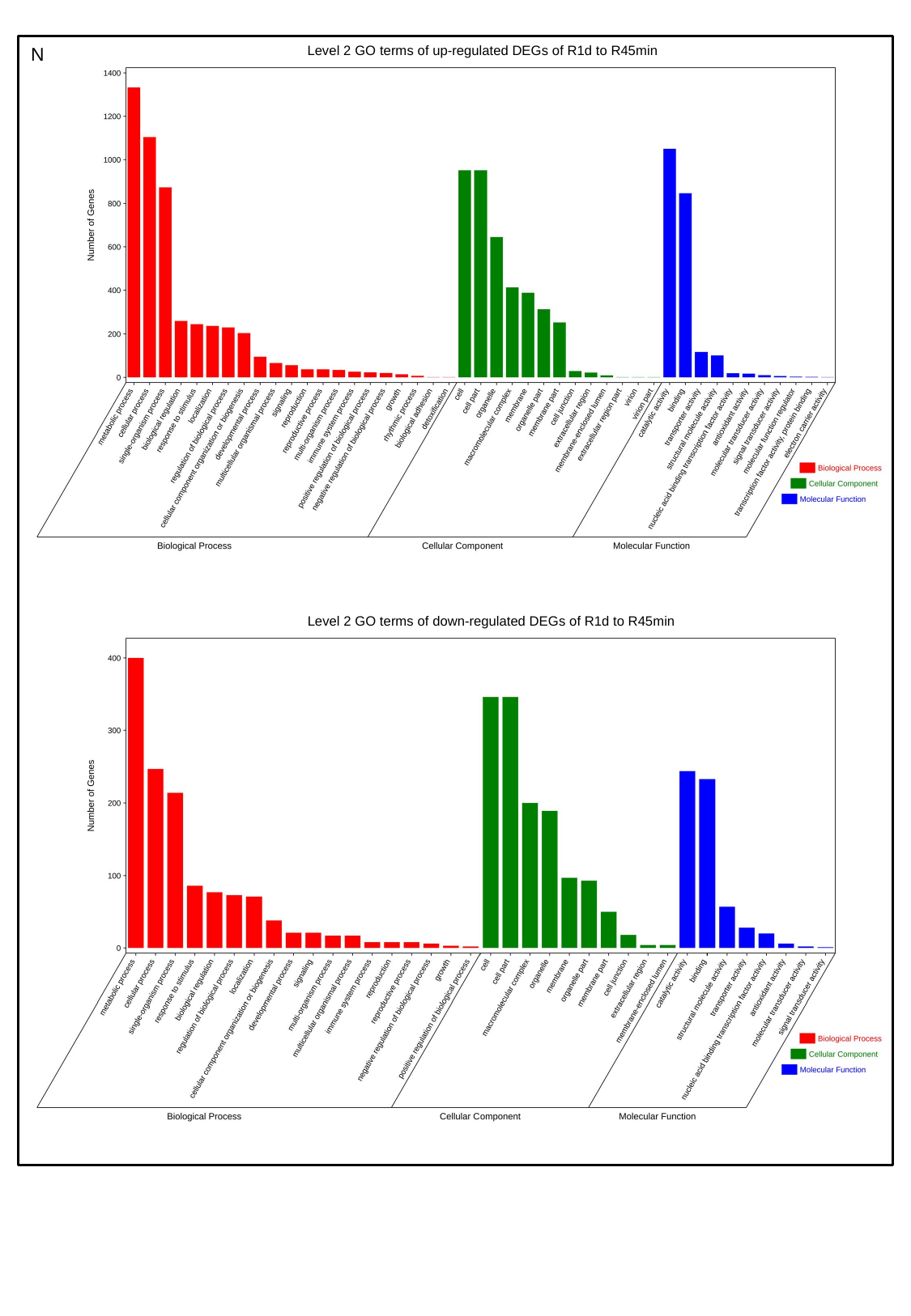

N
Level 2 GO terms of up-regulated DEGs of R1d to R45min
Level 2 GO terms of down-regulated DEGs of R1d to R45min

## Slide 15
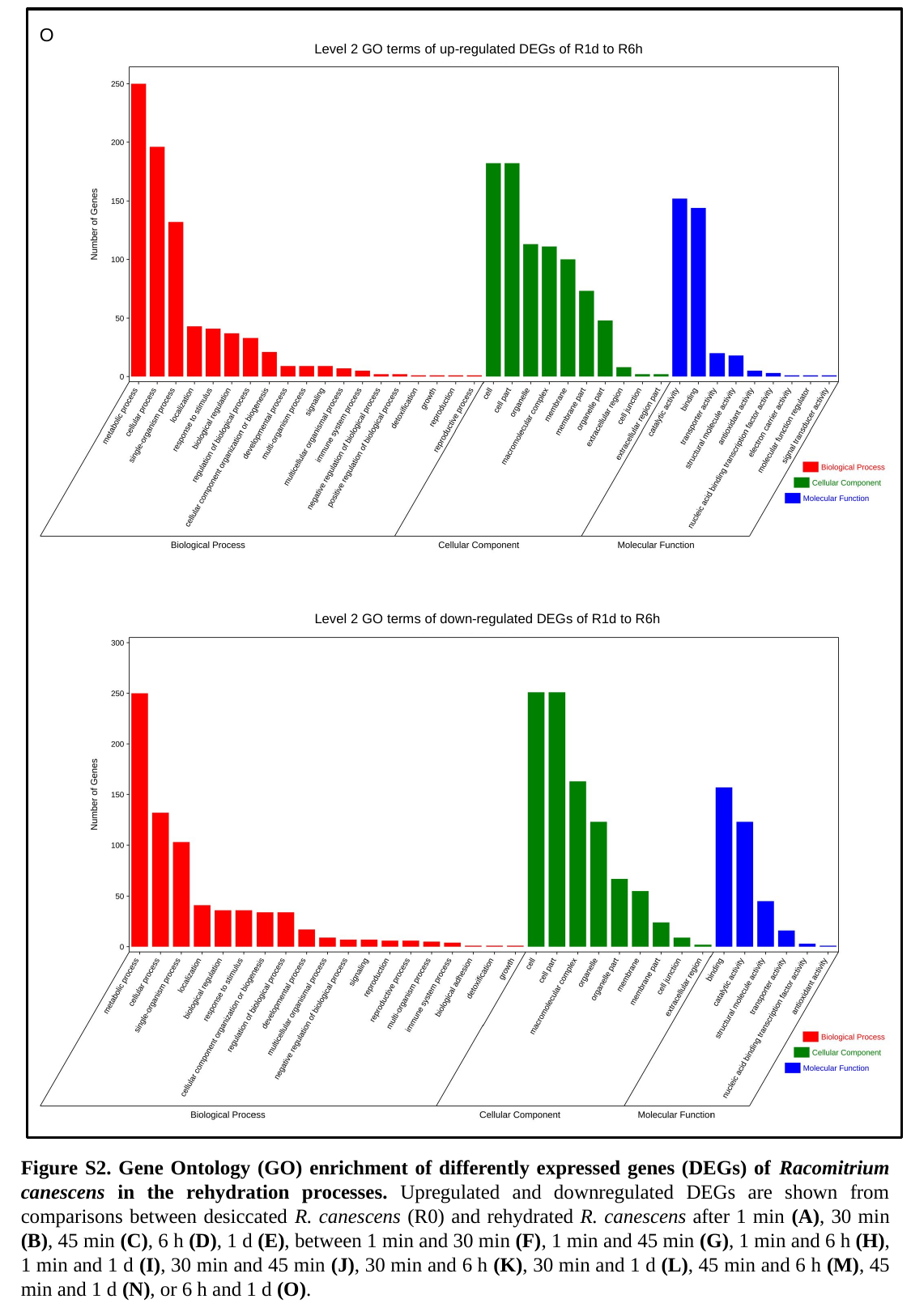

O
Level 2 GO terms of up-regulated DEGs of R1d to R6h
Level 2 GO terms of down-regulated DEGs of R1d to R6h
Figure S2. Gene Ontology (GO) enrichment of differently expressed genes (DEGs) of Racomitrium canescens in the rehydration processes. Upregulated and downregulated DEGs are shown from comparisons between desiccated R. canescens (R0) and rehydrated R. canescens after 1 min (A), 30 min (B), 45 min (C), 6 h (D), 1 d (E), between 1 min and 30 min (F), 1 min and 45 min (G), 1 min and 6 h (H), 1 min and 1 d (I), 30 min and 45 min (J), 30 min and 6 h (K), 30 min and 1 d (L), 45 min and 6 h (M), 45 min and 1 d (N), or 6 h and 1 d (O).
