# Supplementary material for: Proteomic and Transcriptomic Responses of the Desiccation-Tolerant Moss Racomitrium canescens in the Rapid Rehydration Processes
Source: Genes (Basel). 2023 Feb 2;14(2):390. doi: 10.3390/genes14020390 (PMC9956249; doi:10.3390/genes14020390)
Supplement: Supplementary file 1 [file genes-14-00390-s001.zip › figure S3.pptx]

## Slide 1
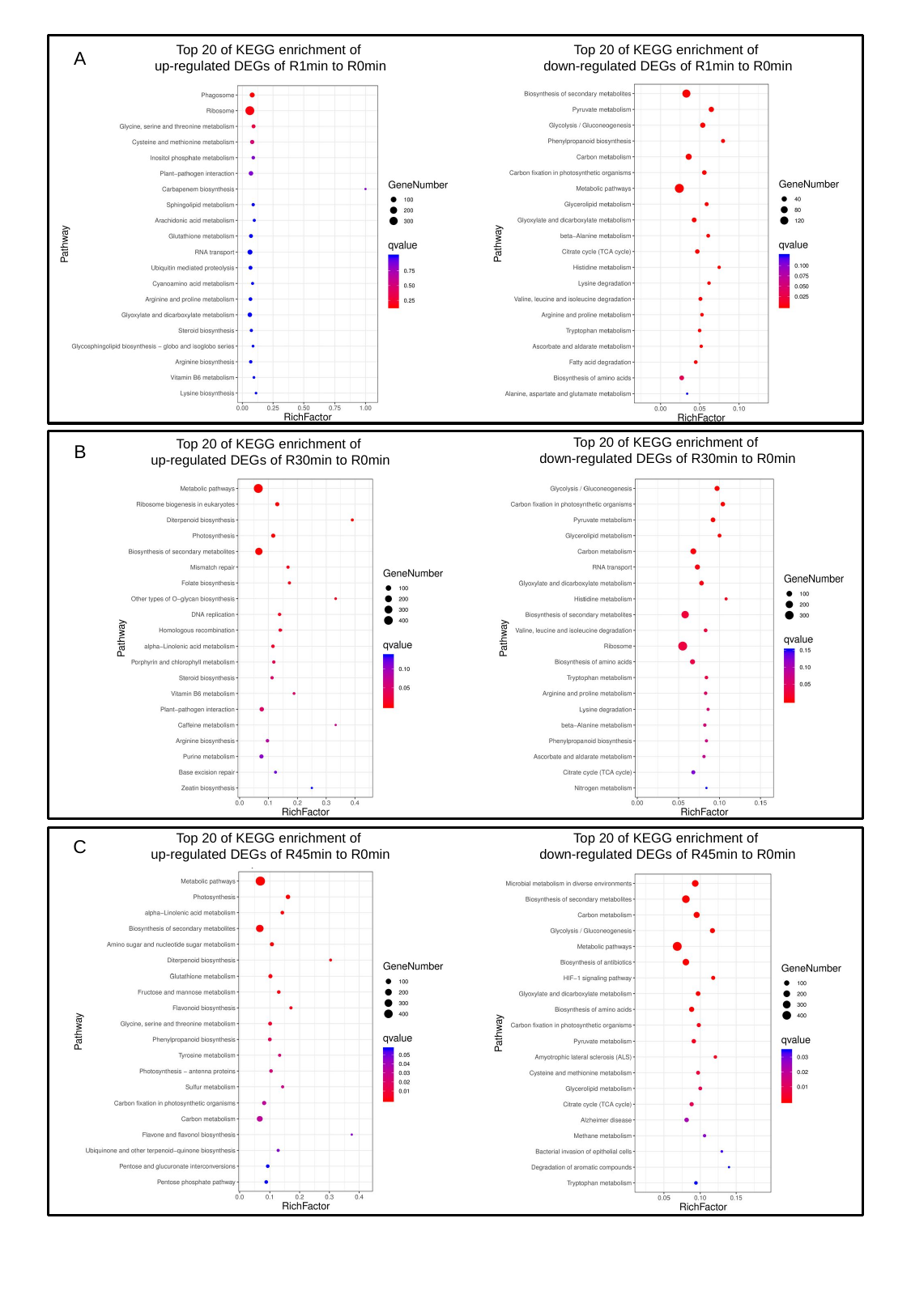

Top 20 of KEGG enrichment of
 up-regulated DEGs of R1min to R0min
Top 20 of KEGG enrichment of
down-regulated DEGs of R1min to R0min
A
Top 20 of KEGG enrichment of
down-regulated DEGs of R30min to R0min
Top 20 of KEGG enrichment of
up-regulated DEGs of R30min to R0min
B
Top 20 of KEGG enrichment of
up-regulated DEGs of R45min to R0min
Top 20 of KEGG enrichment of
 down-regulated DEGs of R45min to R0min
C

## Slide 2
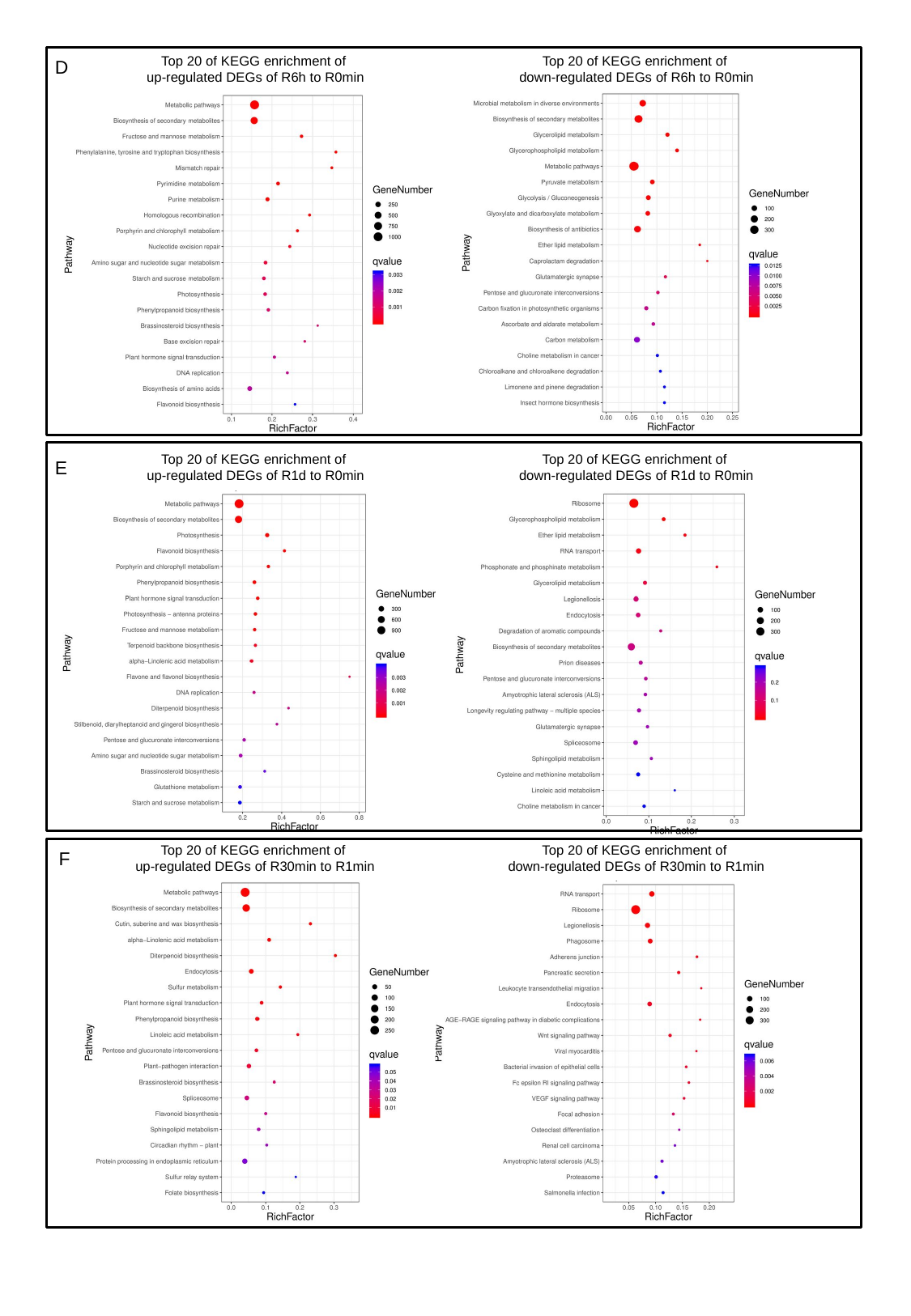

Top 20 of KEGG enrichment of
down-regulated DEGs of R6h to R0min
Top 20 of KEGG enrichment of
 up-regulated DEGs of R6h to R0min
D
Top 20 of KEGG enrichment of
up-regulated DEGs of R1d to R0min
Top 20 of KEGG enrichment of
down-regulated DEGs of R1d to R0min
E
Top 20 of KEGG enrichment of
 down-regulated DEGs of R30min to R1min
Top 20 of KEGG enrichment of
 up-regulated DEGs of R30min to R1min
F

## Slide 3
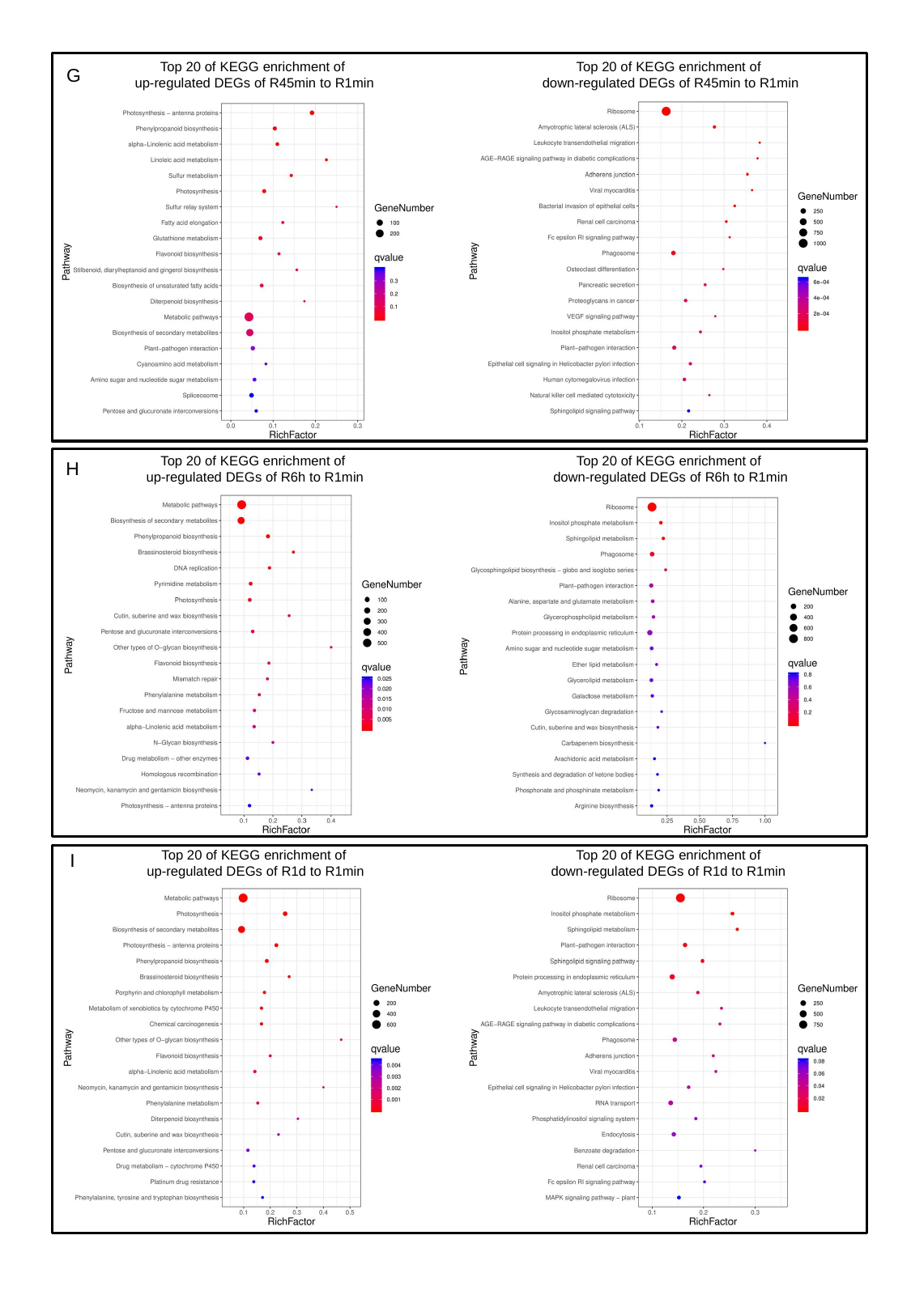

Top 20 of KEGG enrichment of
down-regulated DEGs of R45min to R1min
Top 20 of KEGG enrichment of
 up-regulated DEGs of R45min to R1min
G
Top 20 of KEGG enrichment of
 up-regulated DEGs of R6h to R1min
Top 20 of KEGG enrichment of
 down-regulated DEGs of R6h to R1min
H
Top 20 of KEGG enrichment of
 up-regulated DEGs of R1d to R1min
Top 20 of KEGG enrichment of
down-regulated DEGs of R1d to R1min
I

## Slide 4
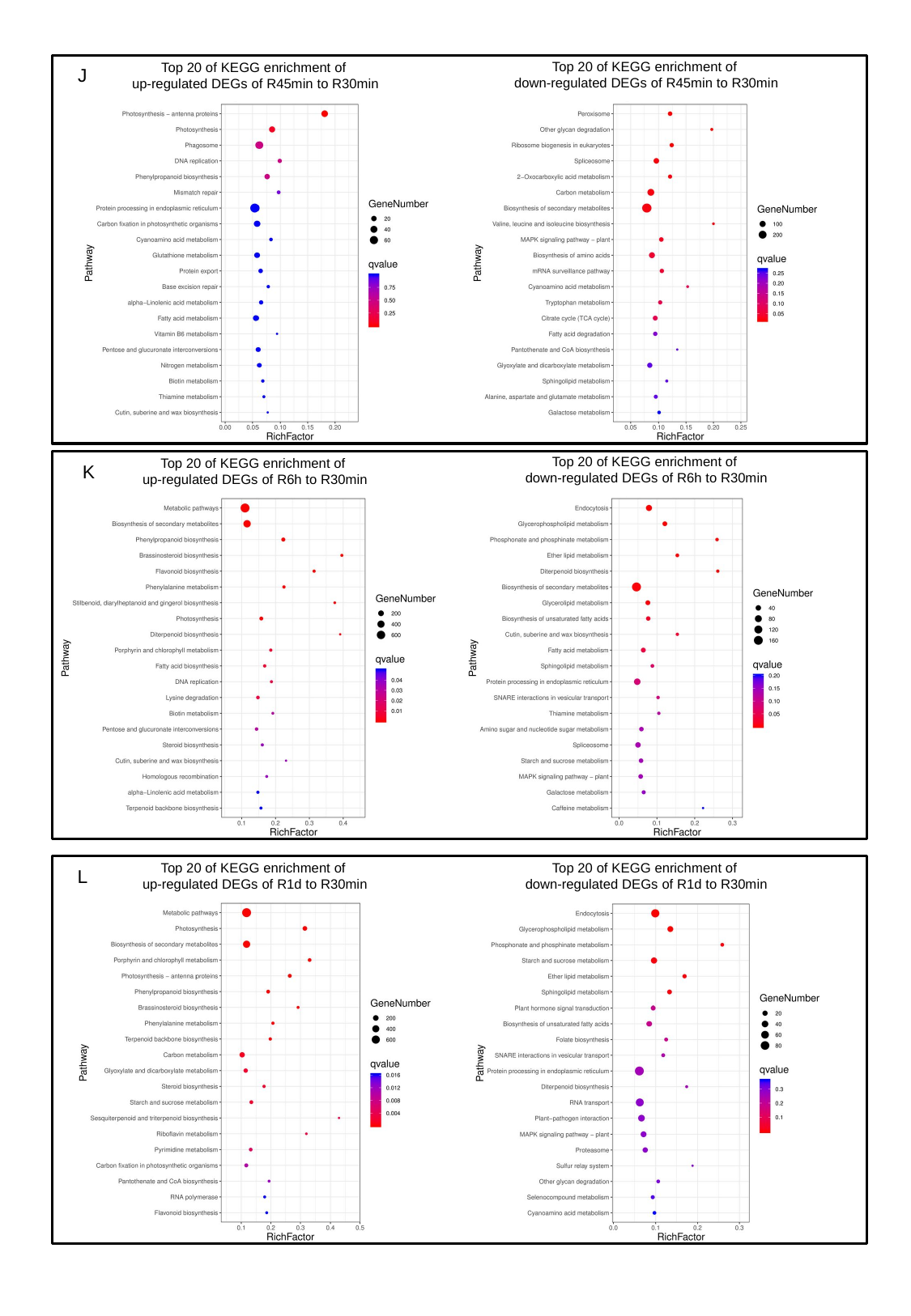

Top 20 of KEGG enrichment of
down-regulated DEGs of R45min to R30min
Top 20 of KEGG enrichment of
up-regulated DEGs of R45min to R30min
J
Top 20 of KEGG enrichment of
down-regulated DEGs of R6h to R30min
Top 20 of KEGG enrichment of
 up-regulated DEGs of R6h to R30min
K
Top 20 of KEGG enrichment of
up-regulated DEGs of R1d to R30min
Top 20 of KEGG enrichment of
down-regulated DEGs of R1d to R30min
L

## Slide 5
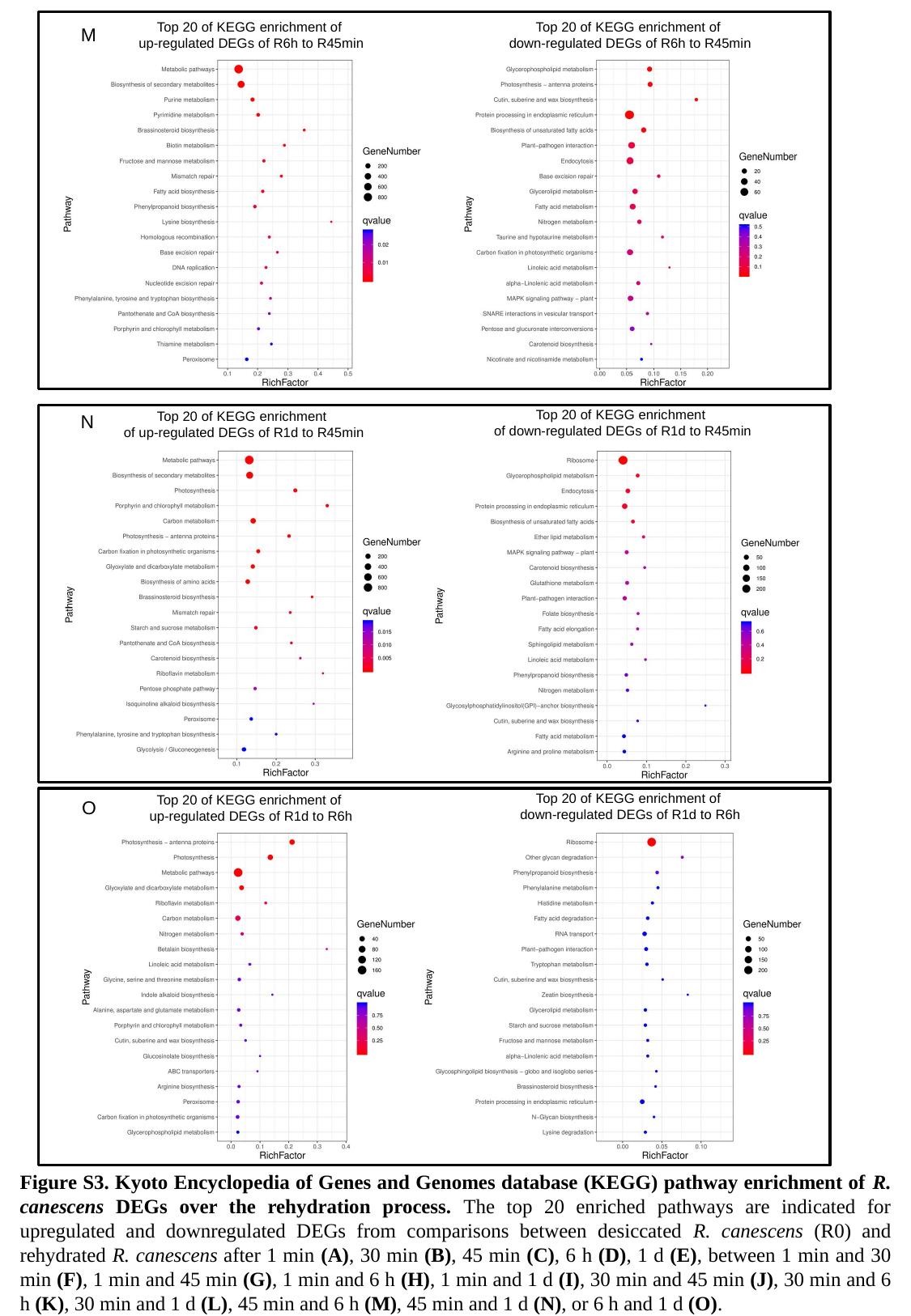

Top 20 of KEGG enrichment of
 up-regulated DEGs of R6h to R45min
Top 20 of KEGG enrichment of
down-regulated DEGs of R6h to R45min
M
Top 20 of KEGG enrichment
of down-regulated DEGs of R1d to R45min
Top 20 of KEGG enrichment
of up-regulated DEGs of R1d to R45min
N
Top 20 of KEGG enrichment of
down-regulated DEGs of R1d to R6h
Top 20 of KEGG enrichment of
 up-regulated DEGs of R1d to R6h
O
Figure S3. Kyoto Encyclopedia of Genes and Genomes database (KEGG) pathway enrichment of R. canescens DEGs over the rehydration process. The top 20 enriched pathways are indicated for upregulated and downregulated DEGs from comparisons between desiccated R. canescens (R0) and rehydrated R. canescens after 1 min (A), 30 min (B), 45 min (C), 6 h (D), 1 d (E), between 1 min and 30 min (F), 1 min and 45 min (G), 1 min and 6 h (H), 1 min and 1 d (I), 30 min and 45 min (J), 30 min and 6 h (K), 30 min and 1 d (L), 45 min and 6 h (M), 45 min and 1 d (N), or 6 h and 1 d (O).
