# Supplementary material for: Proteomic and Transcriptomic Responses of the Desiccation-Tolerant Moss Racomitrium canescens in the Rapid Rehydration Processes
Source: Genes (Basel). 2023 Feb 2;14(2):390. doi: 10.3390/genes14020390 (PMC9956249; doi:10.3390/genes14020390)
Supplement: Supplementary file 1 [file genes-14-00390-s001.zip › figure S4.pptx]

## Slide 1
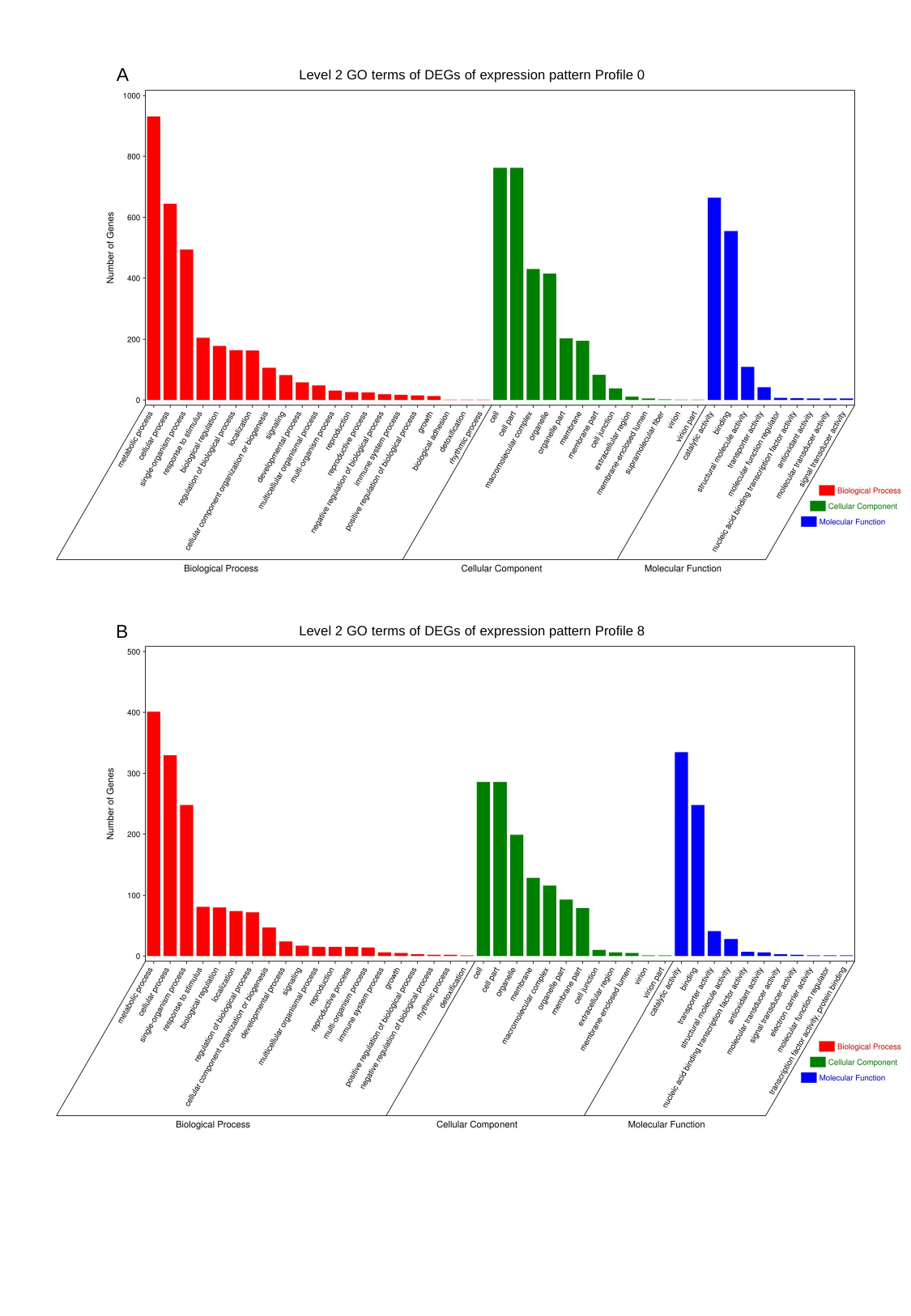

A
Level 2 GO terms of DEGs of expression pattern Profile 0
B
Level 2 GO terms of DEGs of expression pattern Profile 8

## Slide 2
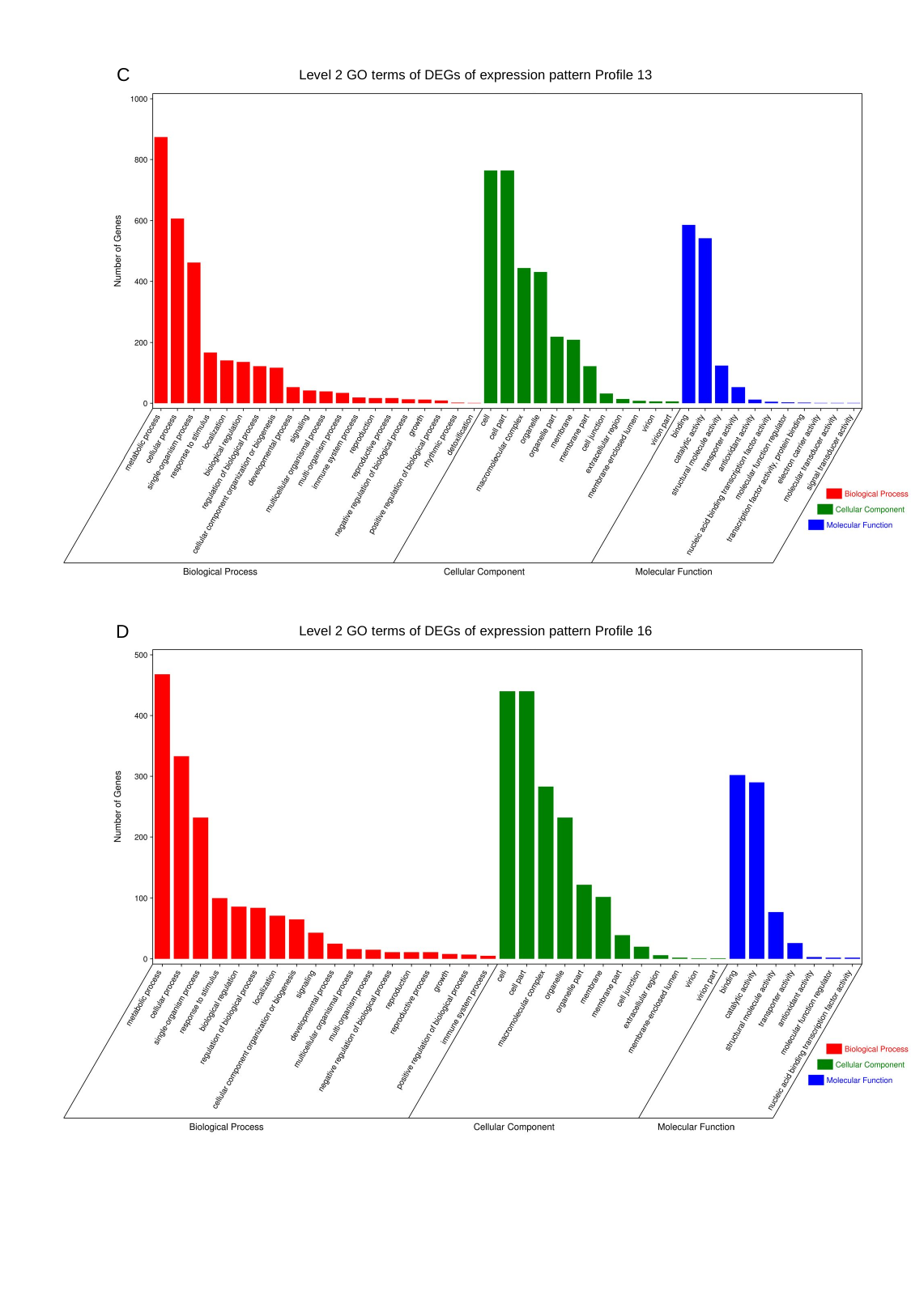

C
Level 2 GO terms of DEGs of expression pattern Profile 13
D
Level 2 GO terms of DEGs of expression pattern Profile 16

## Slide 3
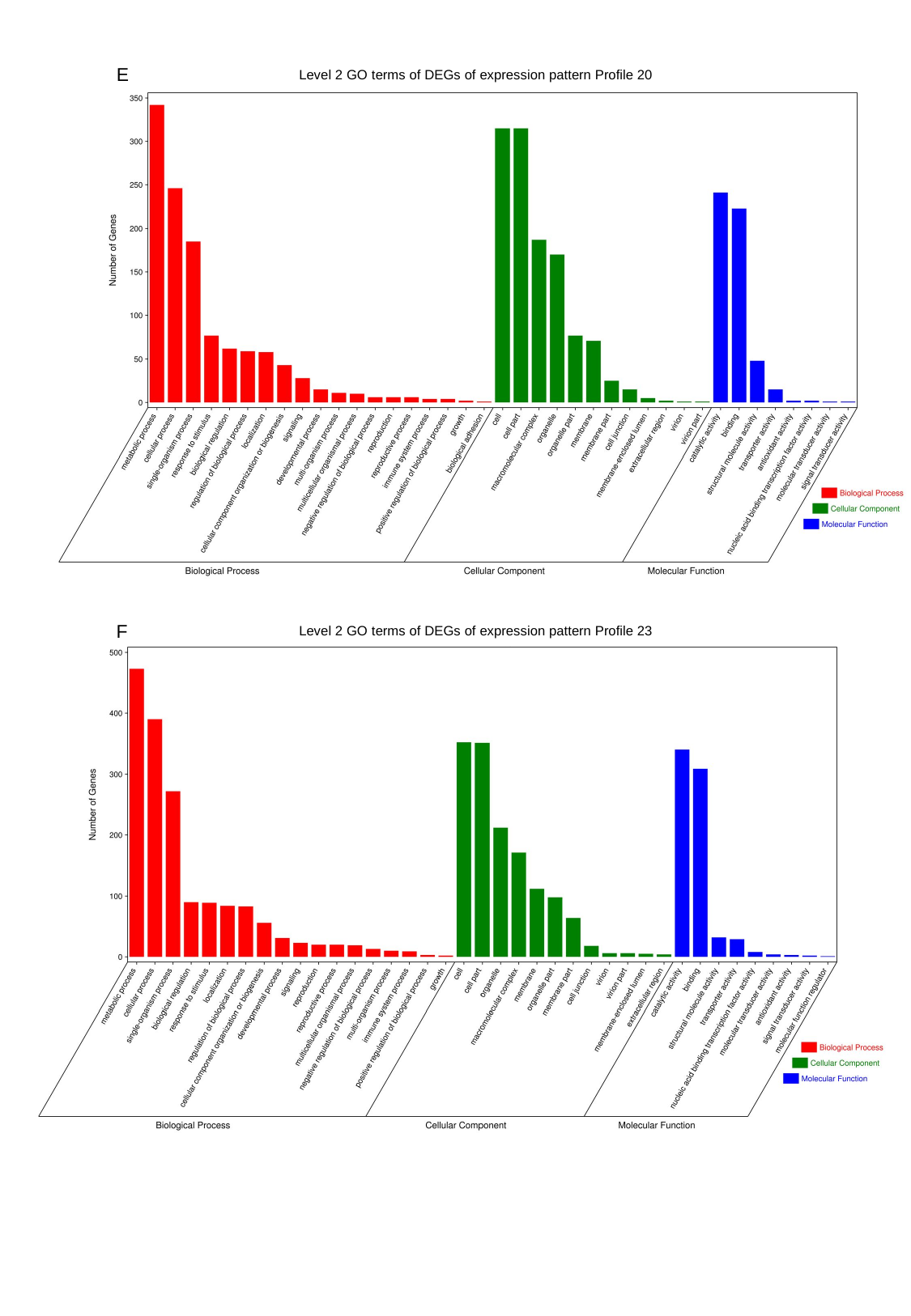

E
Level 2 GO terms of DEGs of expression pattern Profile 20
F
Level 2 GO terms of DEGs of expression pattern Profile 23

## Slide 4
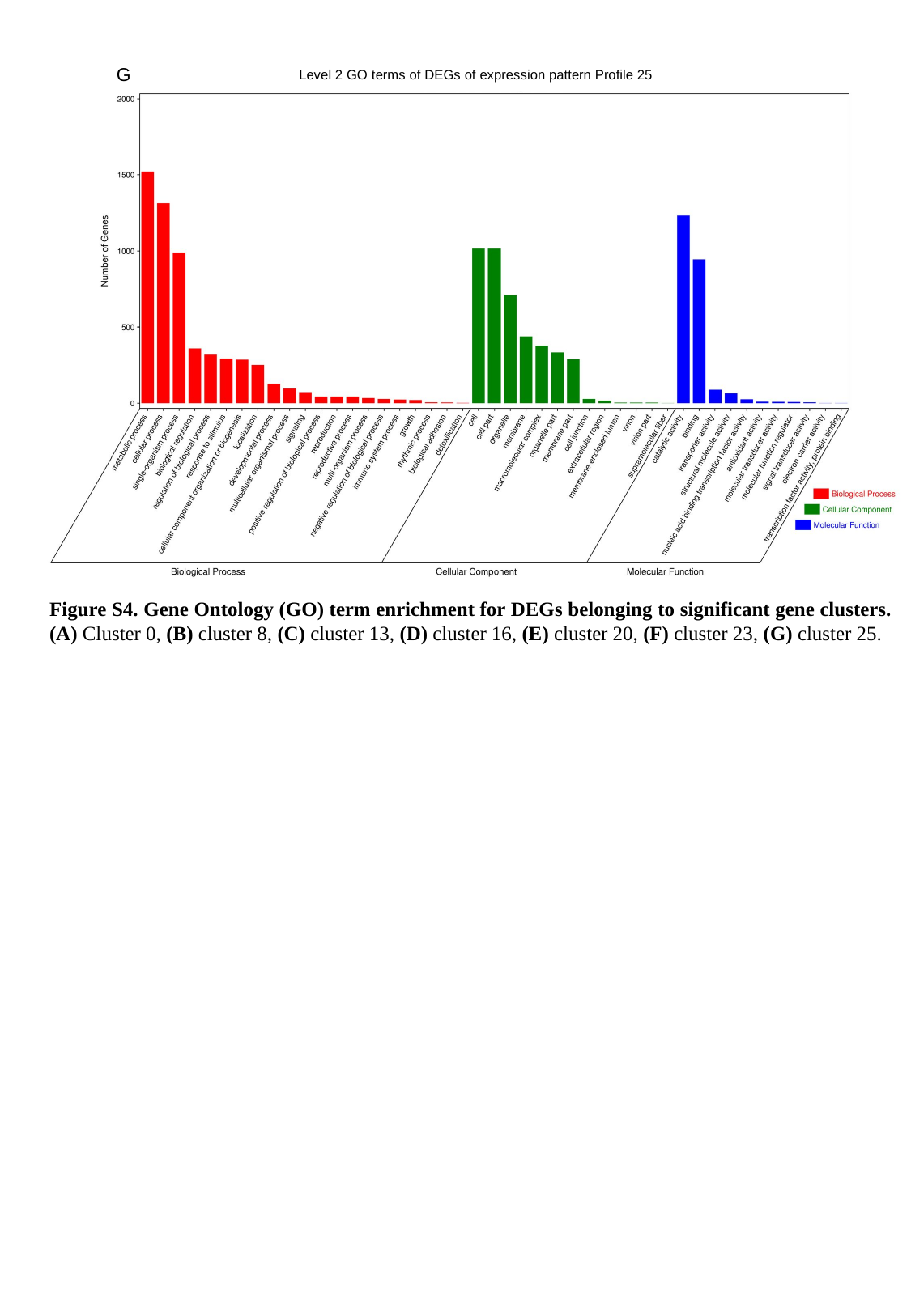

G
Level 2 GO terms of DEGs of expression pattern Profile 25
Figure S4. Gene Ontology (GO) term enrichment for DEGs belonging to significant gene clusters. (A) Cluster 0, (B) cluster 8, (C) cluster 13, (D) cluster 16, (E) cluster 20, (F) cluster 23, (G) cluster 25.
