# Supplementary material for: Proteomic and Transcriptomic Responses of the Desiccation-Tolerant Moss Racomitrium canescens in the Rapid Rehydration Processes
Source: Genes (Basel). 2023 Feb 2;14(2):390. doi: 10.3390/genes14020390 (PMC9956249; doi:10.3390/genes14020390)
Supplement: Supplementary file 1 [file genes-14-00390-s001.zip › figure S5.pptx]

## Slide 1
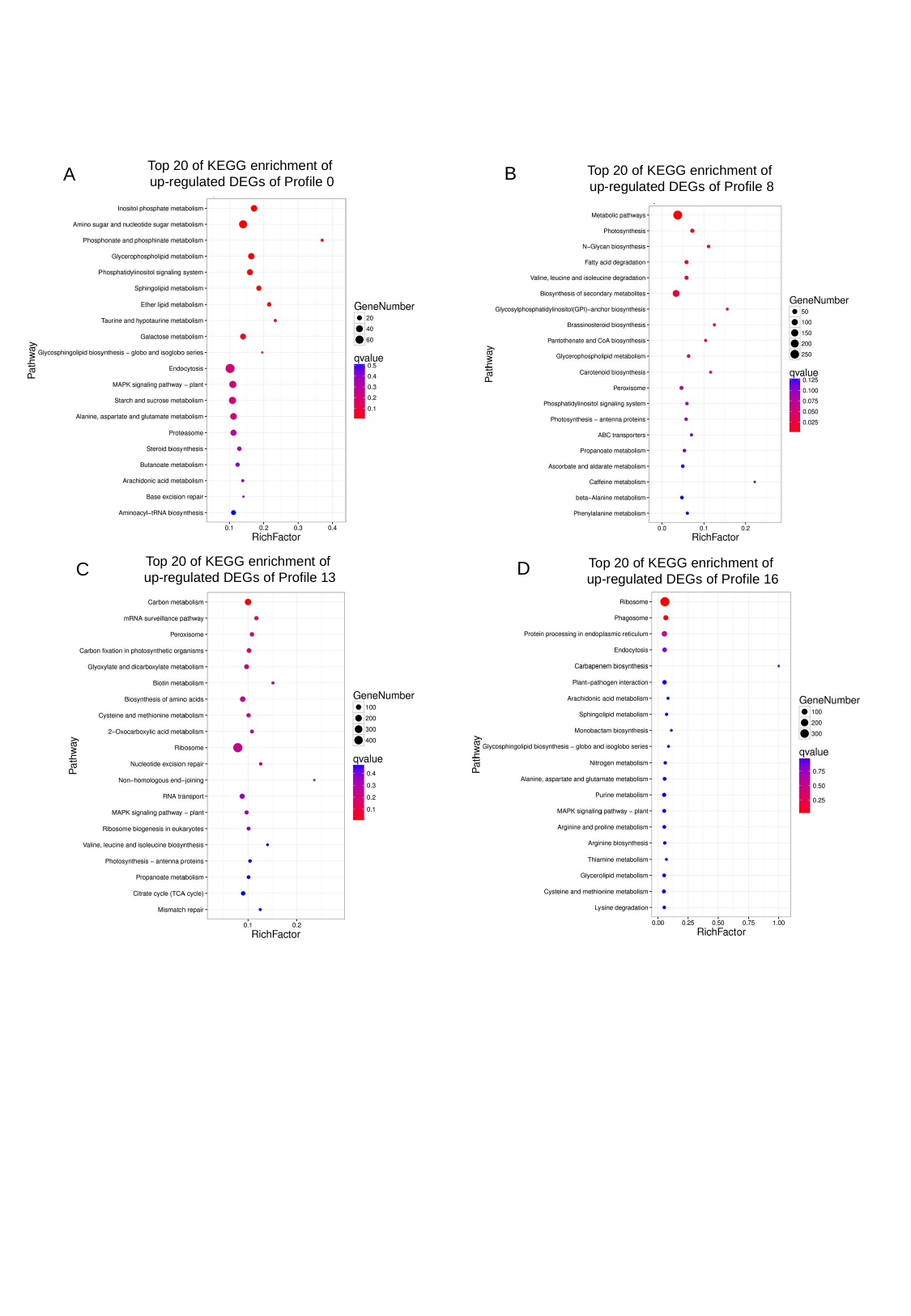

Top 20 of KEGG enrichment of
 up-regulated DEGs of Profile 0
B
Top 20 of KEGG enrichment of
 up-regulated DEGs of Profile 8
A
Top 20 of KEGG enrichment of
 up-regulated DEGs of Profile 13
Top 20 of KEGG enrichment of
 up-regulated DEGs of Profile 16
D
C

## Slide 2
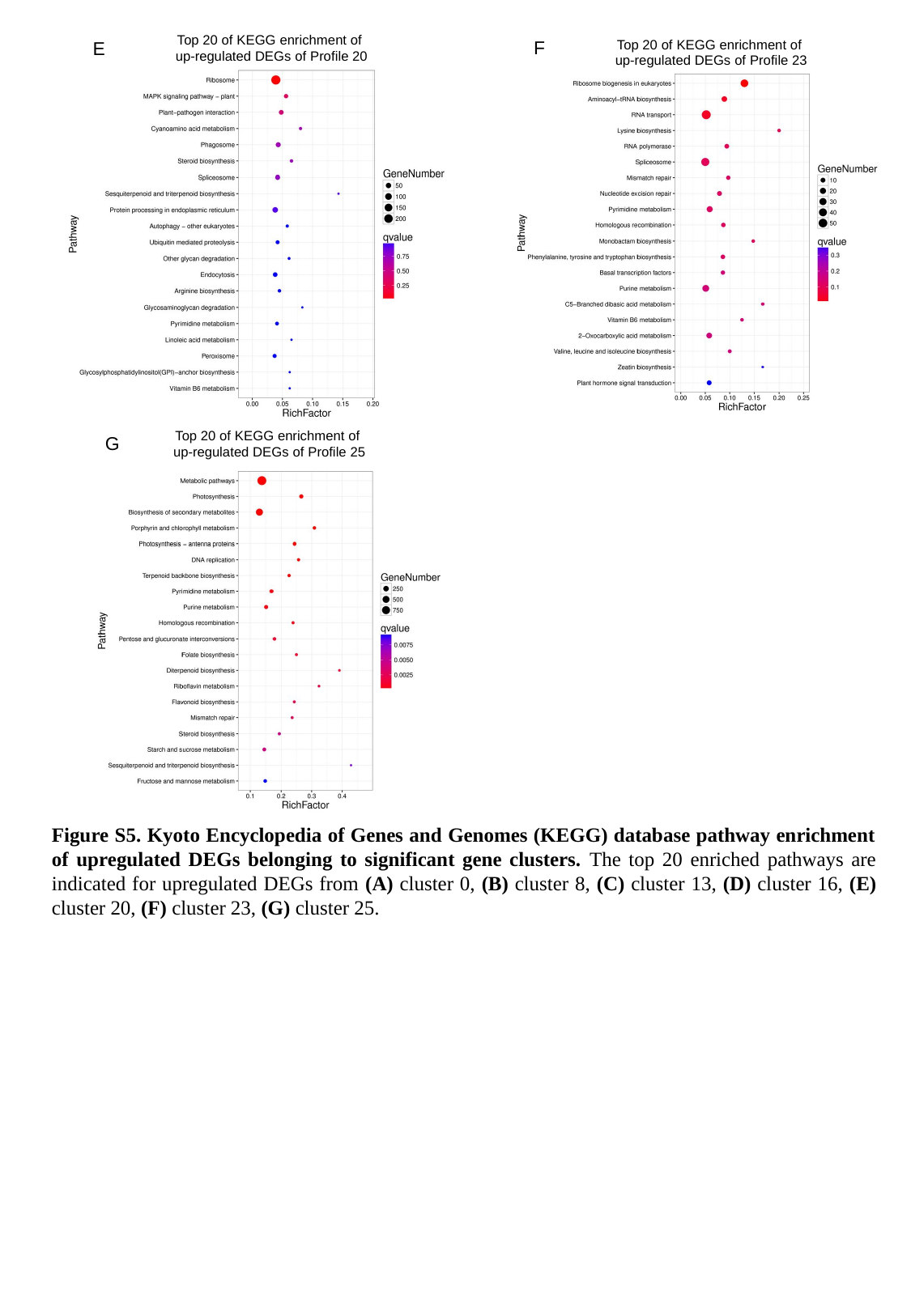

Top 20 of KEGG enrichment of
 up-regulated DEGs of Profile 20
F
Top 20 of KEGG enrichment of
 up-regulated DEGs of Profile 23
E
Top 20 of KEGG enrichment of
 up-regulated DEGs of Profile 25
G
Figure S5. Kyoto Encyclopedia of Genes and Genomes (KEGG) database pathway enrichment of upregulated DEGs belonging to significant gene clusters. The top 20 enriched pathways are indicated for upregulated DEGs from (A) cluster 0, (B) cluster 8, (C) cluster 13, (D) cluster 16, (E) cluster 20, (F) cluster 23, (G) cluster 25.
