# Supplementary material for: Proteomic and Transcriptomic Responses of the Desiccation-Tolerant Moss Racomitrium canescens in the Rapid Rehydration Processes
Source: Genes (Basel). 2023 Feb 2;14(2):390. doi: 10.3390/genes14020390 (PMC9956249; doi:10.3390/genes14020390)
Supplement: Supplementary file 1 [file genes-14-00390-s001.zip › figure S6.pptx]

## Slide 1
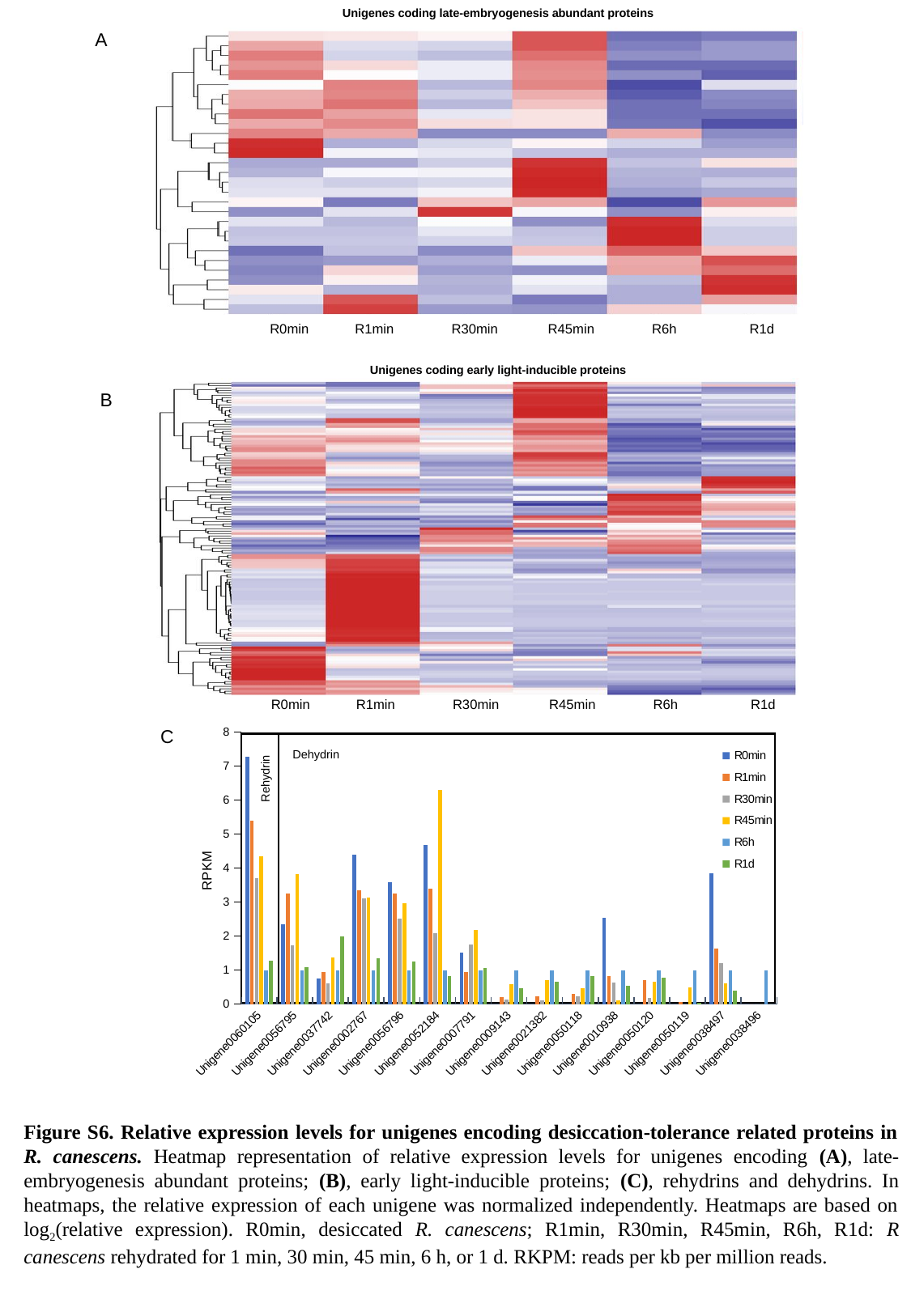

Unigenes coding late-embryogenesis abundant proteins
A
R0min R1min R30min R45min R6h R1d
B
R0min R1min R30min R45min R6h R1d
C
### Chart
| Category | R0min | R1min | R30min | R45min | R6h | R1d |
|---|---|---|---|---|---|---|
| Unigene0060105 | 7.273218576014128 | 5.391756181208385 | 3.6980628608154964 | 4.3429677270596185 | 1.0 | 1.2665733980215732 |
| Unigene0056795 | 2.3485202026974057 | 3.2496688818881414 | 1.7181466548628597 | 3.8206350911539975 | 1.0 | 1.094563027767317 |
| Unigene0037742 | 0.740194036465294 | 0.9492794319826011 | 0.6101520150398385 | 1.3676735047151574 | 1.0 | 1.9837818472930056 |
| Unigene0002767 | 4.392423505245998 | 3.3548192457344306 | 3.1056372253376283 | 3.1212104775867515 | 1.0 | 1.347590532295926 |
| Unigene0056796 | 3.5812914293377744 | 3.2603387890586504 | 2.505476402940548 | 2.968729189465217 | 1.0 | 1.2613953839400296 |
| Unigene0052184 | 4.6708218385804 | 3.3986022039216084 | 2.0883027346848944 | 6.28638185270679 | 1.0 | 0.8149314921255612 |
| Unigene0007791 | 1.5217915455155444 | 0.9314538134645493 | 1.7485596063520463 | 2.1899105345560277 | 1.0 | 1.0499082979199283 |
| Unigene0009143 | 0.0 | 0.21086269325823323 | 0.12293809989079833 | 0.5789413184665785 | 1.0 | 0.4628541870222427 |
| Unigene0021382 | 0.0 | 0.21611582268567212 | 0.11300319989259103 | 0.7101523864933207 | 1.0 | 0.6660251963570454 |
| Unigene0050118 | 0.0 | 0.3099272107789995 | 0.2278457487997522 | 0.4680192039646895 | 1.0 | 0.8145268700634969 |
| Unigene0010938 | 2.53946065487193 | 0.8322468312648535 | 0.634563968840771 | 0.10307466332189069 | 1.0 | 0.5346827964087668 |
| Unigene0050120 | 0.0 | 0.7096356973485666 | 0.17492994179780127 | 0.6555292088812245 | 1.0 | 0.7836818279801682 |
| Unigene0050119 | 0.0 | 0.04946866984243313 | 0.0 | 0.4932209600586296 | 1.0 | 0.0491022352510077 |
| Unigene0038497 | 3.8578224101479908 | 1.6374207188160677 | 1.2034883720930232 | 0.617336152219873 | 1.0 | 0.38530655391120505 |
| Unigene0038496 | 0.0 | 0.0 | 0.0 | 0.0 | 1.0 | 0.0 |
Dehydrin
Rehydrin
RPKM
Unigenes coding early light-inducible proteins
Figure S6. Relative expression levels for unigenes encoding desiccation-tolerance related proteins in R. canescens. Heatmap representation of relative expression levels for unigenes encoding (A), late-embryogenesis abundant proteins; (B), early light-inducible proteins; (C), rehydrins and dehydrins. In heatmaps, the relative expression of each unigene was normalized independently. Heatmaps are based on log2(relative expression). R0min, desiccated R. canescens; R1min, R30min, R45min, R6h, R1d: R canescens rehydrated for 1 min, 30 min, 45 min, 6 h, or 1 d. RKPM: reads per kb per million reads.
