# Supplementary material for: Proteomic and Transcriptomic Responses of the Desiccation-Tolerant Moss Racomitrium canescens in the Rapid Rehydration Processes
Source: Genes (Basel). 2023 Feb 2;14(2):390. doi: 10.3390/genes14020390 (PMC9956249; doi:10.3390/genes14020390)
Supplement: Supplementary file 1 [file genes-14-00390-s001.zip › figure S7.pptx]

## Slide 1
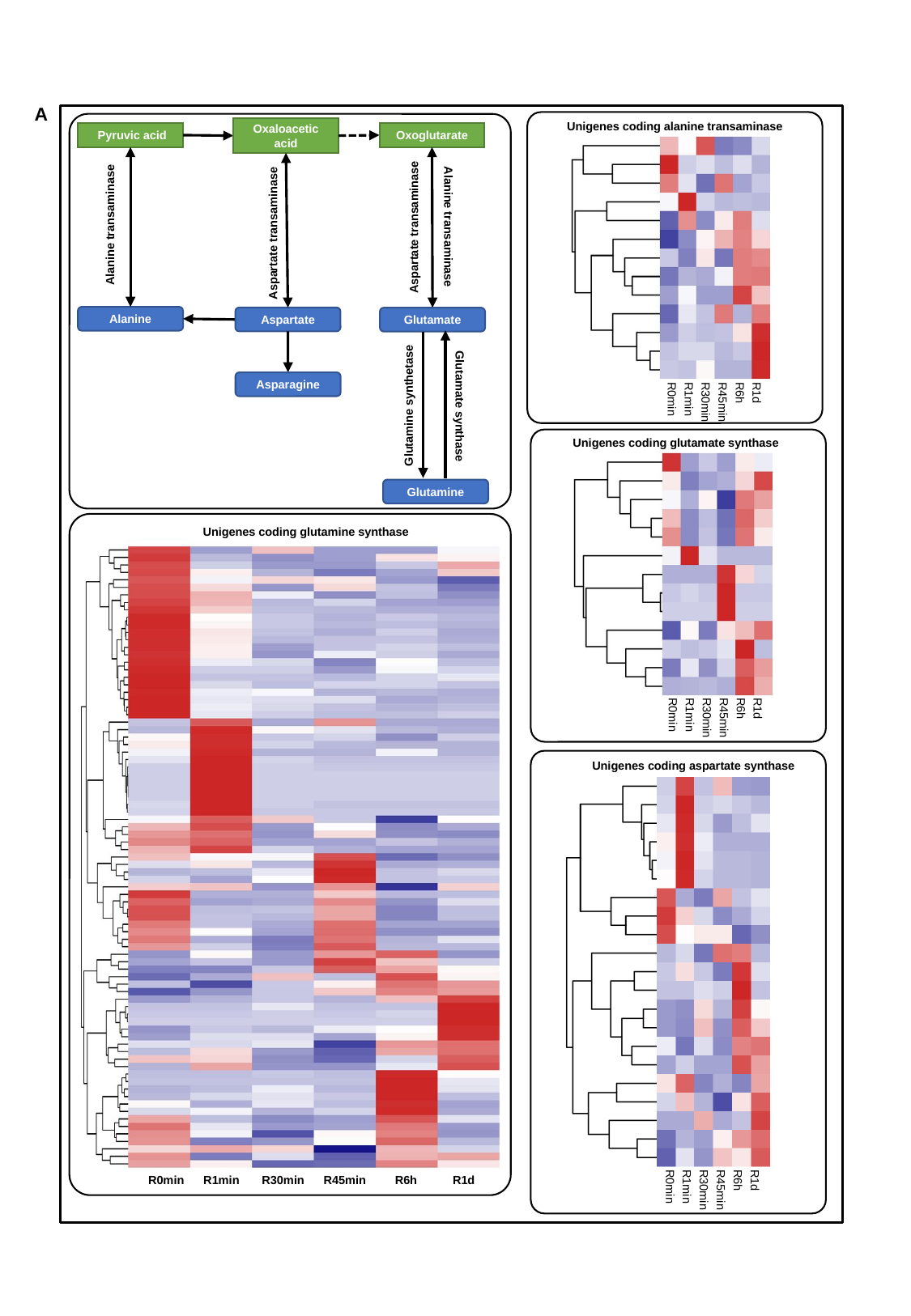

A
Unigenes coding alanine transaminase
Oxaloacetic acid
 Pyruvic acid
Oxoglutarate
Alanine transaminase
Alanine transaminase
Aspartate transaminase
Aspartate transaminase
Alanine
Aspartate
Glutamate
Asparagine
Glutamine synthetase
Glutamate synthase
Glutamine
R1d
R6h
R45min
R30min
R1min
R0min
Unigenes coding glutamate synthase
Unigenes coding glutamine synthase
R1d
R6h
R45min
R30min
R1min
R0min
Unigenes coding aspartate synthase
R1d
R6h
R45min
R30min
R1min
R0min
R0min R1min R30min R45min R6h R1d

## Slide 2
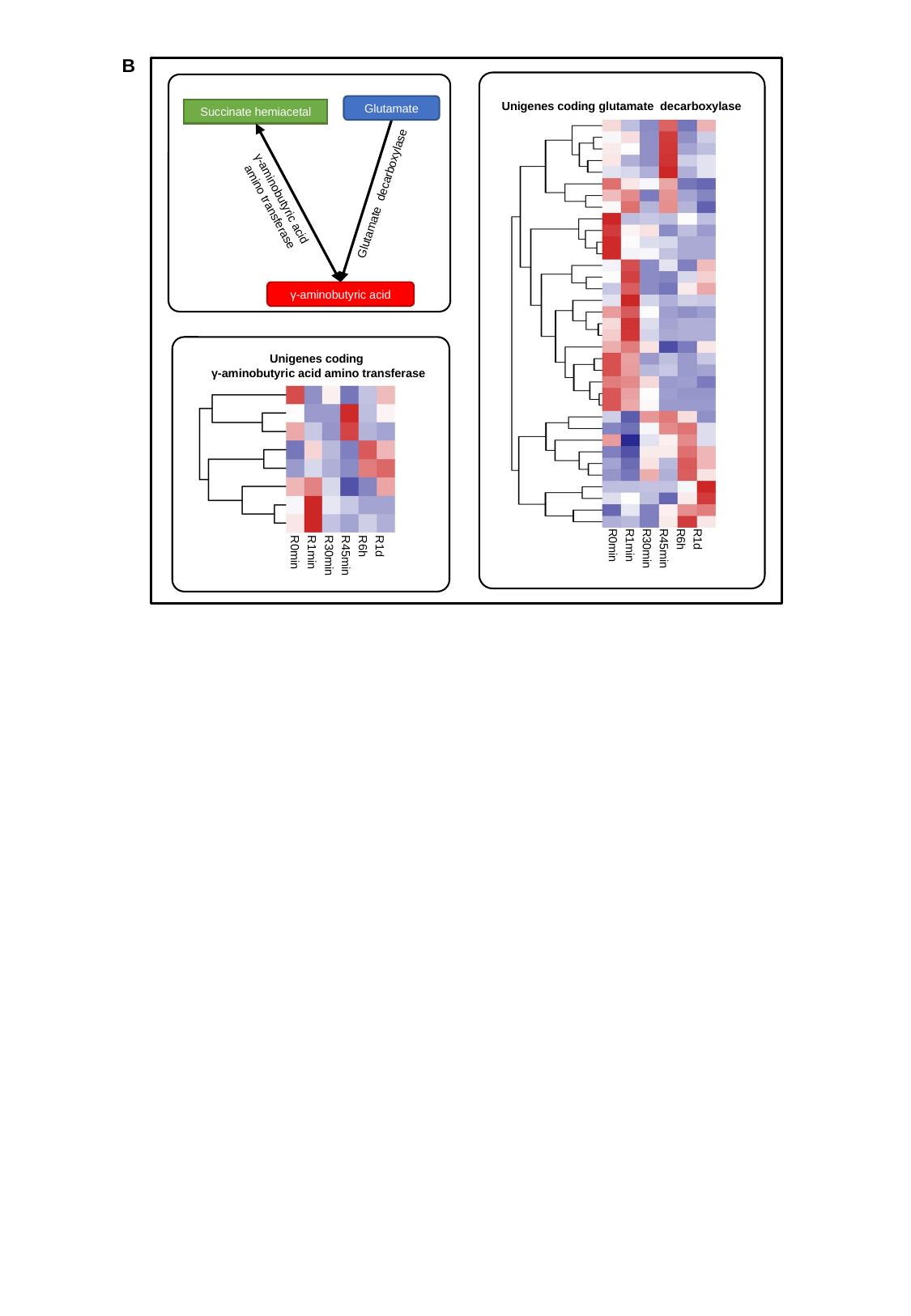

B
Unigenes coding glutamate decarboxylase
Glutamate
Succinate hemiacetal
Glutamate decarboxylase
γ-aminobutyric acid
amino transferase
γ-aminobutyric acid
Unigenes coding
γ-aminobutyric acid amino transferase
R1d
R6h
R45min
R30min
R1min
R0min
R1d
R6h
R45min
R30min
R1min
R0min

## Slide 3
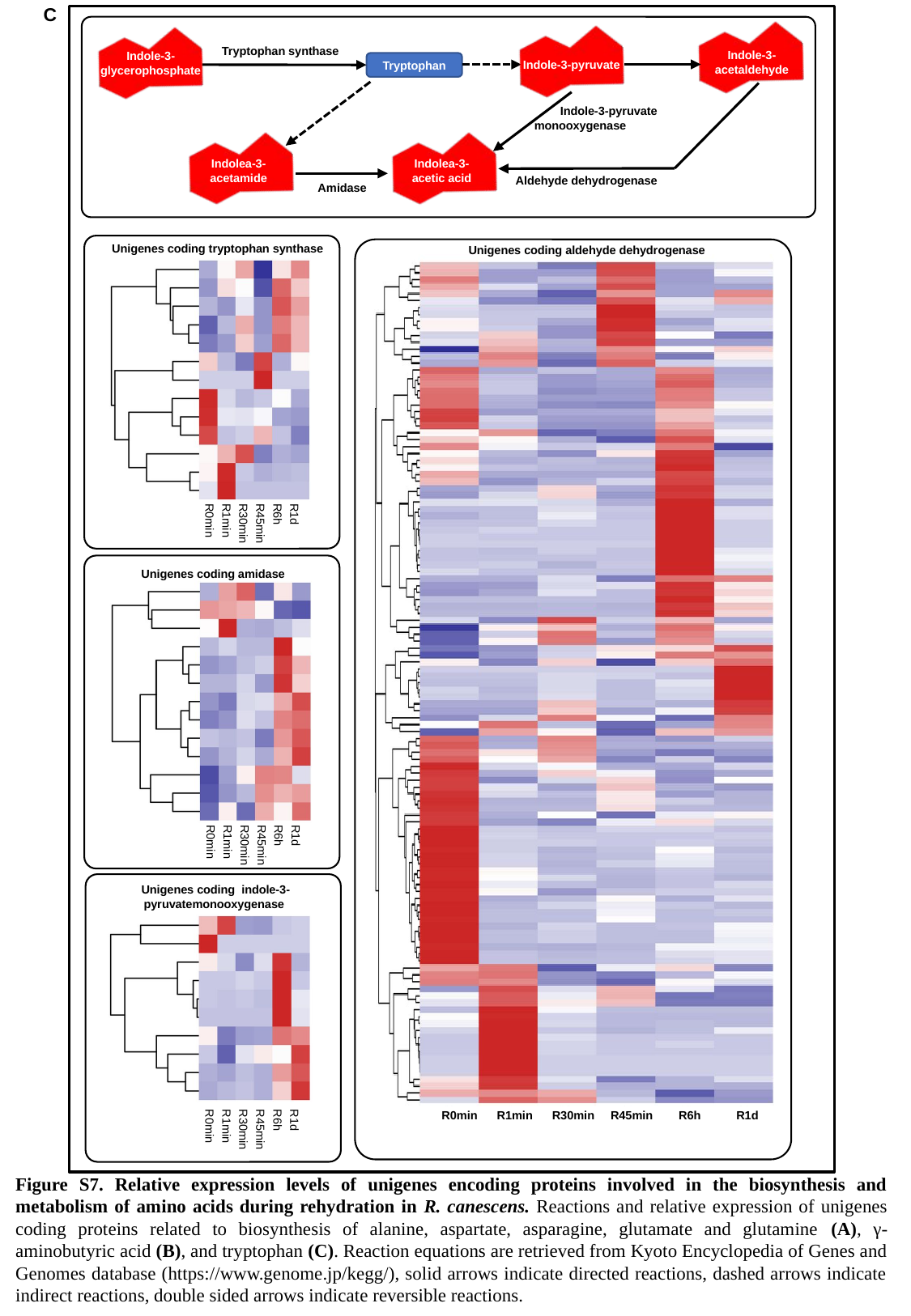

C
Indole-3-
acetaldehyde
Indole-3-pyruvate
Indole-3-
glycerophosphate
Tryptophan synthase
Tryptophan
 Indole-3-pyruvate monooxygenase
Indolea-3-
acetamide
Indolea-3-
 acetic acid
Aldehyde dehydrogenase
Amidase
Unigenes coding tryptophan synthase
R1d
R6h
R45min
R30min
R1min
R0min
Unigenes coding aldehyde dehydrogenase
Unigenes coding amidase
R1d
R6h
R45min
R30min
R1min
R0min
Unigenes coding indole-3-pyruvatemonooxygenase
R1d
R6h
R45min
R30min
R1min
R0min
R0min R1min R30min R45min R6h R1d
Figure S7. Relative expression levels of unigenes encoding proteins involved in the biosynthesis and metabolism of amino acids during rehydration in R. canescens. Reactions and relative expression of unigenes coding proteins related to biosynthesis of alanine, aspartate, asparagine, glutamate and glutamine (A), γ-aminobutyric acid (B), and tryptophan (C). Reaction equations are retrieved from Kyoto Encyclopedia of Genes and Genomes database (https://www.genome.jp/kegg/), solid arrows indicate directed reactions, dashed arrows indicate indirect reactions, double sided arrows indicate reversible reactions.
