# Supplementary material for: Proteomic and Transcriptomic Responses of the Desiccation-Tolerant Moss Racomitrium canescens in the Rapid Rehydration Processes
Source: Genes (Basel). 2023 Feb 2;14(2):390. doi: 10.3390/genes14020390 (PMC9956249; doi:10.3390/genes14020390)
Supplement: Supplementary file 1 [file genes-14-00390-s001.zip › figure S8.pptx]

## Slide 1
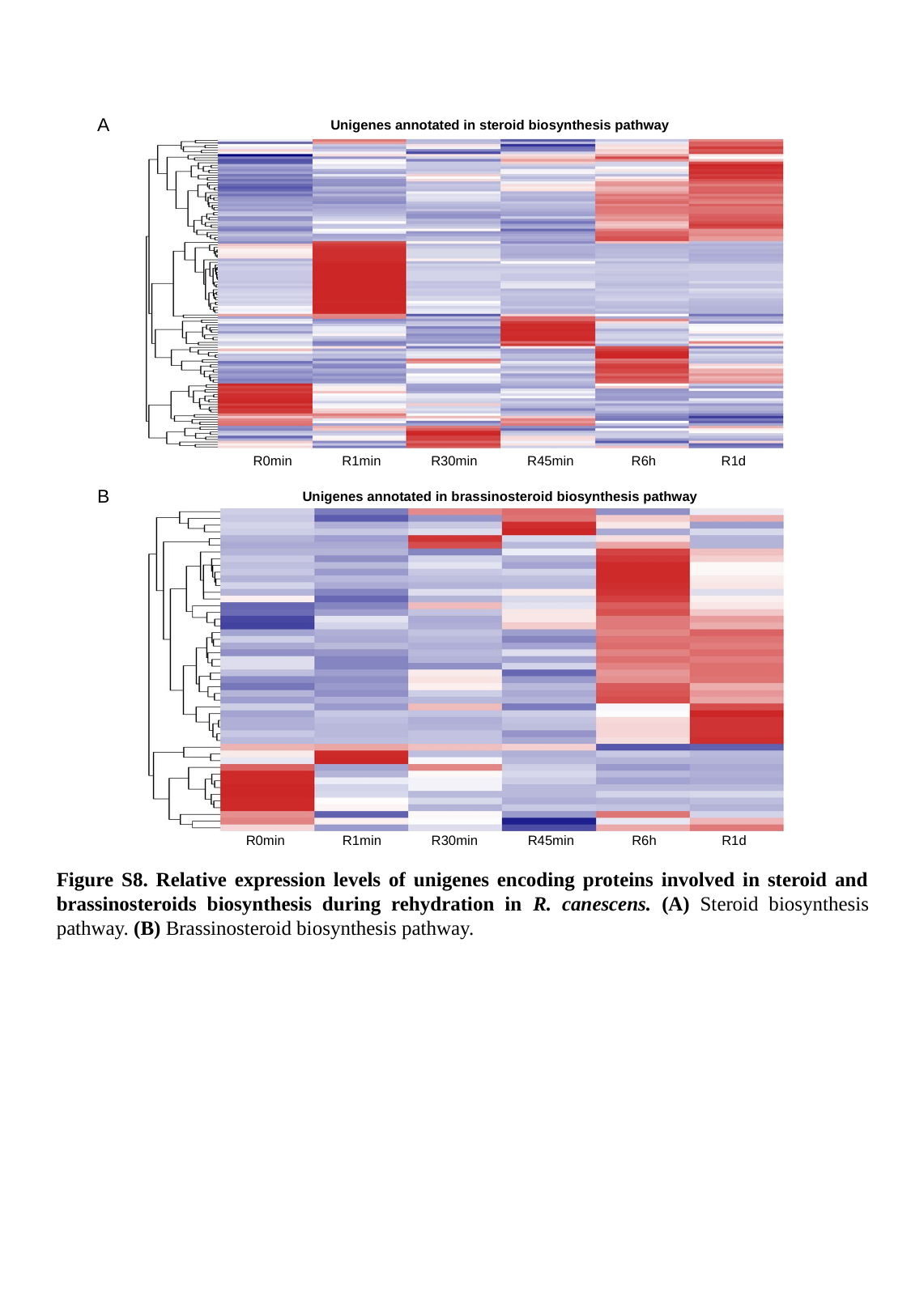

A
Unigenes annotated in steroid biosynthesis pathway
R0min R1min R30min R45min R6h R1d
B
Unigenes annotated in brassinosteroid biosynthesis pathway
R0min R1min R30min R45min R6h R1d
Figure S8. Relative expression levels of unigenes encoding proteins involved in steroid and brassinosteroids biosynthesis during rehydration in R. canescens. (A) Steroid biosynthesis pathway. (B) Brassinosteroid biosynthesis pathway.
