# Supplementary material for: Proteomic and Transcriptomic Responses of the Desiccation-Tolerant Moss Racomitrium canescens in the Rapid Rehydration Processes
Source: Genes (Basel). 2023 Feb 2;14(2):390. doi: 10.3390/genes14020390 (PMC9956249; doi:10.3390/genes14020390)
Supplement: Supplementary file 1 [file genes-14-00390-s001.zip › figure S9 .pptx]

## Slide 1
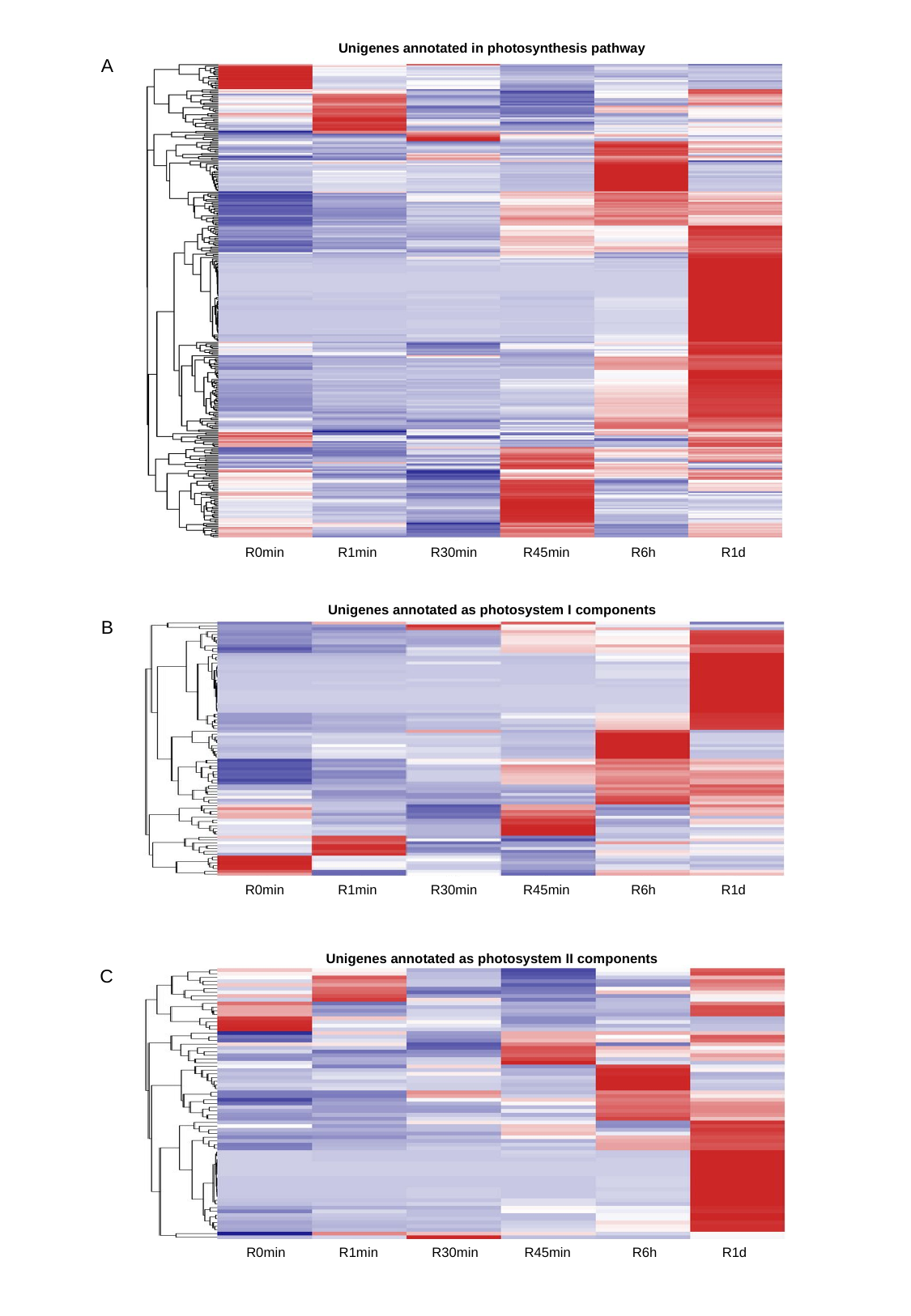

Unigenes annotated in photosynthesis pathway
A
R0min R1min R30min R45min R6h R1d
Unigenes annotated as photosystem I components
B
R0min R1min R30min R45min R6h R1d
Unigenes annotated as photosystem II components
C
R0min R1min R30min R45min R6h R1d

## Slide 2
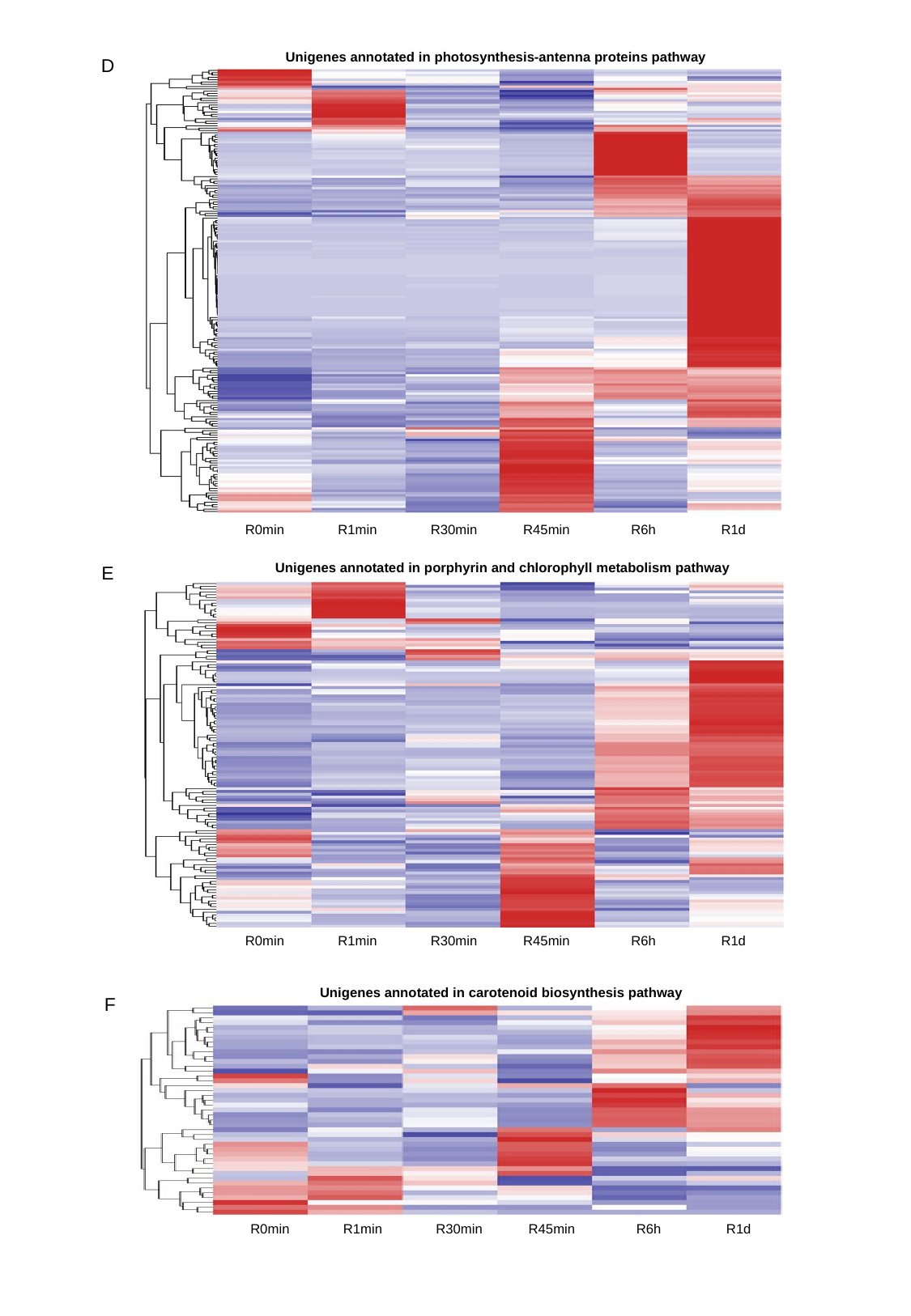

Unigenes annotated in photosynthesis-antenna proteins pathway
D
R0min R1min R30min R45min R6h R1d
Unigenes annotated in porphyrin and chlorophyll metabolism pathway
E
R0min R1min R30min R45min R6h R1d
Unigenes annotated in carotenoid biosynthesis pathway
F
R0min R1min R30min R45min R6h R1d

## Slide 3
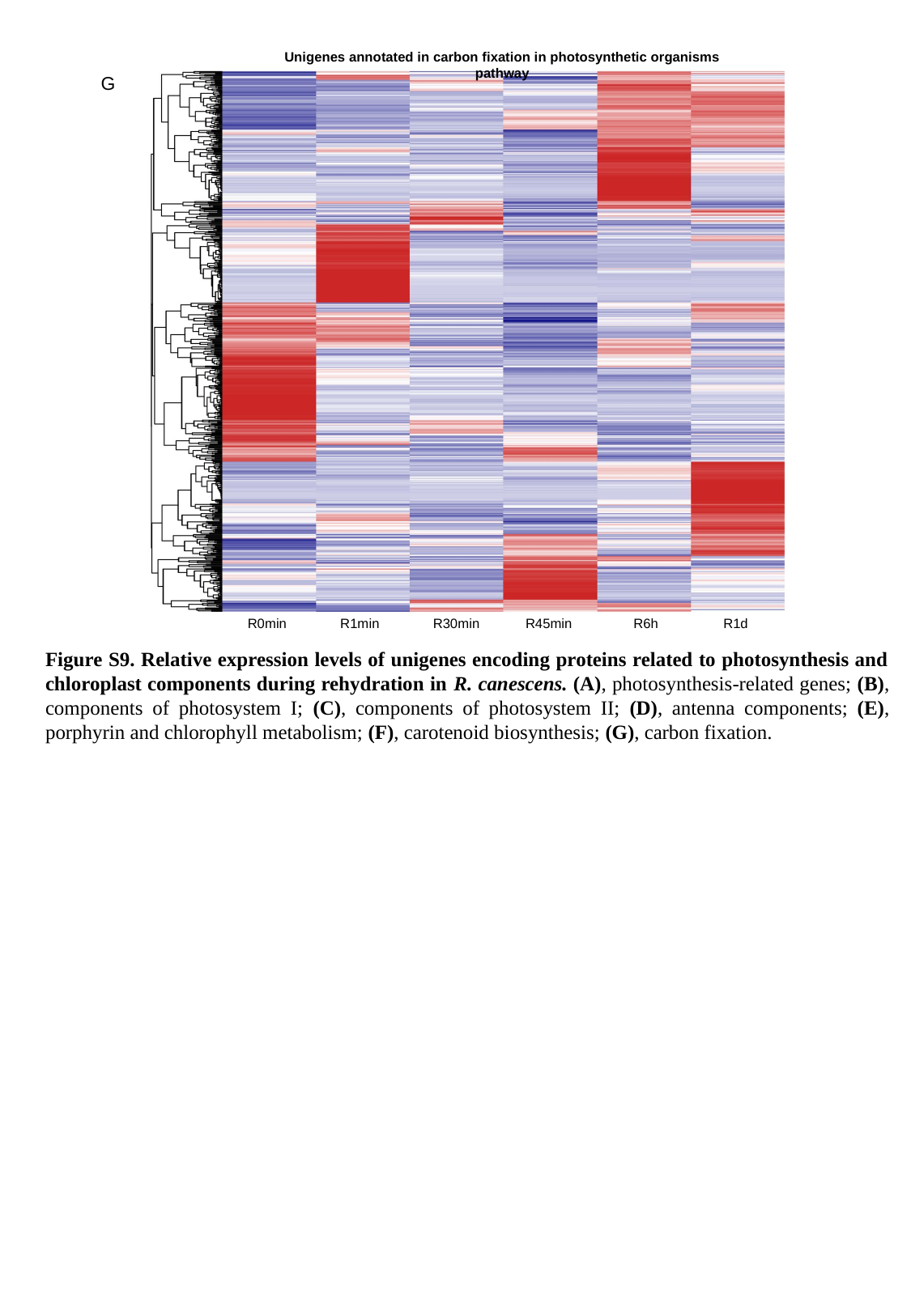

Unigenes annotated in carbon fixation in photosynthetic organisms pathway
G
R0min R1min R30min R45min R6h R1d
Figure S9. Relative expression levels of unigenes encoding proteins related to photosynthesis and chloroplast components during rehydration in R. canescens. (A), photosynthesis-related genes; (B), components of photosystem I; (C), components of photosystem II; (D), antenna components; (E), porphyrin and chlorophyll metabolism; (F), carotenoid biosynthesis; (G), carbon fixation.
