# Supplementary material for: Proteomic and Transcriptomic Responses of the Desiccation-Tolerant Moss Racomitrium canescens in the Rapid Rehydration Processes
Source: Genes (Basel). 2023 Feb 2;14(2):390. doi: 10.3390/genes14020390 (PMC9956249; doi:10.3390/genes14020390)
Supplement: Supplementary file 1 [file genes-14-00390-s001.zip › Supplementary Tables-20230110.pdf]

Table S1. List of primers used in qRT-PCR analysis.

Unigene	Forward primer sequence	Reverse primer sequence
Unigene0044326	AGCCTCCGCATTCTGTTCG	CTCCCAGCACGCATTCCC
Unigene0003124	CGTTGGAGGAAGTTGTGGAG	GCTTGTAACCGCCGAAGG
Unigene0009458	GCAACACCAGCACCGAGAG	CGCCACGAACAGGAACCAG
Unigene0009493	TGCTGTGCGGTGGAGATGATG	GCGGACTCGGACGGAAGATG
Unigene0010441	GCCGCTGTTCTTTGCTGTG	GGAATGGAATGAGTTGTTAGTTG
Unigene0043420	ACCAACTGCGGCGACAAG	CACCTGGACGACGACGATG
Unigene0046204	GCCGAGGTGAAGCGTATGG	TTCTTGAGGAGCGAAGGATGC
Unigene0050808	GGCGGAGCGGAAGCGGAAG	CGAGGACCTTACGGAACGACTT
Unigene0055922	GCGTGGATATGGCGAAGTG	GTCGGCGTTGTCAGTTCC
Unigene0056205	GAGTGTTGAAGGATTACGAAG	ATGCCGATGGTGATGAAGAAC
Unigene0056795	GGCTCCAACACAGGCTACC	TGTCGTCACTTCCAACTCTCG
Unigene0058161	ACGGAGATATTGAAGACACAG	TCATAACCGATGGCTTGGAAGG
Unigene0060105	CCGAACTCAGGCTACGACAC	TCCTTCATCTTATCCACCACACC

Table S2. Number of peptides and proteins identified in desiccated *Racomitrium canescens* of different rehydration stages.

Sample	Identified proteins	Identified peptides	Identified spectra
R0min-1	2 677	11 878	18 028
R0min-2	2 667	11 674	17 942
R0min-3	2 568	11 127	17 352
R1min-1	1 815	7 462	11 758
R1min-2	1 497	5 690	8 926
R1min-3	1 745	7 184	11 156
R6h-1	1 590	6 081	9 204
R6h-2	1 794	6 914	10 722
R6h-3	1 706	6 681	10 559

Table S3. Functional annotation of proteins with increased accumulation in *Racomitrium canescens* after 1 min rehydration.

Categories/annotation	Unigene ID	Mean iBAQ		
		R0min	R1min	R6h
**Protease and peptidase**				
Subtilisin-like protease	0061971	3753133.3	9377266.7	3194533.3
Aspartic proteinase-like protein 2	0058435	3050500	4545833.3*	4925266.7
Aminopeptidase M1	0024169	2434633.3	3545133.3	3797066.7
Serine carboxypeptidase	0032483	1581220	3060133.3	4358300
Presequence protease 1, chloroplastic/mitochondrial-like isoform	0007650	1547233.3	2342366.7*	2744033.3
Puromycin-sensitive aminopeptidase isoform X1	0061910	1003443.3	1653366.7*	2829466.7
Low-temperature-induced cysteine proteinase	0011055	1067796.7	1261966.7	1765833.3
Leucine aminopeptidase 2, chloroplastic	0038457	382420	1138693.3	609550
Subtilisin-like protease	0015368	212820	920570*	934406.67
Serine carboxypeptidase-like enzyme	0056687	471380	833586.67	1277066.7
Aspartic proteinase-like protein 2 isoform X2	0002662	191866.67	471250*	795800
Subtilisin-like protease	0061547	246406.67	422796.67	609606.67*
Beta-aspartyl peptidase	0010440	0	200686.67*	194831.67*
Probable glutamyl endopeptidase, chloroplastic	0049098	37624.667	116488*	76838.667
**Saccharides degradation/metabolism**				
Alpha-amylase	0050221	109231.33	310554.67	266123.33
Alpha-galactosidase 3	0064191	74550.333	116676.67*	190510
Alpha-mannosidase	0065028	414980	1046416.7	1655200
Alpha-mannosidase isoform X1	0062067	108553.33	420846.67	705513.33
Alpha-xylosidase 1-like	0010441	600780	1030330*	1643933.3
Beta-fructofuranosidase	0046488	727190	2020300*	2704833.3
Beta-galactosidase 9	0006714	129650	280663.33	412123.33
Endo-1,3;1,4-beta-D-glucanase-like isoform X2	0007756	1198200	2509900*	3394700
Glucan endo-1,3-beta-glucosidase 5-like	0040173	0	74551.333*	189996.67*
Alpha-xylosidase 1	0043926	349780	643040*	916100
Mannosylglycoprotein endo-beta-mannosidase-like	0056704	190663.33	345830*	445400
Trehalase	0053334	264080	1096053.3*	1818900
**Redox regulation**				
Lambda class glutathione S-transferase	0052456	522463.33	586026.67	124996.67
Mitochondrial Mn-superoxide dismutase	0049856	8791200	13273333	16125000
Peroxidase	0043835	88252667	130136667*	122654000
Peroxidase	0023388	60326667	85263667	41602333
**Chromatin structure composition**				
Histone H2B	0002626	0	22434667*	16999933*
Histone H4	0030563	6273400	73637667*	89975000
Nucleosome assembly protein family	0010541	0	346980*	632673.33*

Table S3. Continued

Categories/annotation	Unigene ID	Mean iBAQ		
		R0min	R1min	R6h
**Extracellular matrix substance**				
Chitinase	0076039	792553.33	1012393.3	1442966.7
Laccase-3-like	0043730	478780	1129756.7*	1147806.7
Pectinesterase-like	0049397	2008400	3076366.7	13015667
**Cytoskeleton related proteins**				
Actin, plasmodial isoform	0065906	227230	429976.67	121983.67
Armadillo repeat-containing kinesin A	0003222	57459.667	99289.667	107107.33
Beta-1 tubulin	0062682	187422.67	276800	505766.67
Severin	0054408	0	39208*	51564*
Tropomyosin-1	0062660	110066.67	650920	1454233.3
**Lipid metabolism**				
Lipocalin-like protein	0024096	1579613.3	3637433.3*	2670066.7
Phospholipase D alpha 1	0034363	2442200	3437600	3599933.3
Phospholiase C	0055013	160123.33	481880*	380893.33
GDSL esterase/lipase	0037669	0	472420*	410050*
Probable plastid-lipid-associated protein	0059364	0	44749.333*	0
Temperature-induced lipocalin	0022374	330806.67	1004320*	1347916.7*
**Putative stress related proteins**				
Nascent polypeptide-associated complex subunit alpha-like protein	0064085	0	171056.67*	222016.67*
Niemann-Pick C1 protein	0062046	33968.333	61226*	40817
Blue copper protein	0052403	0	541066.67*	649040*
LRR receptor-like serine/threonine-protein kinase EFR	0037797	0	1582333.3*	654836.67*
Pyruvate decarboxylase 2 isoform X2	0039848	0	36131.333*	57091.5*
Glyoxalase_2 domain-containing protein	0003434	1481566.7	1829660	8862733.3
**Ribosome related proteins**				
Elongation factor 1-alpha 1	0004972	0	37558.667*	13311*
Elongation factor 1-alpha	0064711	0	26960.667*	35390*
60S ribosomal protein L15	0094090	122216.67*	325356.67*	500320*
60S ribosomal protein L15	0078401	0	171566.67*	287715*
116 kDa U5 small nuclear ribonucleoprotein component	0039013	0	32912.333*	68247.667*
30S ribosomal protein S16-like	0030011	0	462276.67*	587220*
40S ribosomal protein S9-2	0054631	0	243200*	1200350*
60S ribosomal protein l23a	0052391	0	574900*	1087953.3*
Small nuclear ribonucleoprotein Sm D1-like	0071345	0	189115*	161456.67*
Ribosome maturation factor rimp	0023991	0	269460*	527345*
**Others**				
Indole-3-acetaldehyde oxidase isoform X7	0008118	234473.33	273623.33	693326.67

Table S3. Continued

Categories/annotation	Unigene ID	Mean iBAQ		
		R0min	R1min	R6h
Indole-3-acetaldehyde oxidase isoform X5	0001875	0	23935.5*	13429.667*
Metaxin-2, partial	0050100	0	155930*	162570*
Calcium-dependent lipid-binding domain-containing protein	0046387	1309600	14129000*	37727667
Chlorophyllase	0041131	5486166.7	6120300	16253000
Dihydrolipoyllysine-residue acetyltransferase component 3 of pyruvate dehydrogenase complex, mitochondrial-like isoform X3	0060878	556463.33	871273.33	144823
Glutamate dehydrogenase	0060503	280173.33	340333.33	688853.33
Isocitrate dehydrogenase [NADP], partial	0001540	0	90822.333*	97978.5*
Nucleoside diphosphate-sugar hydrolase of the mutt (NUDIX) family	0044702	319573.33	551486.67	714036.67
AT-hook motif nuclear-localized protein 9-like isoform X2	0053005	624590	1388206.7	3209400
Cell number regulator 10	0056278	1235440	1900876.7	2240566.7
Molybdopterin biosynthesis protein CNX1	0057532	116560	293970*	193061
Phosphoribosylformylglycinamidine cyclo-ligase, chloroplastic	0002576	405816.67	702583.33	807230
Polyamine oxidase	0041109	874220	1115386.7	1978666.7
Uric acid degradation bifunctional protein TTL X2	0050529	171973.33	561823.33	487760
Reticuline oxidase-like protein	0051668	1359260	1643100	2793566.7
14-3-3 protein zeta	0063616	286821.33	953256.67*	1167906.7
3-isopropylmalate dehydrogenase	0060015	165215.33	549920*	466200
Aminoacylase-1-like	0046482	382276.67	860683.33*	660600
Bac_rhamnosid-domain-containing protein	0044778	73814.667	260850*	276773.33
Pathogenesis-related protein 1-like	0009084	5560766.7	7701533.3	9689300
Homogentisate 1,2-dioxygenase isoform X1	0061270	29817.667	99301.667*	159752.67
Bifunctional nitrilase/nitrile hydratase NIT4B, partial	0024409	265740	447440	590220
Glutamate decarboxylase	0033918	278663.33	517960	746140
Metallo-hydrolase/oxidoreductase	0043116	265497.33	405396.67	763326.67
NADH dehydrogenase [ubiquinone] 1 alpha subcomplex subunit 13-B-like	0042282	0	215540*	212510
N-carbamoylputrescine amidase isoform X2	0059396	290276.67	771750*	1233273.3
Peptidyl-prolyl cis-trans isomerase CYP38, chloroplastic-like isoform X1	0056790	2853166.7	6896200*	9997666.7
Probable L-gulonolactone oxidase 6	0061206	83802	210133.33*	451890

Mean iBAQ: mean value of the calculated protein abundance by intensity based absolute quantification method, n=3. R0min, R1min, R6h: *Racomitrium canescens* samples rehydrated from desiccation for 0 min, 1 min and 6 h, respectively. Asterisks indicate proteins reached the significance and the fold change > 1.2 in R1min or R6h samples, respectively.

Table S4. Differently expressed genes belonging to GO term of vacuolar membrane (GO:0005774) significantly increased at 1 minute after rehydration of desiccated *Racomitrium canescens*.

Unigene ID	Expression trend cluster	Predicted annotation	Expression
0006745	0	chalcone synthase	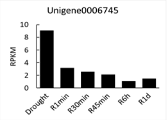
0016186	0	H^+-translocating pyrophosphatase family	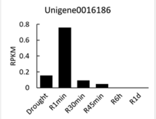
0073914	0	H^+-translocating pyrophosphatase family protein, partial	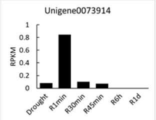
0083215	0	PREDICTED: autophagy-related protein 8C	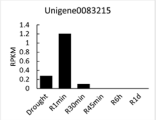
0091530	0	Adenylate cyclase-associated protein	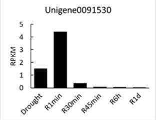
0021137	16	Adenylate cyclase-associated protein	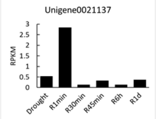
0030480	16	catalase	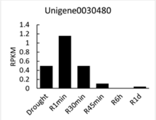
0059566	13	SM/Sec1-family protein	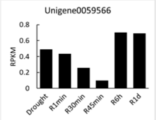
0059704	8	CBL-interacting protein kinase	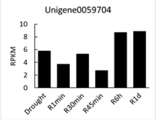
0044014	25	CBL-interacting protein kinase	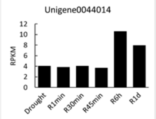
0046204	25	calcineurin B-like calcium binding protein	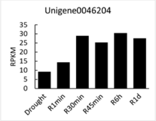

R0min, R1min, R30min, R45min, R6h, R1d: *Racomitrium canescens* rehydrated for 0 min, 1 min, 30 min, 45 min, 6 h, 1 d, respectively. RKPM: reads per kb per million reads.
